# Divergent Syntheses of (-)-Chicanine, (+)-Fragransin A_2_, (+)-Galbelgin, (+)-Talaumidin, and (+)-Galbacin via One-Pot Homologative *γ*-Butyrolactonization

**DOI:** 10.3390/molecules29030701

**Published:** 2024-02-02

**Authors:** Hosam Choi, Jongyeol Han, Joohee Choi, Kiyoun Lee

**Affiliations:** Department of Chemistry, The Catholic University of Korea, Bucheon 14662, Republic of Korea; pzpz1233@gmail.com (H.C.); gkswhdduf@naver.com (J.H.); cholo4015@naver.com (J.C.)

**Keywords:** one-pot homologative γ-butyrolactonization, tetrahydrofuran lignans, (-)-chicanine, (+)-fragransin A_2_, (+)-galbelgin, (+)-talaumidin, (+)-galbacin

## Abstract

In this study, the divergent syntheses of (-)-chicanine, (+)-fragransin A_2_, (+)-galbelgin, (+)-talaumidin, and (+)-galbacin are detailed. In this approach, an early-stage modified Kowalski one-carbon homologation reaction is utilized to construct the central *γ*-butyrolactone framework with the two necessary *β*,*γ*-vicinal stereogenic centers. The two common chiral *γ*-butyrolactone intermediates were designed to be capable for assembling five different optically active tetrahydrofuran lignans from commercially available materials in a concise and effective divergent manner in five to eight steps. These five syntheses are among the shortest and highest-yielding syntheses reported to date.

## 1. Introduction

Lignans and neolignans have been discovered in a number of pharmacologically important and structurally complex natural products [1,2,3]. It is generally understood that lignans have significant biological roles in plants, including protection against herbivores and microbes. It is remarkable how much structural flexibility lignans display with just two phenylpropane (C6-C3) subunits in their molecular framework. Lignans have a broad spectrum of medicinal effects, including anticancer, anti-inflammatory, neuroprotective, antioxidant, and antiviral activities [4,5,6]. These units and their synthetic derivatives are becoming increasingly popular owing to their use in cancer therapy and a variety of other pharmacological effects [7].

Among those lignans, 2,5-diaryl-3,4-dimethyltetrahydrofurans have fueled considerable synthetic efforts owing to their molecular diversity and pharmacological profile [3,8,9,10]. Despite these impressive advances, effective approaches have so far been largely limited in terms of stereoselectivity and the availability of starting materials for lignan synthesis. Importantly, these structures are commonly embedded in larger and more complex structures, resulting in design complications for step-efficient synthesis.

**Figure 1:**
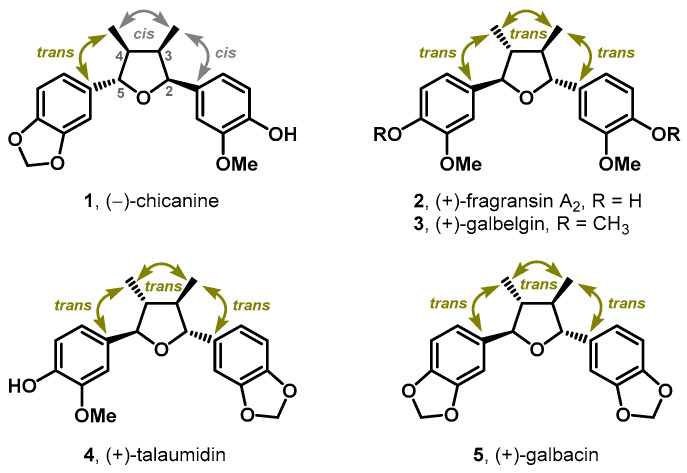
Target molecules featuring 2,5-diaryl-3,4-dimethyltetrahydrofurans.

To further investigate these aspects, herein, we disclose the concise asymmetric syntheses of the tetrahydrofuran lignans, including (-)-chicanine (**1**) [11], (+)-fragransin A_2_ (**2**) [12,13,14,15], (+)-galbelgin (**3**) [14,16,17,18,19,20], (+)-talaumidin (**4**) [18,21,22], and (+)-galbacin (**5**) [16], based on a key reaction we initially disclosed and have recently significantly improved (Figure 1).

**Scheme 1:**
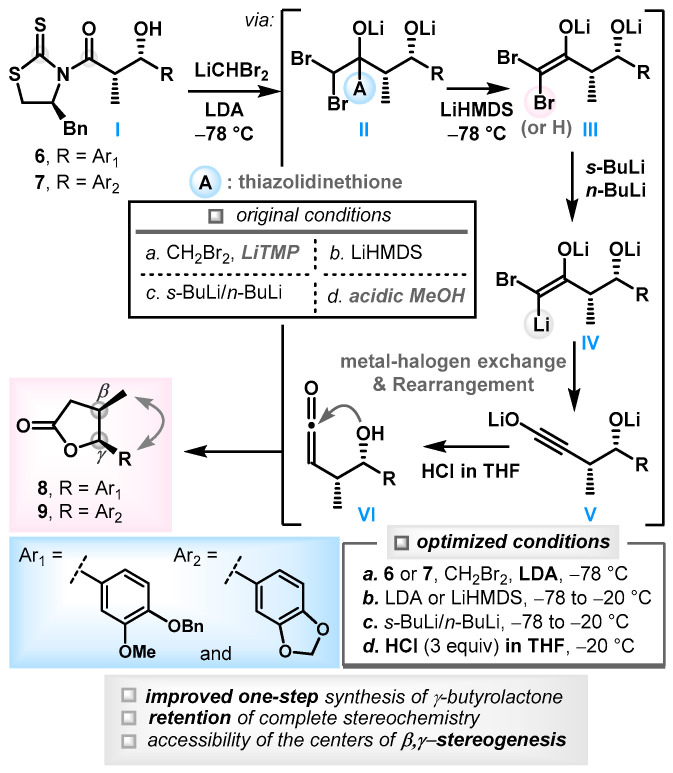
Optimization of a one-pot homologative *γ*-butyrolactonization.

In our previous report, we detailed the stereoselective aldol protocols and the Kowalski ester homologation [23,24,25,26,27] reaction, which we modified for our proposed transformation. This process involved one-carbon homologative *γ*-butyrolactonization and yielded a variety of *γ*-butyrolactones with *β*,*γ*-*cis*-vicinal stereogenic centers [28]. According to the results of our previous study, including the investigation of a range of chiral auxiliaries, the acylthiazolidinethione [29,30] group was determined to be substantially more productive than oxazolidinones or oxazolinethiones, implying that it plays a significant role in the generation of dibromoketone enolate **III** in Scheme 1. Additionally, we demonstrated the synthesis of the challenging *trans*-*γ*-butyrolactone, which can be generated through a sterically hindered *anti*-aldol product [31].

## 2. Results

Although this approach enables the rapid and reliable synthesis of a range of chiral *γ*-butyrolactone frameworks, it also has certain restrictions, resulting in modest to poor chemical yields in some cases. A number of bases and quenching protocols have recently been re-evaluated with the model substrate in an attempt to identify more comprehensive procedures for one-pot one-carbon homologative lactonizations [32].

The optimized conditions involved the replacement of lithium tetramethylpiperidide (LiTMP) with the less hindered and less selective lithium diisopropylamide (LDA) and the substitution of acidic methanol with HCl (3 equiv.) in THF for the quenching process (Scheme 1). These modifications successfully yielded a single diastereomer of *γ*-butyrolactone without the formation of acyclic products, which was a primary issue with the original reaction conditions. 

The following is a simplified representation of the transformation mechanism for the preparation of the key *γ*-butyrolactone skeleton. Our process is initiated by the preparation of dibromomethyllithium from methylene bromide and lithium diisopropylamide (LDA), followed by the addition of **6** and **7** to the dibromomethyllithium solution, resulting in the tetrahedral intermediate, **II**. The subsequent treatment of the intermediate, **II**, with LiHMDS is expected to result in the di- and monobromoketone enolates, **III**. This is followed by the generation of the ynolate anion, **V**, via the metal–halogen exchange rearrangement of **IV**. The chiral *γ*-butyrolactones could then be formed by quenching the ynolate anion, **V**, in THF with HCl (3 equiv.), presumably via the successive intramolecular cyclization of the ketene intermediate, **VI**. It should be noted that one of the key factors in this process for the formation of intermediates **II**–**V** is the addition of reagents at −78 °C, followed by stirring at −20 °C for 90 s, which enables high yields of **8** and **9**. Notably, the stereochemical outcome of the two vicinal stereogenic centers in *γ*-butyrolactones is conserved under these reaction conditions (see the Supplementary Materials for details). It should also be highlighted that by taking advantage of the great stereofacial selectivity of auxiliary-mediated asymmetric aldol processes, all the stereoisomers are accessible.

**Scheme 2:**
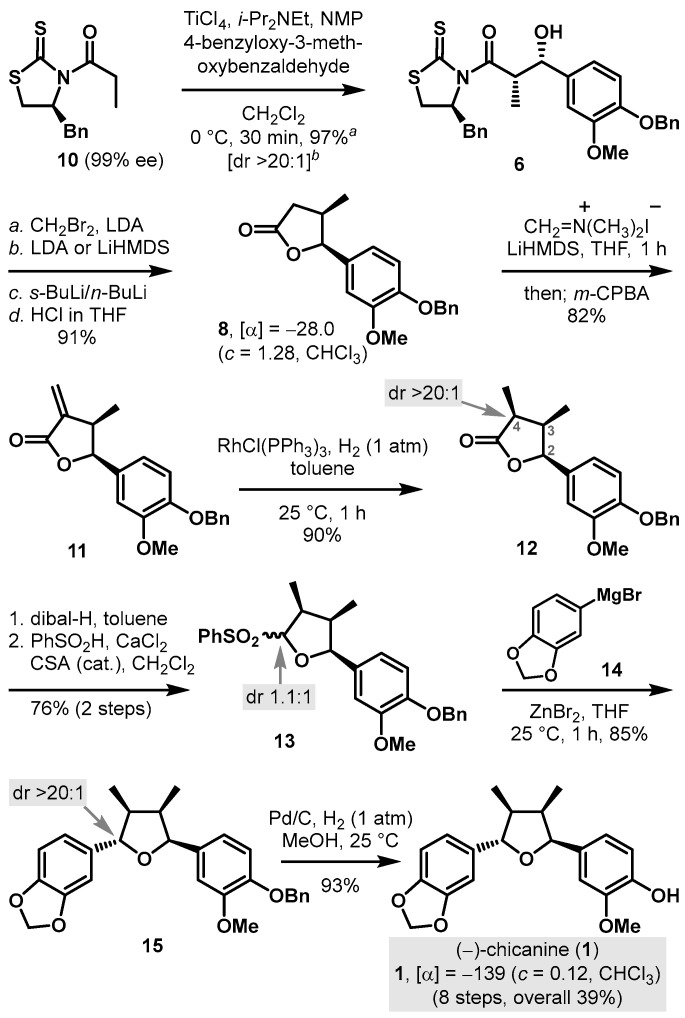
Synthesis of (-)-chicanine (**1**). *^a^*Isolated yield of major diastereomer. *^b^*Dr = diastereomeric ratio = (desired *syn*):(∑ other isomers). The diastereomeric ratio was determined by integrating the ^1^H-NMR spectrum of the crude product.

As outlined in Scheme 2, our synthesis of (-)-chicanine (**1**) began with a highly diastereoselective Evans *syn*-aldol addition reaction, as described by Crimmins [33,34,35]. Commercially available 4-benzyloxy-3-methoxybenzaldehyde was reacted with thiazolidinethione propionate (**10**) in the presence of TiCl_4_ and NMP to provide the desired *syn*-aldol adduct, **6**, in a 97% yield as a single diastereomer. Compound **6** was then subjected to our modified one-pot homologative *γ*-butyrolactonization conditions to afford **8** at an excellent conversion (91%). It is of note that the developed reaction proceeds with the complete retention of the stereochemistry in **8**. Having successfully secured the *γ*-butyrolactone, **8**, we continued our investigation of the biaryl skeleton and completion of the synthesis of (-)-chicanine. To this end, the treatment of **8** with Eschenmoser’s salt (LiHMDS, THF, −78 °C, 1 h) and its subsequent in situ elimination (*m*-CPBA, CH_2_Cl_2_, 25 °C, 5 min) afforded the *α*-*exo*-methylene lactone, **11**, in a 76% yield. The subsequent diastereoselective hydrogenation was most effectively carried out using Wilkinson’s catalyst (RhCl(PPh_3_)_3_, H_2_, 25 °C, 1 h) in toluene. At this juncture, the C-4 methyl group determines the facial selectivity, resulting in the formation of **12** as a single diastereomer with the optimal conversion (90%, dr > 20:1). 

Having successfully produced **12**, our next focus was the crucial arylation of methylenedioxybenzene. The primary issue we encountered in the preparation of the stereoselective 2,3-*cis*-3,4-*cis*-4,5-*trans*-tetrahydrofuran was the occurrence of epimerization. To prevent this, we adopted Ley’s procedure [36,37,38], which involves the introduction of an organozinc reactant to the 2-benzenesulfonyl cyclic ether, **13**, which was obtained in a 76% yield by the reduction of **12** with DIBALH, followed by treatment with PhSO_2_H and CSA in two steps. The subsequent nucleophilic substitution reaction of **13** with (1,2-methylenedioxyphenyl)zinc (II) bromide, generated in situ from (1,2-methylenedioxyphenyl)magnesium bromide and ZnBr_2_, resulted in an 85% yield of 2,3-*cis*-3,4-*cis*-4,5-*trans*-tetrahydrofuran, **15**. It should be noted that we did not detect any other diastereomers at this stage. The final deprotection of the benzyl group (Pd/C, H_2_, MeOH, 93%) afforded natural (-)-chicanine (**1**, [α]_D_ −139 (*c* 0.12, CHCl_3_) vs. reported [α]_D_ −134.3 (*c* 1.01, CHCl_3_)) [11,39], which proved to be identical in all respects to the natural product. This demonstrates that the approach outlined in this report enabled the synthesis of (-)-chicanine in eight steps and in an overall yield of 39% from commercially available thiazolidinethione propionate, **10**.

**Scheme 3:**
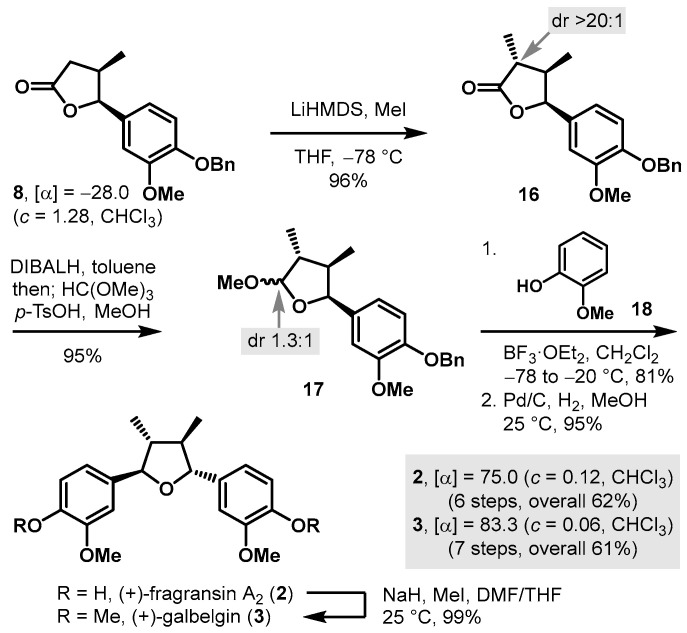
Syntheses of (+)-fragransin A_2_ (**2**) and (+)-galbelgin (**3**).

An additional benefit of the current approach is that the same intermediate, **8**, can also be used to access the straightforward synthesis of naturally occurring (+)-fragransin A_2_ (**2**) and (+)-galbelgin (**3**) (Scheme 3). The biological profiles of these compounds, which includes antioxidant and antiviral activities, as well as the all *trans* 2,5-diaryl-3,4-dimethyltetrahydrofuran structural feature, have drawn the attention of organic chemists to pursue a stereoselective synthesis of target molecules.

With the requisite γ-butyrolactone, **8**, in hand, an asymmetric *α*-methylation (LiHMDS, MeI, THF, −78 °C) cleanly proceeded to provide 3,4-dimethyl-5-aryldihydrofuran-2(3*H*)-one (**16**) in a 96% yield as a single diastereomer. At this stage, we envisioned that stereoselective 2,3-*trans*-3,4-*trans*-4,5-*trans*-tetrahydrofuran embedded in **2** and **3** would be accessed by the BF_3_∙OEt_2_-promoted epimerization of the cyclic methyl acetal or hemiketal [8,14,40]. To this end, the lactone, **16**, was converted to the cyclic methyl acetal, **17**, by one-pot reduction (DIBALH) and acetalization in a 95% yield. As predicted, the subsequent Friedel–Crafts-type arylation reaction conditions (**18**, BF_3_∙OEt_2_, CH_2_Cl_2_, from −78 to −20 °C) proceeded with epimerization at the C2 position of **17** to provide the desired 2,3-*trans*-3,4-*trans*-4,5-*trans*-tetrahydrofuran as a single diastereomer. The exploration of the Friedel–Crafts-type arylation by employing the cyclic hemiketal, generated by reduction with DIBALH, was less productive (54%, see the Supplementary Materials). 

Finally, the removal of the Bn protecting group enabled the completion of (+)-fragransin A_2_ (**2**, [α]_D_ +75.0 (*c* 0.12, CHCl_3_) vs. reported [α]_D_ +86.9 (*c* 0.88, CHCl_3_)) [15]. Furthermore, the methylation of the two corresponding phenolic groups in **2** provided a straightforward access to (+)-galbelgin (**3**, [α]_D_ +83.3 (*c* 0.06, CHCl_3_) vs. [α]_D_ +80.0 (*c* 0.5, CHCl_3_)) [20], resulting in only six steps (for (+)-fragransin A_2_) and seven steps (for (+)-galbelgin) in remarkably high yields (61–62% overall), demonstrating the efficiency of our approaches.

**Scheme 4:**
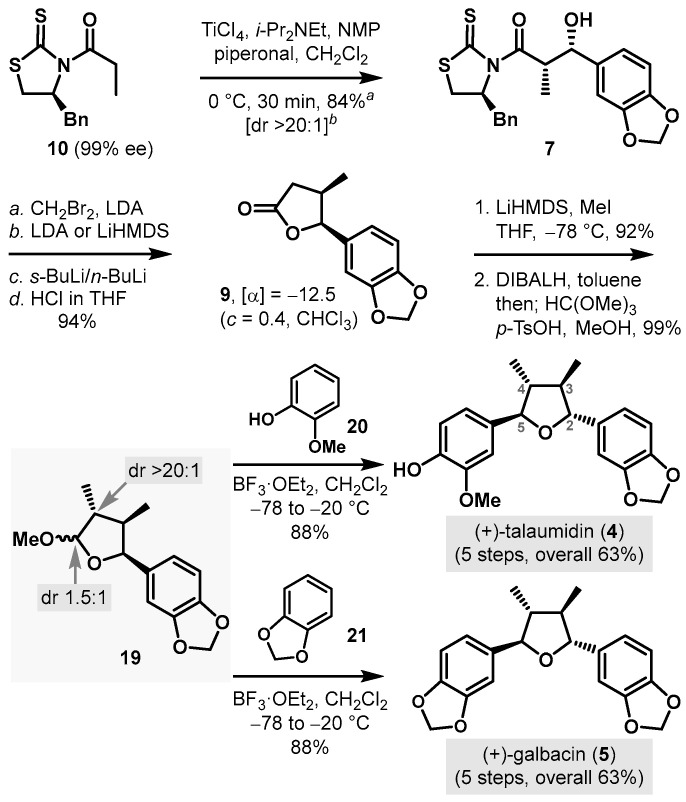
Syntheses of (+)-talaumidin (**4**) and (+)-galbacin (**5**). *^a^*Isolated yield of major diastereomer. *^b^*Dr = diastereomeric ratio = (desired *syn*):(∑ other isomers).

Another common *γ*-butyrolactone intermediate enabled the synthesis of two additional 2,5-diaryl-3,4-dimethyltetrahydrofuran lignans, named (+)-talaumidin (**4**) and (+)-galbacin (**5**). The syntheses of **4** and **5** began with the preparation of the key *γ*-butyrolactone, **9** (Scheme 4). The diastereoselective aldol addition of the chlorotitanium enolate of thiazolidinethione propionate, **10**, with 3,4-methylenedioxybenzaldehyde yielded the *syn*-aldol adduct, **7**, in an 84% yield, which was subjected to our modified one-carbon homologative lactonization to provide the desired γ-butyrolactone, **9**, in a 94% yield. The subsequent asymmetric *α*-methylation of **9** (LiHMDS, MeI, THF, −78 °C) provided the desired 3,4-*trans*-dimethyltetrahydrofuran with excellent conversion and diastereoselectivity (92%, dr > 20:1). By employing one-pot reduction and acetalization, the lactone was cleanly converted to the cyclic methyl acetal, **19** (99%), and the ensuing Friedel–Crafts-type arylation reaction conditions (**20**, BF_3_∙OEt_2_, CH_2_Cl_2_, from −78 to −20 °C, 88%) proceeded to give (+)-talaumidin (**4**, [α]_D_ +75.9 (*c* 0.16, CHCl_3_) vs. reported [α]_D_ +88.3 (*c* 2.1, CHCl_3_)) [39] and [α]_D_ +76.3 (*c* 0.28, CHCl_3_) [18]. Additionally, the synthesis of (+)-galbacin (**5**) was successfully achieved employing a Friedel–Crafts-type arylation process with **19** and **21**, resulting in a yield of 88% ([α]_D_ +121 (*c* 0.22, CHCl_3_) vs. [α]_D_ +110 (*c* 1.0, CHCl_3_)) [20], and for which the properties in all respects proved to be identical with those of the known synthetic sample of the natural product. Both syntheses, which are among the shortest documented to date, comprised five sequential steps and resulted in yields of 63%.

## 3. Materials and Methods

### 3.1. General Information

All the reactions were conducted in oven-dried glassware under nitrogen. Unless otherwise stated, all the reagents were purchased from Sigma-Aldrich (St. Louis, MO, USA); Acros; or Fisher (Hampton, NH, USA) and were used without further purification. All the solvents were ACS grade or higher and purified before use. Dichloromethane (CH_2_Cl_2_) and dimethylformamide (DMF) were distilled from CaH_2_. Tetrahydrofuran (THF) was distilled from sodium benzophenone. Methanol (MeOH) was distilled from Mg/I_2_. Analytical thin-layer chromatography (TLC) was performed with glass-backed silica gel (60 Å) plates and a fluorescent indicator (Whatman, St. Louis, MO, USA). Visualization was accomplished by UV irradiation at 254 nm and/or by staining with ceric ammonium molybdate, phosphomolybdic acid in EtOH, or *p*-anisaldehyde solution. Flash column chromatography was performed using silica gel (particle size: 70–230 mesh, ASTM). All the ^1^H-NMR and ^13^C-NMR spectra were recorded at 298 K on a Bruker Avance III HD 500 MHz (Bruker Corporation, Billerica, MA, USA) spectrometer in CDCl_3_ using the signal of the residual CHCl_3_ as an internal standard. All the NMR *δ* values are given in ppm, and all the *J* values are in Hz. Optical rotation values were measured with a Rudolph Research Analytical (AUTOPOL II, Hackettstown, NJ, USA) polarimeter.

### 3.2. Synthesis of (-)-Chicanine (**1**)

(2*S*,3*S*)-1-((*S*)-4-Benzyl-2-thioxothiazolidin-3-yl)-3-(4-(benzyloxy)-3-methoxyphenyl)-3-hydroxy-2-methylpropan-1-one (**6**): To a cooled (0 °C) solution of (*S*)-1-(4-benzyl-2-thioxothiazolidin-3-yl)propan-1-one, **10**, (670 mg, 2.52 mmol) in CH_2_Cl_2_ (13 mL, 0.2 M), titanium(IV) chloride (2.8 mL, 1.0 M in CH_2_Cl_2_, 2.8 mmol, 1.1 equiv.) was added. After being stirred for 15 min at the same temperature, *i*-Pr_2_NEt (0.53 mL, 3.0 mmol, 1.2 equiv.) was added dropwise, and the reaction mixture was stirred for 40 min at 0 °C. NMP (0.49 mL, 5.1 mmol, 2 equiv.) was added, and the reaction mixture was stirred for an additional 10 min. 4-Benzyloxy-3-methoxybenzaldehyde (1.22 g, 5.04 mmol, 2 equiv.) in CH_2_Cl_2_ (10 mL) was added to the enolate. After being stirred for 30 min, the reaction mixture was quenched with saturated aqueous NH_4_Cl (20 mL) and diluted with CH_2_Cl_2_ (10 mL). The layers were separated, and the aqueous layer was extracted with CH_2_Cl_2_ (20 mL × 3). The combined organic layers were washed with brine (40 mL × 1), dried over anhydrous Na_2_SO_4_, filtered, and concentrated in vacuo. The residue was purified by column chromatography (SiO_2_, 25% EtOAc/hexane) to provide **6** (1.24 g, 97%) as a yellow foam: [α]_D_^25^ +138 (*c* 1.27, CHCl_3_); ^1^H-NMR (500 MHz, CDCl_3_) δ 7.31–7.37 (m, 4H); 7.26–7.30 (m, 1H); 7.21–7.25 (m, 2H); 7.16–7.20 (m, 3H); 6.96 (d, *J* = 1.7 Hz, 1H); 6.77 (d, *J* = 8.2 Hz, 1H); 6.71 (dd, *J* = 8.2, 1.8 Hz, 1H); 5.10 (ABX, 2H, *J* = 12.7 Hz, Δν = 11.9 Hz); 4.82 (p, *J* = 6.6 Hz, 1H); 4.66 (d, *J* = 7.8 Hz, 1H); 4.47 (ddd, *J* = 10.5, 6.5, 3.9 Hz, 1H); 3.88 (s, 3H); 3.10 (dd, *J* = 13.2, 3.6 Hz, 1H); 2.86 (dd, *J* = 13.1, 10.8 Hz, 1H); 2.63 (br s, 1H); 2.47 (d, *J* = 11.3 Hz, 1H); 2.40 (dd, *J* = 11.3, 6.8 Hz, 1H); 1.39 (d, *J* = 6.6 Hz, 3H); ^13^C-NMR (125 MHz, CDCl_3_) δ 201.3, 176.8, 149.5, 147.4, 136.7, 136.2, 134.8, 129.2, 128.7, 128.3, 127.8, 127.1, 127.0, 118.5, 113.2, 109.3, 76.8, 70.5, 68.9, 55.8, 46.6, 36.4, 32.1, and 13.0; HRMS (Q–TOF) *m*/*z*: 506.1460 ((M–H)^+^, C_28_H_28_NO_4_S_2_ requires 506.1460). 

(4*R*,5*S*)-5-(4-(Benzyloxy)-3-methoxyphenyl)-4-methyldihydrofuran-2(3H)-one (**8**): To a cooled (−78 °C) solution of *i*-Pr_2_NH (1.1 mL, 7.8 mmol, 4.4 equiv.) in THF (4.5 mL, 0.4 M), *n*-BuLi (4.5 mL, 1.6 M in hexane, 7.1 mmol, 4 equiv.) was added, and the reaction mixture was stirred for 15 min at 0 °C before it was cooled to −78 °C. CH_2_Br_2_ (7.8 mL, 1.0 M in THF, 7.8 mmol, 4.4 equiv.) and **6** (904 mg, 1.78 mmol) in THF (7.8 mL) were added dropwise to the above LDA solution. After being stirred for 10 min at −78 °C, the reaction mixture was added to LiHMDS (7.1 mL, 1.0 M in THF, 7.1 mmol, 4 equiv.). After being stirred for 5 min at −78 °C, the reaction mixture was stirred for 90 s at −20 °C before it was cooled to −78 °C. *s*-BuLi (5.1 mL, 1.4 M in cyclohexane, 7.1 mmol, 4 equiv.) was added dropwise, and the reaction mixture was stirred for 90 s at −20 °C and cooled to −78 °C, followed by the dropwise addition of *n*-BuLi (4.5 mL, 1.6 M in hexane, 7.1 mmol, 4 equiv.). After being stirred for 5 min, the reaction mixture was stirred for 90 s at −20 °C, warmed to 25 °C in a water bath, and stirred for 30 min before it was added to a solution of HCl (0.44 mL, 5.3 mmol, 3 equiv.) in THF (59 mL, 0.03 M) at −20 °C. After being stirred for 5 min at −20 °C, the reaction mixture was quenched with the addition of saturated aqueous NaHCO_3_ (30 mL) and diluted with EtOAc (30 mL). The layers were separated, and the aqueous layer was extracted with EtOAc (30 mL × 3). The combined organic layers were washed with brine (60 mL × 1), dried over anhydrous Na_2_SO_4_, filtered, and concentrated in vacuo. The residue was purified by column chromatography (SiO_2_, 33% EtOAc/hexane) to provide **8** (506 mg, 91%) as a colorless oil: [α]_D_^25^ −28.0 (*c* 1.28, CHCl_3_); ^1^H-NMR (500 MHz, CDCl_3_) δ 7.42–7.45 (m, 2H); 7.34–7.38 (m, 2H); 7.28–7.32 (m, 1H); 6.88 (d, *J* = 8.3 Hz, 1H); 6.77 (d, *J* = 2.0 Hz, 1H); 6.68–6.71 (m, 1H); 5.53 (d, *J* = 5.7 Hz, 1H); 5.15 (s, 2H); 3.88 (s, 3H); 2.79–2.85 (m, 2H); 2.31–2.37 (m, 1H); 0.70 (d, *J* = 7.0 Hz, 3H); ^13^C-NMR (125 MHz, CDCl_3_) δ 176.8, 149.6, 147.8, 136.9, 129.1, 128.5, 127.8, 127.2, 117.6, 113.8, 109.1, 83.9, 71.0, 56.1, 37.1, 35.1, and 15.1; HRMS (Q–TOF) *m*/*z*: 335.1260 ((M + Na)^+^, C_19_H_20_NaO_4_ requires 335.1259). 

(4*R*,5*S*)-5-(4-(Benzyloxy)-3-methoxyphenyl)-4-methyl-3-methylenedihydrofuran-2(3H)-one (**11**): To a cooled (−78 °C) solution of **8** (229 mg, 0.733 mmol) in THF (3.7 mL, 0.2 M), LiHMDS (1.5 mL, 1.0 M in THF, 1.5 mmol, 2 equiv.) was added. After being stirred for 1 h, the reaction mixture was added to Eschenmoser’s salt (407 mg, 2.20 mmol, 3 equiv.) and stirred for 10 min at −78 °C before it was quenched with the addition of saturated aqueous NH_4_Cl (5 mL) and diluted with Et_2_O (10 mL). The layers were separated, and the aqueous layer was extracted with Et_2_O (10 mL × 3). The combined organic layers were washed with brine (20 mL × 1), dried over anhydrous Na_2_SO_4_, filtered, and concentrated in vacuo. The residue was dissolved in CH_2_Cl_2_ (9.2 mL, 0.08 M) and saturated NaHCO_3_ (4.6 mL, 0.16 M). *m*-CPBA (190 mg, 1.10 mmol, 1.5 equiv.) was added to the above solution. After being stirred for 5 min at 0 °C, the reaction mixture was diluted with saturated aqueous NaHCO_3_ (10 mL). The layers were separated, and the aqueous layer was extracted with CH_2_Cl_2_ (15 mL × 3). The combined organic layers were washed with brine (20 mL × 1), dried over anhydrous Na_2_SO_4_, filtered, and concentrated in vacuo. The residue was purified by column chromatography (SiO_2_, 13% EtOAc/hexane) to provide **11** (195 mg, 82%) as a colorless oil: [α]_D_^25^ +30.0 (*c* 1.73, CHCl_3_); ^1^H-NMR (500 MHz, CDCl_3_) δ 7.41−7.44 (m, 2H); 7.34−7.38 -(m, 2H); 7.28−7.32 (m, 1H); 6.86 (d, *J* = 8.2 Hz, 1H); 6.67 (d, *J* = 2.0 Hz, 1H); 6.64 (dd, *J* = 8.3, 1.9 Hz, 1H); 6.32 (d, *J* = 2.8 Hz, 1H); 5.57 (d, *J* = 2.6 Hz, 1H); 5.55 (d, *J* = 8.0 Hz, 1H); 5.14 (s, 2H); 3.86 (s, 3H); 3.35−3.43 (m, 1H); 0.81 (d, *J* = 7.1 Hz, 3H); ^13^C-NMR (125 MHz, CDCl_3_) δ 170.6, 149.6, 148.1, 140.2, 136.8, 129.3, 128.5, 127.9, 127.3, 121.6, 118.5, 113.7, 109.5, 82.1, 71.0, 56.0, 39.0, and 15.3; HRMS (Q–TOF) *m*/*z*: 325.1444 ((M + H)^+^, C_20_H_21_O_4_ requires 325.1440).

*(3S,4R,5S)-5-(4-(Benzyloxy)-3-methoxyphenyl)-3,4-dimethyldihydrofuran-2(3H)-one* (**12**): To a solution of **11** (92.0 mg, 0.284 mmol) in toluene (2.8 mL, 0.1 M), RhCl(PPh_3_)_3_ (79 mg, 0.085 mmol, 30 mol%) was added, and the reaction mixture was hydrogenated at 1 atm. After being stirred for 1 h at 25 °C, the reaction mixture was concentrated in vacuo and purified by column chromatography (SiO_2_, 13% EtOAc/hexane) to provide **12** (83.1 mg, 90%) as a colorless oil: [α]_D_^20^ −50.0 (*c* 0.14, CHCl_3_); ^1^H-NMR (500 MHz, CDCl_3_) δ 7.43 (d, *J* = 7.3 Hz, 2H); 7.34–7.38 (m, 2H); 7.27–7.31 (m, 1H); 6.88 (d, *J* = 8.3 Hz, 1H); 6.82 (d, *J* = 1.8 Hz, 1H); 6.72 (dd, *J* = 8.3, 1.5 Hz, 1H); 5.45 (d, *J* = 5.0 Hz, 1H); 5.14 (s, 2H); 3.88 (s, 3H); 2.94–3.01 (m, 1H); 2.68–2.76 (m, 1H); 1.21 (d, *J* = 7.2 Hz, 3H); 0.55 (d, *J* = 7.3 Hz, 3H); ^13^C-NMR (125 MHz, CDCl_3_) δ 179.0, 149.6, 147.6, 136.9, 129.2, 128.5, 127.8, 127.2, 117.4, 113.8, 108.9, 82.1, 71.0, 56.0, 41.0, 40.0, 10.0, and 9.4; HRMS (Q–TOF) *m*/*z*: 327.1609 ((M + H)^+^, C_20_H_23_O_4_ requires 327.1596). 

*(2S,3R,4S)-2-(4-(Benzyloxy)-3-methoxyphenyl)-3,4-dimethyl-5-(phenylsulfonyl)tetrahydrofuran* (**13**): [DIBALH reduction]: To a cooled (−78 °C) solution of **12** (92.7 mg, 0.284 mol) in toluene (2.8 mL, 0.1 M), DIBALH (0.31 mL, 1.0 M in toluene, 0.31 mmol, 1.1 equiv.) was added. After being stirred for 10 min at −78 °C, the reaction mixture was quenched with MeOH (0.5 mL), followed by the addition of aqueous Rochelle’s salt solution (15 mL), and diluted with Et_2_O (15 mL). The resulting mixture was stirred for 6 h at 25 °C. The layers were separated, and the aqueous layer was extracted with Et_2_O (10 mL × 3). The combined organic layers were washed with brine (15 mL × 1), dried over anhydrous Na_2_SO_4_, filtered, and concentrated in vacuo. The residue was filtered through a pad of silica gel (20% EtOAc/hexane) and concentrated in vacuo to provide a crude mixture, which was employed in the next step without further purification. [Sulfonylation]: To a solution of the above crude mixture in CH_2_Cl_2_ (3.6 mL, 0.08 M), PhSO_2_H (80.8 mg, 0.568 mmol, 2 equiv.), CaCl_2_ (94.6 mg, 0.852 mmol, 3 equiv.), and CSA (0.28 mL, 0.1 M in CH_2_Cl_2_, 0.028 mmol, 10 mol%) were added. After being stirred for 2 h at 25 °C, the reaction mixture was quenched with saturated aqueous NaHCO_3_ (5 mL) and diluted with EtOAc (5 mL). The layers were separated, and the aqueous layer was extracted with EtOAc (5 mL × 3). The combined organic layers were washed with brine (10 mL × 1), dried over anhydrous Na_2_SO_4_, filtered, and concentrated in vacuo. The residue was purified by column chromatography (SiO_2_, 10% EtOAc/hexane) to provide a 1.1:1 anomeric mixture of sulfonate, **13** (97.5 mg, 76%) as a colorless oil: ^1^H-NMR (500 MHz, CDCl_3_) δ 7.96–7.99 (m, 2H); 7.91–7.94 (m, 2H); 7.63–7.67 (m, 1H); 7.59–7.63 (m, 1H); 7.54 (t, *J* = 7.7 Hz, 2H); 7.50 (t, *J* = 7.7 Hz, 2H); 7.46 (d, *J* = 7.4 Hz, 2H); 7.42 (d, *J* = 7.4 Hz, 2H); 7.34–7.40 (m, 5H); 7.28–7.33 (m, 2H); 6.87 (dd, *J* = 8.2, 1.7 Hz, 1H); 6.84 (d, *J* = 8.2 Hz, 1H); 6.81 (d, *J* = 8.3 Hz, 1H); 6.72 (d, *J* = 1.7 Hz, 1H); 6.64 (dd, *J* = 8.2, 1.7 Hz, 1H); 5.28 (d, *J* = 4.5 Hz, 1H); 5.17 (s, 2H); 5.13 (s, 2H); 4.62 (d, *J* = 8.0 Hz, 1H); 4.60 (d, *J* = 1.3 Hz, 1H); 4.54 (d, *J* = 10.5 Hz, 1H); 3.98 (s, 3H); 3.93 (s, 3H); 3.20–3.30 (m, 2H); 2.68–2.77 (m, 1H); 2.42–2.50 (m, 1H); 1.30 (d, *J* = 7.0 Hz, 3H); 1.20 (d, *J* = 7.4 Hz, 3H); 0.88 (d, *J* = 6.9 Hz, 3H); 0.53 (d, *J* = 7.2 Hz, 3H); ^13^C-NMR (125 MHz, CDCl_3_) δ 150.0, 149.4, 148.4, 147.3, 137.5, 137.4, 137.2, 137.1, 133.8, 132.0, 131.0, 129.4, 129.093, 129.088, 129.0, 128.58, 128.55, 127.9, 127.8, 127.29, 127.27, 120.3, 118.1, 113.6, 113.1, 110.7, 109.8, 100.0, 97.8, 89.9, 85.8, 71.1, 71.0, 56.1, 43.8, 42.3, 38.6, 38.4, 14.6, 14.5, 10.3, and 9.4; HRMS (EI) *m*/*z*: 452.1658 ((M)^+^, C_26_H_28_O_5_S requires 452.1657). 

5-((2*S*,3*S*,4*R*,5*S*)-5-(4-(Benzyloxy)-3-methoxyphenyl)-3,4-dimethyltetrahydrofuran-2-yl)benzo[*d*][1,3]dioxole (**15**): To a solution of 3,4-methylenedioxybromobenzene (59.2 mg, 0.294 mmol, 3.2 equiv.) and Mg turnings (6.7 mg, 0.276 mmol, 3 equiv.) in THF (0.9 mL, 0.1 M), I_2_ (46 μL, 0.1 M in THF, 4.6 μmol, 5 mol%) was added, and the resulting mixture was stirred for 30 min at 80 °C (the oil bath temperature) to afford **14** and then treated with ZnBr_2_ (1.5 mL, 0.2 M in THF, 0.29 mmol, 3.2 equiv.) via a cannula at 25 °C, and the resulting mixture was stirred for 30 min at 25 °C before it was added to **13** (41.6 mg, 0.092 mmol) in THF (0.9 mL, 0.1 M). After being stirred for 1 h at 25 °C, the reaction mixture was quenched with saturated aqueous NH_4_Cl (5 mL) and diluted with EtOAc (5 mL). The layers were separated, and the aqueous layer was extracted with EtOAc (5 mL × 3). The combined organic layers were washed with brine (10 mL × 1), dried over anhydrous Na_2_SO_4_, filtered, and concentrated in vacuo. The residue was purified by column chromatography (SiO_2_, 10% EtOAc/hexane) to provide **15** (33.8 mg, 85%) as a colorless oil: [α]_D_^25^ −86.6 (*c* 0.15, CHCl_3_); ^1^H-NMR (500 MHz, CDCl_3_) δ 7.44 (d, *J* = 7.4 Hz, 2 H); 7.34–7.38 (m, 2H); 7.27–7.31 (m, 1H); 6.93 (dd, *J* = 4.3, 1.6 Hz, 2H); 6.85 (d, *J* = 8.2 Hz, 1H); 6.82 (dd, *J* = 8.0, 1.5 Hz, 1H); 6.77 (dd, *J* = 8.0, 2.6 Hz, 2H); 5.94 (ABq, 2H, *J* = 1.4 Hz, Δν = 3.1 Hz); 5.42 (d, *J* = 4.4 Hz, 1H); 5.14 (s, 2H); 4.62 (d, *J* = 9.3 Hz, 1H); 3.89 (s, 3H); 2.36–2.46 (m, 2H); 1.00 (d, *J* = 6.5 Hz, 3H); 0.61 (d, *J* = 7.0 Hz, 3H); ^13^C-NMR (125 MHz, CDCl_3_) δ 149.4, 147.8, 146.89, 146.85, 137.3, 137.2, 133.8, 128.5, 127.7, 127.3, 119.5, 118.0, 113.8, 109.8, 107.9, 106.4, 100.9, 85.7, 84.7, 71.1, 56.0, 47.6, 43.4, 11.8, and 9.4; HRMS (Q–TOF) *m*/*z*: 433.2020 ((M + H)^+^, C_27_H_29_O_5_ requires 433.2015). 

(-)-Chicanine (**1**): To a solution of **15** (24.8 mg, 0.057 mol) in MeOH (1.4 mL, 0.04 M), Pd/C (10%, 124 mg) was added, and the reaction mixture was hydrogenated at 1 atm. After being stirred for 2 h at 25 °C, the reaction mixture was filtered through a pad of celite and concentrated in vacuo. The residue was purified by column chromatography (SiO_2_, 25% EtOAc/hexane) to provide natural (-)-chicanine (**1**, 18.1 mg, 93%) as a colorless oil for which the spectral data were identical to those of the known synthetic **1 [11,39]**: [α]_D_^25^ –139 (*c* 0.12, CHCl_3_) vs. [α]_D_^25^ −134.3 (*c* 1.01, CHCl_3_) [39]; ^1^H-NMR (500 MHz, CDCl_3_) δ 6.92 (dd, *J* = 5.8, 1.5 Hz, 2H); 6.88 (d, *J* = 8.1 Hz, 1H); 6.82 (dd, *J* = 8.0, 1.5 Hz, 1H); 6.78 (d, *J* = 7.9 Hz, 1H); 6.77 (dd, *J* = 8.2, 1.6 Hz, 1H); 5.94 (ABq, 2H, *J* = 1.3 Hz, Δν = 3.2 Hz); 5.55 (s, 1H); 5.43 (d, *J* = 4.3 Hz, 1H); 4.62 (d, *J* = 9.3 Hz, 1H); 3.88 (s, 3H); 2.36–2.46 (m, 2H); 0.99 (d, *J* = 6.4 Hz, 3H); 0.61 (d, *J* = 7.0 Hz, 3H); ^13^C-NMR (125 MHz, CDCl_3_) δ 147.8, 146.9, 146.2, 144.3, 137.2, 132.5, 119.5, 118.8, 113.9, 108.7, 108.0, 106.4, 100.9, 85.7, 84.8, 55.9, 47.6, 43.4, 11.8, and 9.4; HRMS (Q–TOF) *m*/*z*: 343.1559 ((M + H)^+^, C_20_H_23_O_5_ requires 343.1545). 

### 3.3. Synthesis of (+)-Fragransin A_2_ (**2**) and (+)-Galbelgin (**3**)

(3*R*,4*R*,5*S*)-5-(4-(Benzyloxy)-3-methoxyphenyl)-3,4-dimethyldihydrofuran-2(3H)-one (**16**): To a cooled (−78 °C) solution of **8** (187 mg, 0.599 mmol) in THF (6.0 mL, 0.1 M), LiHMDS (1.2 mL, 1.0 M in THF, 1.2 mmol, 2 equiv.) was added. After being stirred for 1 h, the reaction mixture was added to MeI (56.0 μL, 0.899 mmol, 1.5 equiv.) and stirred for 10 min at −78 °C before it was quenched with the addition of saturated aqueous NH_4_Cl (10 mL) and diluted with EtOAc (10 mL). The layers were separated, and the aqueous layer was extracted with EtOAc (10 mL × 3). The combined organic layers were washed with brine (20 mL), dried over anhydrous Na_2_SO_4_, filtered, and concentrated in vacuo. The residue was purified by column chromatography (SiO_2_, 25% EtOAc/hexane) to provide **16** (187 mg, 96%) as a colorless oil: [α]_D_^25^ +28.5 (*c* 1.72, CHCl_3_); ^1^H-NMR (500 MHz, CDCl_3_) δ 7.42 (d, *J* = 7.3 Hz, 2H); 7.33−7.37 (m, 2H); 7.27−7.31 (m, 1H); 6.87 (d, *J* = 8.2 Hz, 1H); 6.66 (d, *J* = 1.9 Hz, 1H); 6.64 (dd, *J* = 8.2, 1.8 Hz, 1H); 5.48 (d, *J* = 7.6 Hz, 1H); 5.13 (s, 2H); 3.86 (s, 3H); 2.41−2.49 (m, 1H); 2.30−2.37 (m, 1H); 1.27 (d, *J* = 7.1 Hz, 3H); 0.75 (d, *J* = 6.9 Hz, 3H); ^13^C-NMR (125 MHz, CDCl_3_) δ 179.7, 149.5, 147.9, 136.8, 129.1, 128.4, 127.8, 127.2, 118.0, 113.6, 109.4, 82.3, 70.9, 56.0, 42.3, 39.9, 14.5, and 13.6; HRMS (Q–TOF) *m*/*z*: 327.1612 ((M + H)^+^, C_20_H_23_O_4_ requires 327.1596). 

(2*S*,3*R*,4*R*)-2-(4-(Benzyloxy)-3-methoxyphenyl)-5-methoxy-3,4-dimethyltetrahydrofuran **(17):** To a cooled (−78 °C) solution of **16** (18.3 mg, 0.073 mmol) in toluene (1.8 mL, 0.04 M), DIBALH (80 μL, 1.0 M in toluene, 0.080 mmol, 1.1 equiv.) was added. After being stirred for 10 min at −78 °C, the reaction mixture was diluted with MeOH (3.7 mL, 0.02 M) before it was added to HC(OMe)_3_ (80 μL, 0.73 mmol, 10 equiv.) and *p*-TsOH (6.3 mg, 0.037 mmol, 50 mol%). After being stirred for 12 h at 25 °C, the reaction mixture was quenched with saturated aqueous NaHCO_3_ (10 mL) and diluted with EtOAc (10 mL). The layers were separated, and the aqueous layer was extracted with EtOAc (10 mL × 3). The combined organic layers were washed with brine (15 mL × 1), dried over anhydrous Na_2_SO_4_, filtered, and concentrated in vacuo. The residue was purified by column chromatography (SiO_2_, 10% EtOAc/hexane) to provide a 1.3:1 anomeric mixture of cyclic methyl acetal, **17**, (18.4 mg, 95%) as a colorless oil: For the major diastereomer: ^1^H-NMR (500 MHz, CDCl_3_) δ 7.42–7.45 (m, 2H); 7.33–7.38 (m, 2H); 7.30 (d, *J* = 7.1 Hz, 1H); 6.84 (d, *J* = 8.2 Hz, 1H); 6.74 (d, *J* = 1.7 Hz, 1H); 6.67 (dd, *J* = 8.2, 1.7 Hz, 1H); 5.12–5.17 (m, 2H); 5.04 (d, *J* = 4.7 Hz, 1H); 3.88, (s, 3H); 3.43 (s, 3H); 2.24–2.33 (m, 1H); 1.81–1.91 (m, 1H); 1.03 (d, *J* = 6.8 Hz, 3H); 0.56 (d, *J* = 7.0 Hz, 3H); for the minor diastereomer: ^1^H-NMR (500 MHz, CDCl_3_) δ 7.42–7.45 (m, 2H); 7.33–7.38 (m, 2H); 7.28 (d, *J* = 6.9 Hz, 1H); 7.06 (d, *J* = 1.7 Hz, 1H); 6.83 (d, *J* = 8.2 Hz, 1H); 6.76 (dd, *J* = 8.3, 1.7 Hz, 1H); 5.12–5.17 (m, 2H); 4.75 (d, *J* = 5.1 Hz, 1H); 3.90, (s, 3H); 3.56 (s, 3H); 2.10–2.18 (m, 1H); 1.81–1.91 (m, 1H); 1.09 (d, *J* = 6.9 Hz, 3H); 0.64 (d, *J* = 7.0 Hz, 3H); for **17**: ^13^C-NMR (125 MHz, CDCl_3_) δ 149.23, 149.18, 147.1, 146.9, 137.3, 137.2, 133.9, 133.8, 128.4, 127.67, 127.65, 127.23, 127.20, 118.9, 118.8, 113.6, 113.3, 112.5, 110.7, 110.4, 106.1, 84.7, 83.1, 71.0, 56.5, 55.9, 55.7, 54.8, 44.7, 44.2, 44.1, 41.3, 14.8, 14.5, 14.2, and 11.5; HRMS (Q–TOF) *m*/*z*: 365.1730 ((M + Na)^+^, C_21_H_26_NaO_4_ requires 365.1729). 

4-((2*R*,3*R*,4*R*,5*R*)-5-(4-(Benzyloxy)-3-methoxyphenyl)-3,4-dimethyltetrahydrofuran-2-yl)-2-methoxyphenol (**18A**): To a cooled (−78 °C) solution of **17** (42.8 mg, 0.125 mmol) and 2-methoxyphenol, **18**, (78.0 mg, 0.625 mmol, 5 equiv.) in CH_2_Cl_2_ (1.3 mL, 0.1 M), BF_3_∙OEt_2_ (93 μL, 0.75 mmol, 6 equiv.) was added. After being stirred for 30 min at −20 °C, the reaction mixture was quenched with saturated aqueous NaHCO_3_ (5 mL) and diluted with CH_2_Cl_2_ (5 mL). The layers were separated, and the aqueous layer was extracted with CH_2_Cl_2_ (5 mL × 3). The combined organic layers were washed with brine (10 mL × 1), dried over anhydrous Na_2_SO_4_, filtered, and concentrated in vacuo. The residue was purified by column chromatography (SiO_2_, 17% EtOAc/hexane) to provide **18A** (44.0 mg, 81%) as a white oil: [α]_D_^25^ +52.1 (*c* 0.23, CHCl_3_); ^1^H-NMR (500 MHz, CDCl_3_) δ 7.43 (d, *J* = 7.4 Hz, 2H); 7.33–7.37 (m, 2H); 7.27–7.31 (m, 1H); 6.98 (s, 1H); 6.94 (d, *J* = 1.6 Hz, 1H); 6.89 (d, *J* = 8.1 Hz, 1H); 6.86 (d, *J* = 1.7 Hz, 1H); 6.85 (s, 2H); 5.58 (br s, 1H); 5.15 (s, 2H); 4.63 (dd, *J* = 9.1, 1.9 Hz, 2H); 3.92 (s, 3H); 3.91 (s, 3H); 1.72–1.83 (m, 2H); 1.04 (d, *J* = 5.1 Hz, 6H); ^13^C-NMR (125 MHz, CDCl_3_) δ 149.7, 147.6, 146.6, 145.1, 137.2, 135.6, 134.3, 128.5, 127.7, 127.3, 119.3, 118.6, 114.0, 113.8, 109.8, 108.5, 88.4, 88.2, 71.1, 56.0, 55.9, 51.0, 50.9, 13.9, and 13.8; HRMS (EI) *m*/*z*: 434.2093 ((M)^+^, C_27_H_30_O_5_ requires 434.2093).

(+)-Fragransin A_2_ (**2**): To a solution of **18A** (11 mg, 0.025 mmol) in MeOH (1.3 mL, 0.02 M), Pd/C (10%, 55 mg) was added, and the reaction mixture was hydrogenated at 1 atm. After being stirred for 30 min at 25 °C, the reaction mixture was filtered through a pad of celite and concentrated in vacuo. The residue was purified by column chromatography (SiO_2_, 50% EtOAc/hexane) to provide natural (+)-fragransin A_2_ (**2**, 8.2 mg, 95%) as a colorless oil for which the spectral data were identical to those of the known synthetic **2 [15,18]**: [α]_D_^25^ +75.0 (*c* 0.12, CHCl_3_) vs. [α]_D_^25^ +86.9 (*c* 0.88, CHCl_3_) [15]; ^1^H-NMR (500 MHz, CDCl_3_) δ 6.95 (d, *J* = 1.6 Hz, 2H); 6.90 (d, *J* = 8.0 Hz, 2H); 6.87 (dd, *J* = 8.1, 1.7 Hz, 2H); 5.57 (s, 2H); 4.63 (d, *J* = 9.2 Hz, 2H); 3.92 (s, 6H); 1.72–1.80 (m, 2H); 1.04 (d, *J* = 6.0 Hz, 6H); ^13^C-NMR (125 MHz, CDCl_3_) δ 146.6, 145.1, 134.3, 119.4, 113.9, 108.4, 88.3, 55.9, 51.0, and 13.8; HRMS (Q–TOF) *m*/*z*: 367.1523 ((M + Na)^+^, C_20_H_24_NaO_5_ requires 367.1521). 

(+)-Galbelgin (**3**): To a cooled (0 °C) solution of **2** (8.0 mg, 0.023 mmol) in DMF/THF (2:1, a total of 0.6 mL, 0.038 M), NaH (3.7 mg, 60% dispersion in mineral oil, 0.092 mmol, 4 equiv.) was added. After being stirred for 10 min at 0 °C, the reaction mixture was added to MeI (58 μL, 1.0 M in THF, 0.058 μmol, 2.5 equiv.) and stirred for 30 min at 25 °C before it was quenched with the addition of saturated aqueous NH_4_Cl (5 mL) and diluted with EtOAc (5 mL). The layers were separated, and the aqueous layer was extracted with EtOAc (5 mL × 3). The combined organic layers were washed with brine (5 mL × 1), dried over anhydrous Na_2_SO_4_, filtered, and concentrated in vacuo. The residue was purified by column chromatography (SiO_2_, 25% EtOAc/hexane) to provide natural (+)-galbelgin (**3**, 8.5 mg, 99%) as a colorless oil for which the spectral data were identical to those of the known synthetic **3 [10,18,19,20]**: [α]_D_^25^ +83.3 (*c* 0.06, CHCl_3_) vs. [α]_D_ +80.0 (*c* 0.5, CHCl_3_) [20]; ^1^H-NMR (500 MHz, CDCl_3_) δ 6.96 (d, *J* = 1.9 Hz, 2H); 6.92 (dd, *J* = 8.2, 1.9 Hz, 2H); 6.84 (d, *J* = 8.2 Hz, 2H); 4.66 (d, *J* = 9.2 Hz, 2H); 3.91 (s, 6H); 3.88 (s, 6H); 1.75–1.84 (m, 2H); 1.05 (d, *J* = 6.0 Hz, 6H); ^13^C-NMR (125 MHz, CDCl_3_) δ 149.0, 148.5, 134.9, 118.6, 110.8, 109.1, 88.3, 55.89, 55.87, 51.0, and 13.8; HRMS (Q–TOF) *m*/*z*: 373.2016 ((M + H)^+^, C_22_H_29_O_5_ requires 373.2015). 

### 3.4. Synthesis of (+)-Talaumidin (**4**) and (+)-Galbacin (**5**)

(2*S*,3*S*)-3-(Benzo[*d*][1,3]dioxol-5-yl)-1-((*S*)-4-benzyl-2-thioxothiazolidin-3-yl)-3-hydroxy-2-methylpropan-1-one (**7**). To a cooled (0 °C) solution of (*S*)-1-(4-benzyl-2-thioxothiazolidin-3-yl)propan-1-one, **10**, (590 mg, 2.22 mmol) in CH_2_Cl_2_ (11 mL, 0.2 M), titanium(IV) chloride (2.4 mL, 1.0 M in CH_2_Cl_2_, 2.4 mmol, 1.1 equiv.) was added. After being stirred for 15 min at the same temperature, *i*-Pr_2_NEt (0.47 mL, 2.7 mmol, 1.2 equiv.) was added dropwise, and the reaction mixture was stirred for 40 min at 0 °C. NMP (0.43 mL, 4.4 mmol, 2 equiv.) was added, and the reaction mixture was stirred for an additional 10 min. Piperonal (667 mg, 4.44 mmol, 2 equiv.) in CH_2_Cl_2_ (5 mL) was added to the enolate. After being stirred for 30 min, the reaction mixture was quenched with saturated aqueous NH_4_Cl (10 mL) and diluted with CH_2_Cl_2_ (10 mL). The layers were separated, and the aqueous layer was extracted with CH_2_Cl_2_ (10 mL × 3). The combined organic layers were washed with brine (20 mL × 1), dried over anhydrous Na_2_SO_4_, filtered, and concentrated in vacuo. The residue was purified by column chromatography (SiO_2_, 25% EtOAc/hexane) to provide **7** (775 mg, 84%) as a yellow oil: [α]_D_^25^ +156 (*c* 1.16, CHCl_3_); ^1^H-NMR (500 MHz, CDCl_3_) δ 7.31–7.35 (m, 2H); 7.26–7.29 (m, 1H); 7.22–7.26 (m, 2H); 6.87 (d, *J* = 1.6 Hz, 1H); 6.78 (dd, *J* = 8.0, 1.5 Hz, 1H); 6.73 (d, *J* = 8.0 Hz, 1H); 5.93 (ABq, 2H, *J* = 1.4 Hz, Δν = 1.8 Hz); 4.89 (ddd, *J* = 10.6, 6.8, 4.0 Hz, 1H); 4.81 (d, *J* = 6.2 Hz, 1H); 4.73 (p, *J* = 6.6 Hz, 1H); 3.17 (dd, *J* = 13.2, 3.8 Hz, 1H); 2.93–2.98 (m, 3H); 2.76 (d, *J* = 11.4 Hz, 1H); 1.30 (d, *J* = 6.7 Hz, 3H); ^13^C-NMR (125 MHz, CDCl_3_) δ 201.1, 177.0, 147.5, 146.9, 136.2, 135.6, 129.3, 128.8, 127.1, 119.5, 107.9, 106.7, 100.9, 75.4, 68.8, 46.3, 36.5, 32.2, and 12.1; HRMS (Q–TOF) *m*/*z*: 416.0997 ((M + H)^+^, C_21_H_22_NO_4_S_2_ requires 416.0990). 

(4*R*,5*S*)-5-(Benzo[*d*][1,3]dioxol-5-yl)-4-methyldihydrofuran-2(3H)-one (**9**): To a cooled (−78 °C) solution of *i*-Pr_2_NH (0.58 mL, 4.1 mmol, 4.4 equiv.) in THF (2.3 mL, 0.4 M), *n*-BuLi (2.3 mL, 1.6 M in hexane, 3.8 mmol, 4 equiv.) was added, and the reaction mixture was stirred for 15 min at 0 °C before it was cooled to −78 °C. CH_2_Br_2_ (4.1 mL, 1.0 M in THF, 4.1 mmol, 4.4 equiv.) and **7** (390 mg, 0.939 mmol) in THF (4.0 mL) were added dropwise to the above LDA solution. After being stirred for 10 min at −78 °C, the reaction mixture was treated with LiHMDS (1.9 mL, 1.0 M in THF, 1.9 mmol, 2 equiv.). After being stirred for 5 min at −78 °C, the reaction mixture was stirred for 90 s at −20 °C before it was cooled to −78 °C. *s*-BuLi (1.3 mL, 1.4 M in cyclohexane, 1.9 mmol, 2 equiv.) was added dropwise, and the reaction mixture was stirred for 90 s at −20 °C and cooled to −78 °C, followed by the dropwise addition of *n*-BuLi (2.3 mL, 1.6 M in hexane, 3.8 mmol, 4 equiv.). After being stirred for 5 min, the reaction mixture was stirred for 90 s at –20 °C, warmed to 25 °C in a water bath, and stirred for 30 min before it was added to a solution of HCl (86 μL, 2.8 mmol, 3 equiv.) in THF (31 mL, 0.03 M) at −20 °C. After being stirred for 5 min at −20 °C, the reaction mixture was quenched with the addition of saturated aqueous NaHCO_3_ (10 mL), and diluted with EtOAc (10 mL). The layers were separated, and the aqueous layer was extracted with EtOAc (10 mL × 3). The combined organic layers were washed with brine (15 mL × 1), dried over anhydrous Na_2_SO_4_, filtered, and concentrated in vacuo. The residue was purified by column chromatography (SiO_2_, 20% EtOAc/hexane) to provide **9** (194 mg, 94%) as a colorless oil: [α]_D_^25^ −12.5 (*c* 0.40, CHCl_3_); ^1^H-NMR (500 MHz, CDCl_3_) δ 6.76 (d, *J* = 8.0 Hz, 1H); 6.67 (d, *J* = 1.4 Hz, 1H); 6.65 (dd, *J* = 8.0, 1.6 Hz, 1H); 5.92 (s, 2H); 5.46 (d, *J* = 5.9 Hz, 1H); 2.74–2.82 (m, 2H); 2.25–2.32 (m, 1H); 0.67 (d, *J* = 7.0 Hz, 3H); ^13^C-NMR (125 MHz, CDCl_3_) δ 176.5, 147.7, 147.1, 129.8, 118.7, 108.0, 105.9, 101.0, 83.8, 36.8, 34.8, and 14.9; HRMS (Q–TOF) *m*/*z*: 221.0821 ((M + H)^+^, C_12_H_13_O_4_ requires 221.0814). 

(3*R*,4*R*,5*S*)-5-(Benzo[*d*][1,3]dioxol-5-yl)-3,4-dimethyldihydrofuran-2(3H)-one (**9A**): To a cooled (−78 °C) solution of **9** (210 mg, 0.954 mmol) in THF (9.5 mL, 0.1 M), LiHMDS (1.9 mL, 1.0 M in THF, 1.9 mmol, 2 equiv.) was added. After being stirred for 1 h, the reaction mixture was added to MeI (71.2 μL, 1.15 mmol, 1.2 equiv.), and the reaction mixture was stirred for 10 min at −78 °C before it was quenched with the addition of saturated aqueous NH_4_Cl (15 mL) and diluted with EtOAc (15 mL). The layers were separated, and the aqueous layer was extracted with EtOAc (15 mL × 3). The combined organic layers were washed with brine (30 mL × 1), dried over anhydrous Na_2_SO_4_, filtered, and concentrated in vacuo. The residue was purified by column chromatography (SiO_2_, 25% EtOAc/hexane) to provide **9A** (206 mg, 92%) as a colorless oil: [α]_D_^25^ +30.1 (*c* 1.72, CHCl_3_); ^1^H-NMR (500 MHz, CDCl_3_) δ 6.73 (d, *J* = 7.8 Hz, 1H); 6.55−6.58 (m, 2H); 5.90−5.92 (m, 2H); 5.40 (d, *J* = 7.8 Hz, 1H); 2.41 (dp, *J* = 10.3, 7.0 Hz, 1H); 2.28 (dq, *J* = 10.3, 7.0 Hz, 1H); 1.21 (d, *J* = 7.1 Hz, 3H); 0.71 (d, *J* = 7.0 Hz, 3H); ^13^C-NMR (125 MHz, CDCl_3_) δ 179.5, 147.7, 147.2, 129.8, 119.1, 107.9, 106.1, 101.1, 82.2, 42.1, 39.6, 14.3, and 13.4; HRMS (Q–TOF) *m*/*z*: 235.0993 ((M + H)^+^, C_13_H_15_O_4_ requires 235.0970). 

5-((2*S*,3*R*,4*R*)-5-Methoxy-3,4-dimethyltetrahydrofuran-2-yl)benzo[*d*][1,3]dioxole (**19**): To a cooled (−78 °C) solution of **9A** (180 mg, 0.770 mmol) in toluene (19 mL, 0.04 M), DIBALH (0.85 mL, 1.0 M in toluene, 0.85 mmol, 1.1 equiv.) was added. After being stirred for 10 min at −78 °C, the reaction mixture was diluted with MeOH (38 mL, 0.02 M) before it was added to HC(OMe)_3_ (0.84 mL, 7.7 mmol, 10 equiv.) and *p*-TsOH (66.3 mg, 0.385 mmol, 50 mol%). After being stirred for 12 h at 25 °C, the reaction mixture was quenched with saturated aqueous NaHCO_3_ (30 mL) and diluted with EtOAc (30 mL). The layers were separated, and the aqueous layer was extracted with EtOAc (30 mL × 3). The combined organic layers were washed with brine (45 mL × 1), dried over anhydrous Na_2_SO_4_, filtered, and concentrated in vacuo. The residue was purified by column chromatography (SiO_2_, 10% EtOAc/hexane) to provide a 1.5:1 anomeric mixture of cyclic methyl acetal, **19**, (190 mg, 99%) as a colorless oil: For the major diastereomer: ^1^H-NMR (500 MHz, CDCl_3_) δ 6.75 (s, 1H); 6.68 (d, *J* = 1.6 Hz, 1H); 6.64 (dd, *J* = 8.0, 1.2 Hz, 1H); 5.92 (s, 2H); 5.13 (d, *J* = 8.9 Hz, 1H); 5.01 (d, *J* = 4.7 Hz, 1H); 3.41 (s, 3H); 2.22–2.31 (m, 1H); 1.78–1.88 (m, 1H); 1.01 (d, *J* = 6.8 Hz, 3H); 0.56 (d, *J* = 7.0 Hz, 3H); for the minor diastereomer: ^1^H-NMR (500 MHz, CDCl_3_) δ 6.91 (s, 1H); 6.75 (s, 1H); 6.74 (s, 1H); 5.93 (s, 2H); 5.10 (d, *J* = 7.6 Hz, 1H); 4.71 (d, *J* = 5.1 Hz, 1H); 3.53 (s, 3H); 2.11 (dp, *J* = 9.0, 7.1 Hz, 1H); 1.78–1.88 (m, 1H); 1.07 (d, *J* = 6.9 Hz, 3H); 0.63 (d, *J* = 7.0 Hz, 3H); **for 19:** ^13^C-NMR (125 MHz, CDCl_3_) δ 147.4, 147.3, 146.5, 146.4, 134.73, 134.66, 119.9, 112.4, 107.7, 107.2, 106.2, 100.81, 100.76, 84.5, 83.2, 56.6, 54.8, 44.7, 44.1, 43.9, 41.3, 14.9, 14.4, 14.3, and 11.5.

(+)-Talaumidin (**4**): To a cooled (−78 °C) solution of **19** (56.5 mg, 0.226 mmol) and 2-methoxyphenol, **20**, (140 mg, 1.13 mmol, 5 equiv.) in CH_2_Cl_2_ (2.3 mL, 0.1 M), BF_3_∙OEt_2_ (0.17 mL, 1.36 mmol, 6 equiv.) was added. After being stirred for 30 min at −20 °C, the reaction mixture was quenched with saturated aqueous NaHCO_3_ (10 mL) and diluted with CH_2_Cl_2_ (10 mL). The layers were separated, and the aqueous layer was extracted with CH_2_Cl_2_ (10 mL × 3). The combined organic layers were washed with brine (15 mL × 1), dried over anhydrous Na_2_SO_4_, filtered, and concentrated in vacuo. The residue was purified by column chromatography (SiO_2_, 17% EtOAc/hexane) to provide natural (+)-talaumidin (**4**, 67.9 mg, 88%) as a colorless oil for which the spectral data were identical to those of the known synthetic **4 [18,39,41]**: [α]_D_^25^ +75.9 (*c* 0.16, CHCl_3_) vs. [α]_D_^20^ +88.3 (*c* 2.1, CHCl_3_) [39]; ^1^H-NMR (500 MHz, CDCl_3_) δ 6.93 (dd, *J* = 4.5, 1.6 Hz, 2H); 6.89 (d, *J* = 8.1 Hz, 1H); 6.82–6.87 (m, 2H); 6.78 (d, *J* = 7.9 Hz, 1H); 5.94–5.95 (m, 2H); 5.61 (s, 1H); 4.62 (d, *J* = 9.2 Hz, 2H); 3.91 (s, 3H); 1.71–1.82 (m, 2H); 1.04 (d, *J* = 6.2 Hz, 3H); 1.02 (d, *J* = 6.2 Hz, 3H); ^13^C-NMR (125 MHz, CDCl_3_) δ 147.8, 147.0, 146.6, 145.1, 136.6, 134.1, 119.7, 119.4, 114.0, 108.6, 108.0, 106.6, 100.9, 88.4, 88.2, 56.0, 51.2, 50.9, 13.84, and 13.81; HRMS (EI) *m*/*z*: 342.1465 ((M)^+^, C_20_H_22_O_5_ requires 342.1467). 

(+)-Galbacin (**5**): To a cooled (−78 °C) solution of **19A** (39.6 mg, 0.158 mmol) and 1,2-methylenedioxybenzene, **21**, (97 mg, 0.79 mmol, 5 equiv.) in CH_2_Cl_2_ (2 mL, 0.08 M), BF_3_∙OEt_2_ (0.12 mL, 0.95 mmol, 6 equiv.) was added. After being stirred for 30 min at −20 °C, the reaction mixture was quenched with saturated aqueous NaHCO_3_ (5 mL) and diluted with CH_2_Cl_2_ (5 mL). The layers were separated, and the aqueous layer was extracted with CH_2_Cl_2_ (5 mL × 3). The combined organic layers were washed with brine (10 mL × 1), dried over anhydrous Na_2_SO_4_, filtered, and concentrated in vacuo. The residue was purified by column chromatography (SiO_2_, 50% CH_2_Cl_2_/hexane) to provide natural (+)-galbacin (**5**, 47.3 mg, 88%) as a colorless oil for which the spectral data were identical to those of the known synthetic **5 [15,20,42]**: [α]_D_^25^ +121 (*c* 0.22, CHCl_3_) vs. [α]_D_^28^ +110 (*c* 1.0, CHCl_3_) [20]; ^1^H-NMR (500 MHz, CDCl_3_) δ 6.92 (d, *J* = 1.5 Hz, 2H); 6.83 (dd, *J* = 8.0, 1.5 Hz, 2H); 6.77 (d, *J* = 7.9 Hz, 2H); 5.94 (s, 4H); 4.60 (d, *J* = 9.2 Hz, 2H); 1.70–1.80 (m, 2H); 1.02 (d, *J* = 6.0 Hz, 6H); ^13^C-NMR (125 MHz, CDCl_3_) δ 147.7, 146.9, 136.3, 119.7, 107.9, 106.5, 100.9, 88.2, 51.0, and 13.7; HRMS (EI) *m*/*z*: 340.1310 ((M)^+^, C_20_H_20_O_5_ requires 340.1311).

## 4. Conclusions

In summary, we have detailed the divergent syntheses of (-)-chicanine, (+)-fragransin A_2_, (+)-galbelgin, (+)-talaumidin, and (+)-galbacin via a significantly improved and highly effective early one-carbon homologative *γ*-butyrolactonization for the construction of the core *γ*-butyrolactone scaffold bearing the required two *β*,*γ*-vicinal stereogenic centers. The synthesis of (-)-chicanine from 2,3-*cis*-3,4-*cis*-4,5-*trans*-tetrahydrofuran was carried out according to Ley’s protocol, using an organozinc addition to the 2-benzenefulfonyl cyclic ether method. The 2,3-*trans*-3,4-*trans*-4,5-*trans*-tetrahydrofurans present in (+)-fragransin A_2_, (+)-galbelgin, (+)-talaumidin, and (+)-galbacin were formed through the BF_3_∙OEt_2_-promoted epimerization of the cyclic methyl acetal. It is noteworthy that all the syntheses presented in the current report are among the shortest syntheses and have the highest overall yields reported to date. We believe that a range of synthetic applications and the further improvement of this strategy will be advantageous to other researchers in their endeavors to synthesize structurally and pharmacologically important natural compounds containing *γ*-butyrolactones or tetrahydrofurans.

## Data Availability

The following data are available online: copies of the ^1^H- and ^13^C-NMR spectra of **1**–**9**, **11**–**13**, **15**–**17**, and **19**.

## Supplementary Materials

The following supporting information can be downloaded at: https://www.mdpi.com/article/10.3390/molecules29030701/s1, Figure S1. Chromatographs of racemic and synthetic **(–)-8**; Figure S2. Chromatographs of racemic and synthetic **(–)-9**; Table S1. Optimization of the One-Carbon Homologative γ-Butyrolactonization; Scheme S1. Alternative synthetic method for **18A**; Scheme S2. Alternative synthetic methods for (+)-talaumidin **(4)** and (+)-galbacin **(5)**; Spectral data of all compounds synthesized **1–9**, **9A**, **11–13**, **15–17**,**18A**, **19** and **19A** (^1^H and ^13^C NMR).
