# Supplementary material for: Divergent Syntheses of (-)-Chicanine, (+)-Fragransin A2, (+)-Galbelgin, (+)-Talaumidin, and (+)-Galbacin via One-Pot Homologative γ-Butyrolactonization
Source: Molecules. 2024 Feb 2;29(3):701. doi: 10.3390/molecules29030701 (PMC10856021; doi:10.3390/molecules29030701)
Supplement: Supplementary file 1 [file molecules-29-00701-s001.zip › molecules-2835839-supplementary.pdf]

Supplementary Material

Divergent Syntheses of (–)-Chicanine, (+)-Fragransin A_2, (+)-Galbelgin, (+)-Talaumidin, and (+)-Galbacin via a One-Pot Homologative γ-Butyrolactonization

Hosam Choi †, Jongyeol Han †, Joohee Choi and Kiyoun Lee *

Department of Chemistry, The Catholic University of Korea, Bucheon 14662, Korea

† These authors contributed equally to this work.

* Correspondence: kiyoun@catholic.ac.kr; Tel.: +82-2-2164-5528; Fax: +82-2-2164-4764

Table of Contents

1. **Figure S1.** Chromatographs of racemic and synthetic **(–)-8** S2

2. **Figure S2.** Chromatographs of racemic and synthetic **(–)-9** S3

3. **Table S1.** Optimization of the One-Carbon Homologative γ-Butyrolactonization S4

4. Alternative synthetic methods for **18A**, (+)-*talaumidin* **(4)** and (+)-*galbacin* **(5)** S5–7

    Scheme 1. Alternative synthetic method for **18A** S5

    Scheme 2. Alternative synthetic methods for (+)-*talaumidin* **(4)** and (+)-*galbacin* **(5)** S6

5. ^{1}H and ^{13}C NMR spectra of **1–9**, **9A**, **11–13**, **15–17**,**18A**, **19**, **19A** S8–75

6. Reference S76

1. Chromatographs of racemic and synthetic (–)-8

(a) racemic

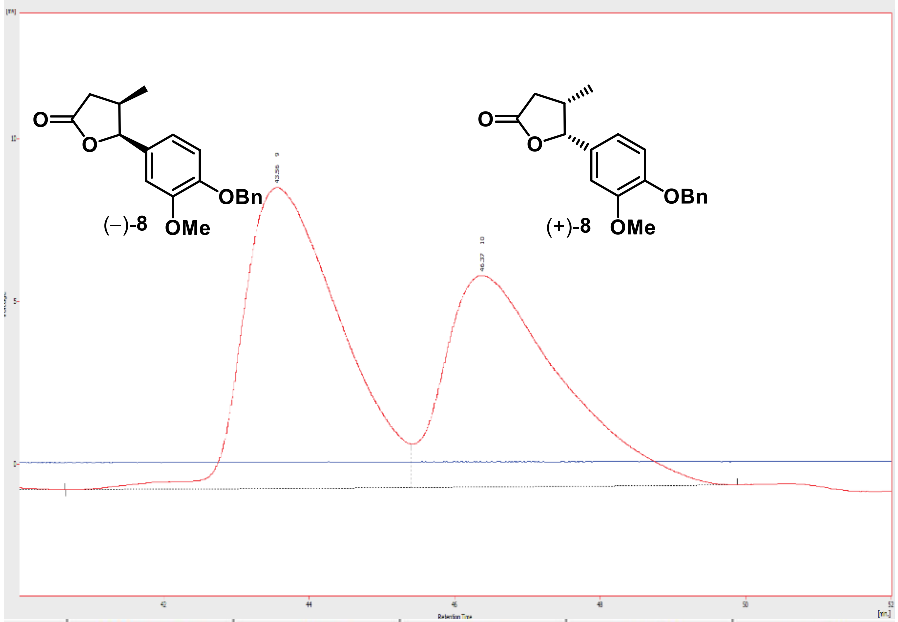

	Reten. Time [min]	Area [mV.s]	Area [%]	Height [mV]	Height [%]
1	43.557	859.384	54.3	9.236	58.7
2	46.373	723.580	45.7	6.487	41.3
	Total	1582.964	100.0	15.723	100.0

(b) synthetic (–)-8

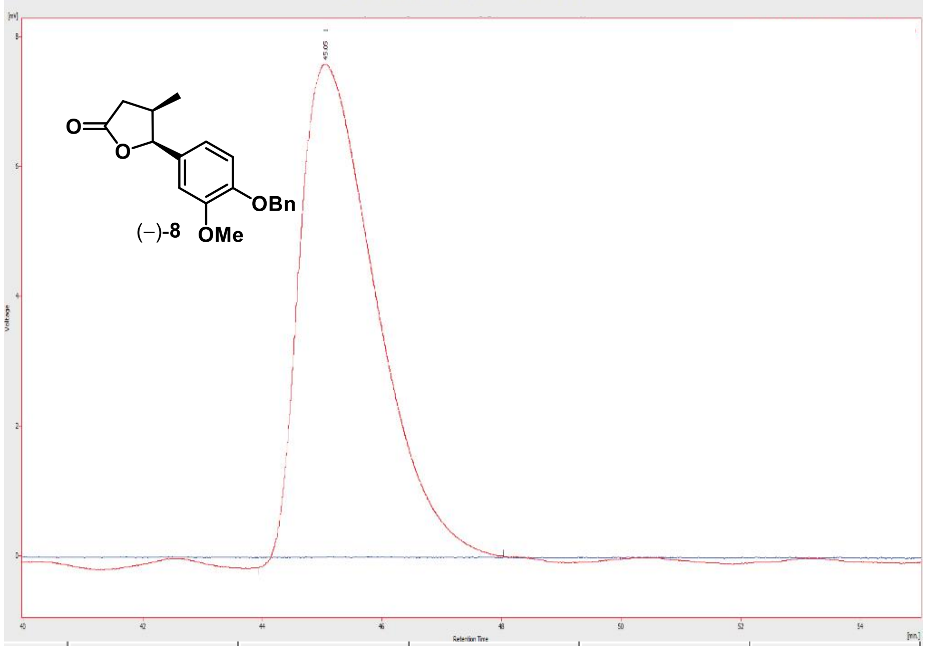

	Reten. Time [min]	Area [mV.s]	Height [mV]	Area [%]	Height [%]
1	45.048	686.699	7.712	100.0	100.0
	Total	686.699	7.712	100.0	100.0

Figure S1. Chiral HPLC (Analytical DaiCel ChiralCel OD column (4.6 x 250 mm), flow rate: 1.0 mL/min, isocratic 10% *i*-PrOH–Hexane). For (–)-**8** (t_R: 43.6 min, $[\alpha]_D^{25}$ –28.0 (c 1.28, $CHCl_3$)) and *ent*-(+)-**8** (t_R: 46.4 min).

2. Chromatographs of racemic and synthetic (–)-9

(a) racemic

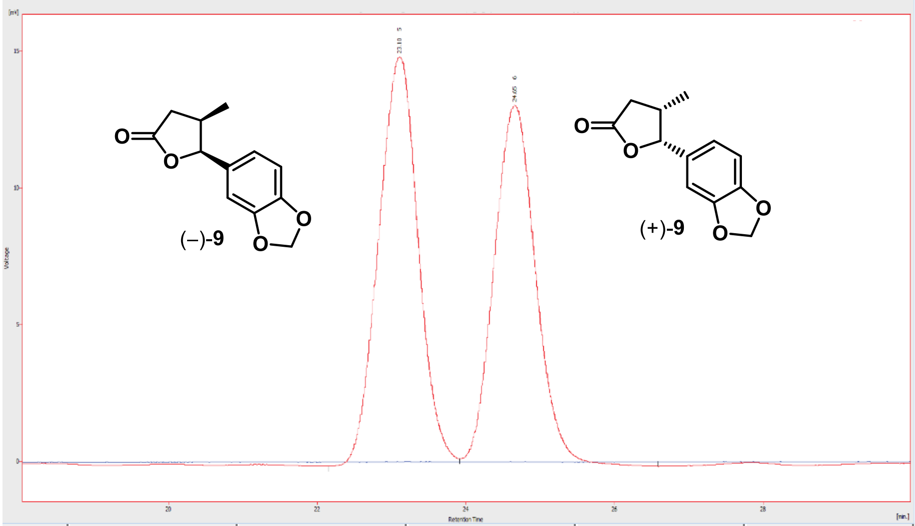

	Reten. Time [min]	Area [mV.s]	Height [mV]	Area [%]	Height [%]
1	23.098	557.257	14.952	51.8	53.1
2	24.648	517.894	13.180	48.2	46.9
	Total	1075.151	28.132	100.0	100.0

(b) synthetic (–)-9

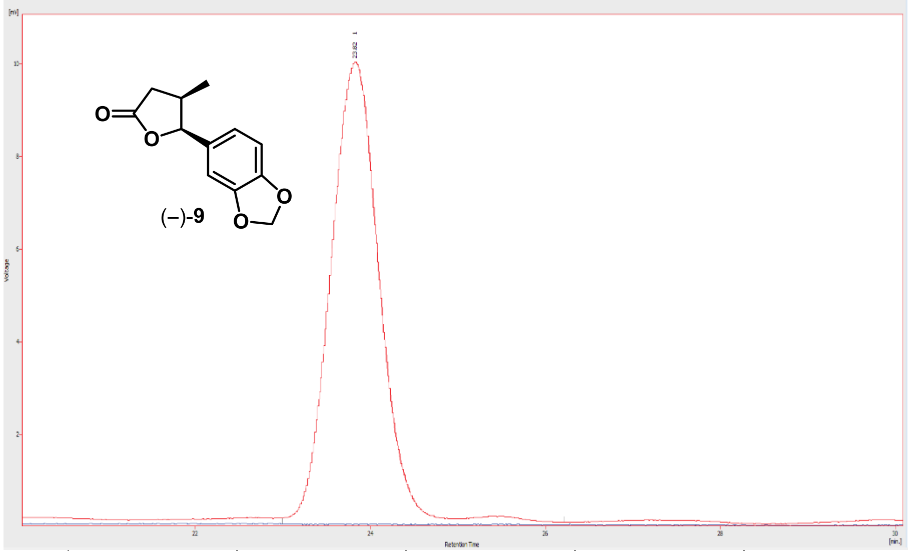

	Reten. Time [min]	Area [mV.s]	Height [mV]	Area [%]	Height [%]
1	23.815	377.850	9.868	100.0	100.0
	Total	377.850	9.868	100.0	100.0

Figure S2. Chiral HPLC (Analytical DaiCel ChiralCel AD column (4.6 x 250 mm), flow rate: 1.0 mL/min, isocratic 5% *i*-PrOH–Hexane). For (–)-**9** (t_R: 23.1 min, $[\alpha]_D^{25}$ –12.5 (c 0.4, $CHCl_3$)) and *ent*-(+)-**9** (t_R: 24.6 min).

3. Optimization of the One-Carbon Homologative γ-Butyrolactonization

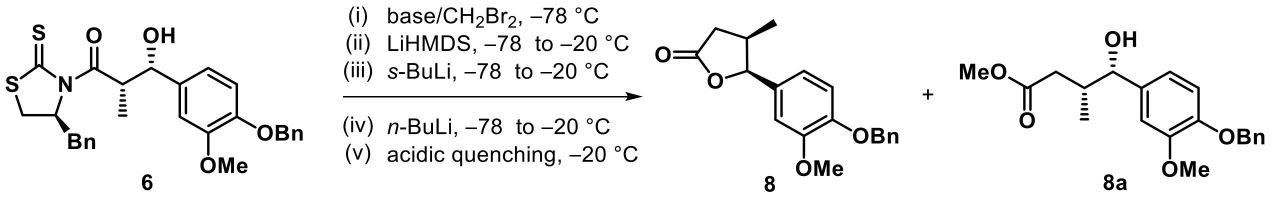

entry	conditions						yield(%)[a]	
	(i) base/CH_2Br_2	(ii) LiHMDS (equiv)	(iii) *s*-BuLi (equiv)	(iv) *n*-BuLi (equiv)	(ii–iv) time (s)	(v) acidic quenching	**8**	**8a**
1	LiTMP	4	-	4	90	acidic MeOH	trace	
2	LiTMP	4	1	4	30	acidic MeOH	N.D[b]	
3	LiTMP	4	1	4	90	acidic MeOH	35	17
4	LiTMP	4	2	4	90	acidic MeOH	42	20
5	LiTMP	4	4	4	90	acidic MeOH	60	35
**6**	**LDA**	**4**	**4**	**4**	**90**	**HCl** (3 equiv)/**THF**	**91**	-
7	LDA	2	4	4	90	HCl (3 equiv)/THF	39	-
8	LiTMP	4	6	4	90	acidic MeOH	N.D[b]	-

(i) base/CH_2Br_2, -78 °C (ii) LiHMDS, -78 to -20 °C, (iii) *s*-BuLi, -78 to -20 °C
(iv) *n*-BuLi, -78 to 25 °C, (v) acidic workup, -20 °C. [a] Isolated yield, [b] No product observed

Table S1. Optimization of the One-Carbon Homologative γ-Butyrolactonization

4. Alternative synthetic methods for 18A, (+)-*talaumidin* (4) and (+)-*galbacin* (5)

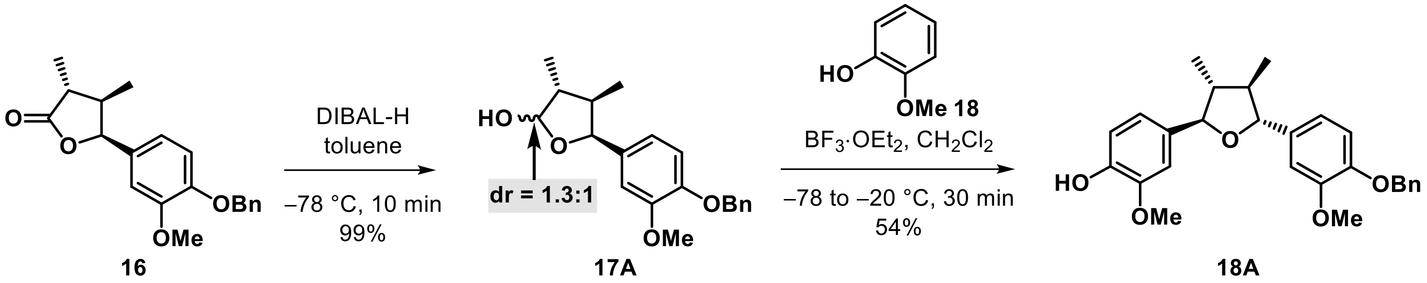

Scheme 1. Alternative synthetic method for **18A**

(3R,4R,5S)-5-(4-(benzyloxy)-3-methoxyphenyl)-3,4-dimethyltetrahydrofuran-2-ol **(17A):** To a cooled (–78 °C) solution of **16** (112 mg, 0.343 mmol) in toluene (3.4 mL, 0.1 M) was added DIBALH (0.38 mL, 1.0 M in toluene, 0.38 mmol, 1.1 equiv). After being stirred for 10 min at –78 °C, the reaction mixture was quenched with MeOH (0.1 mL) followed by aqueous Rochelle's salt solution (10 mL) and diluted with Et_2O (10 mL). The resulting mixture was stirred for 6 h at 25 °C. The layers were separated, and the aqueous layer was extracted with Et_2O (10 mL×3). The combined organic layers were washed with brine (15 mL×1), dried over anhydrous Na_2SO_4, filtered, and concentrated in vacuo. The residue was purified by column chromatography (SiO_2, 50% EtOAc/hexane) to provide an 1.3:1 anomeric mixture of cyclic hemiketal **17A** (111 mg, 99%) as colorless oil: **For major diastereomer:** ^{1}H-NMR (500 MHz, $CDCl_3$) δ 7.42–7.45 (m, 2H), 7.34–7.38 (m, 2H), 7.30 (d, *J* = 7.5 Hz, 1H), 6.82 (d, *J* = 2.8 Hz, 1H), 6.70 (d, *J* = 1.9 Hz, 1H), 6.64 (dd, *J* = 8.3, 1.8 Hz, 1H), 5.57 (d, *J* = 4.7 Hz, 1H), 5.26 (d, *J* = 8.7, 1H), 5.13 (s, 2H), 3.87 (s, 3H), 2.30–2.39 (m, 1H), 1.75–1.87 (m, 1H), 1.03 (d, *J* = 6.8 Hz, 3H), 0.57 (d, *J* = 7.0 Hz, 3H); **For minor diastereomer:** ^{1}H-NMR (500 MHz, $CDCl_3$) δ 7.42–7.45 (m, 2H), 7.34–7.38 (m, 2H), 7.29 (d, *J* = 7.1 Hz, 1H), 7.06 (d, *J* = 1.7 Hz, 1H), 6.84 (d, *J* = 2.8 Hz, 1H), 6.80 (dd, *J* = 8.3, 1.8 Hz, 1H), 5.19 (d, *J* = 5.8 Hz, 1H), 5.14 (s, 2H), 5.09 (d, *J* = 7.8, 1H), 3.88 (s, 3H), 2.10–2.18 (m, 1H), 1.75–1.87 (m, 1H), 1.06 (d, *J* = 6.8 Hz, 3H), 0.63 (d, *J* = 7.0 Hz, 3H); **For 17A:** ^{13}C-NMR (125 MHz, CDCl3) δ 149.1, 147.1, 147.0, 137.2, 137.1, 133.6, 133.5, 128.4, 127.7, 127.6, 127.2, 118.9, 118.7, 113.4, 113.3, 110.8, 110.4, 105.1, 99.5, 84.2, 83.3, 70.9, 55.9, 55.8, 44.9, 44.1, 43.5, 40.9, 14.2, 14.1, 14.0, 11.5; HRMS (Q–TOF) *m*/*z*: 351.1574 [$(M+Na)^+$, $C_{20}H_{24}NaO_4$ requires 351.1572].

4-((2R,3R,4R,5R)-5-(4-(benzyloxy)-3-methoxyphenyl)-3,4-dimethyltetrahydrofuran-2-yl)-2-methoxyphenol **(18A):** To a cooled (–78 °C) solution of **17A** (28 mg, 0.085 mmol) and 2-methoxyphenol **18** (53 mg, 0.43 mmol, 5 equiv) in CH_2Cl_2 (1.1 mL, 0.08 M) was added $BF_3·OEt_2$ (63.0 μL, 0.510 mmol, 6 equiv). After being stirred for 30 min at –20 °C, the reaction mixture was quenched with the saturated aqueous $NaHCO_3$ (5 mL) and diluted with CH_2Cl_2 (5 mL). The layers were separated, and the aqueous layer was extracted with CH_2Cl_2 (5 mL×3). The combined organic layers were washed with brine (10 mL×1), dried over anhydrous Na_2SO_4, filtered, and concentrated in vacuo. The residue was purified by column chromatography (SiO_2, 17% EtOAc/hexane) to provide **18A** (20.0 mg, 54%) as a white oil.

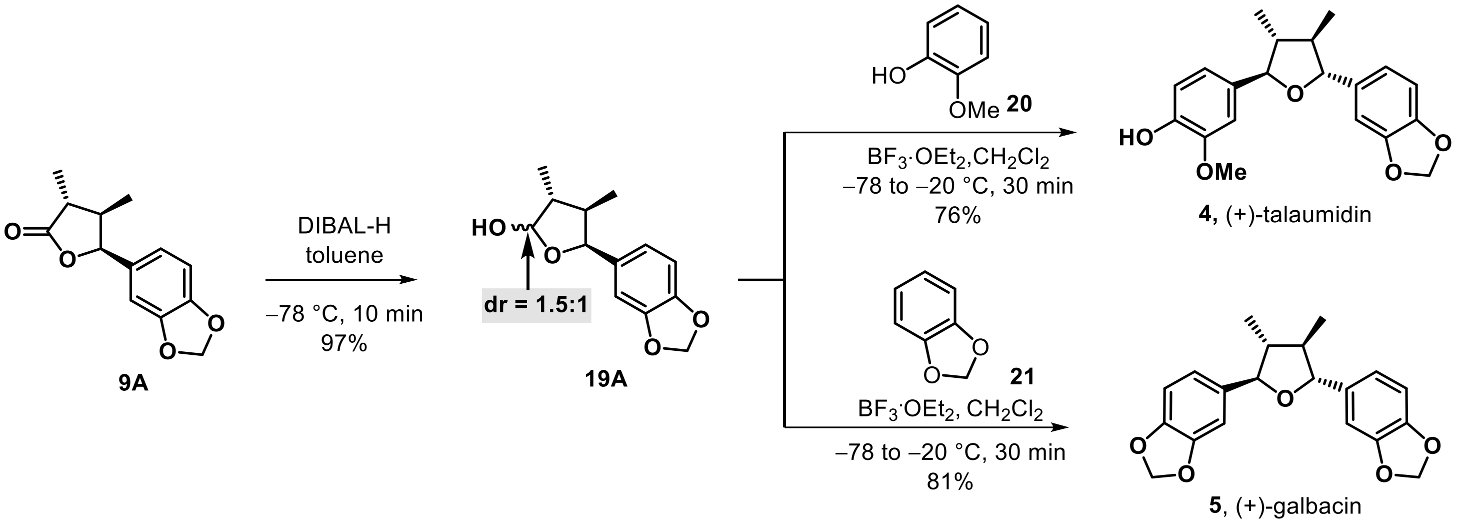

Scheme 2. Alternative synthetic methods for (+)-*talaumidin* **(4)** and (+)-*galbacin* **(5)**

(3R,4R,5S)-5-(benzo[d][1,3]dioxol-5-yl)-3,4-dimethyltetrahydrofuran-2-ol **(19A):** To a cooled (–78 °C) solution of **9A** (200 mg, 0.854 mmol) in toluene (8.5 mL, 0.1 M) was added DIBALH (0.94 mL, 1.0 M in toluene, 0.94 mmol, 1.1 equiv). After being stirred for 10 min at –78 °C, the reaction mixture was quenched with MeOH (0.1 mL) followed by aqueous Rochelle's salt solution (20 mL) and diluted with Et_2O (20 mL). The resulting mixture was stirred for 6 h at 25 °C. The layers were separated, and the aqueous layer was extracted with Et_2O (20 mL×3). The combined organic layers were washed with brine (40 mL×1), dried over anhydrous Na_2SO_4, filtered, and concentrated in vacuo. The residue was purified by column chromatography (SiO_2, 50% EtOAc/hexane) to provide an 1.5:1 anomeric mixture of cyclic hemiketal **19A** (196 mg, 97%) as colorless oil: **For major diastereomer:** ^{1}H-NMR (500 MHz, $CDCl_3$) δ 6.76 (d, J = 6.4 Hz, 1H), 6.66 (d, J = 1.4 Hz, 1H), 6.60–6.63 (m, 1H), 5.93 (s, 2H), 5.56 (d, J = 4.7 Hz, 1H), 5.24 (d, J = 8.7 Hz, 1H), 3.49 (br s, 1H), 2.29–2.38 (m, 1H), 1.73–1.86 (m, 1H), 1.02 (d, J = 6.8 Hz, 3H), 0.58 (d, J = 7.0 Hz, 3H); **For minor diastereomer:** ^{1}H-NMR (500 MHz, $CDCl_3$) δ 6.95 (d, J = 1.3 Hz, 1H), 6.80 (dd, J = 8.0, 1.5 Hz, 1H), 6.75 (d, J = 7.9 Hz, 1H), 5.94 (s, 2H), 5.17 (d, J = 5.7 Hz, 1H), 5.07 (d, J = 7.8 Hz, 1H), 4.25 (br s, 1H), 2.08–2.17 (m, 1H), 1.73–1.86 (m, 1H), 1.05 (d, J = 6.8 Hz, 3H), 0.63 (d, J = 7.0 Hz, 3H); **For 19A:** ^{13}C-NMR (125 MHz, $CDCl_3$) δ 147.38, 147.37, 146.6, 146.50, 134.5, 134.4, 120.0, 119.8, 107.7, 107.63, 107.61, 107.2, 105.2, 100.9, 100.8, 99.7, 84.2, 83.6, 45.2, 44.1, 43.6, 40.9, 14.4, 14.2, 14.1, 11.5; HRMS (Q–TOF) *m/z*: 259.0953 $[(M+Na)^+$, $C_{13}H_{16}NaO_4$ requires 259.0946].

(+)-*talaumidin* **(4):** To a cooled (–78 °C) solution of **19A** (13 mg, 0.055 mmol) and 2-methoxyphenol **20** (34.1 mg, 0.275 mmol, 5 equiv) in CH_2Cl_2 (0.7 mL, 0.08 M) was added $BF_3{\cdot}OEt_2$ (41 μL, 0.33 mmol, 6 equiv). After being stirred for 30 min at –20 °C, the reaction mixture was quenched with the saturated aqueous $NaHCO_3$ (5 mL)

and diluted with CH_2Cl_2 (5 mL). The layers were separated, and the aqueous layer was extracted with CH_2Cl_2 (5 mL×3). The combined organic layers were washed with brine (10 mL×1), dried over anhydrous Na_2SO_4, filtered, and concentrated in vacuo. The residue was purified by column chromatography (SiO_2, 17% EtOAc/hexane) to provide natural (+)-talaumidin (**4**, 14.3 mg, 76%) as a colorless oil.

(+)-*galbacin* **(5):** To a cooled (–78 °C) solution of **19A** (14.6 mg, 61.8 μmol) and 1,2-methylenedioxybenzene **21** (37.7 mg, 0.310 mmol, 5 equiv) in CH_2Cl_2 (0.8 mL, 0.08 M) was added $BF_3 \cdot OEt_2$ (46 μL, 0.37 mmol, 6 equiv). After being stirred for 30 min at –20 °C, the reaction mixture was quenched with the saturated aqueous $NaHCO_3$ (5 mL) and diluted with CH_2Cl_2 (5 mL). The layers were separated, and the aqueous layer was extracted with CH_2Cl_2 (5 mL×3). The combined organic layers were washed with brine (10 mL×1), dried over anhydrous Na_2SO_4, filtered, and concentrated in vacuo. The residue was purified by column chromatography (SiO_2, 50% CH_2Cl_2/hexane) to provide natural (+)-galbacin (**5**, 17 mg, 81%) as a colorless oil.

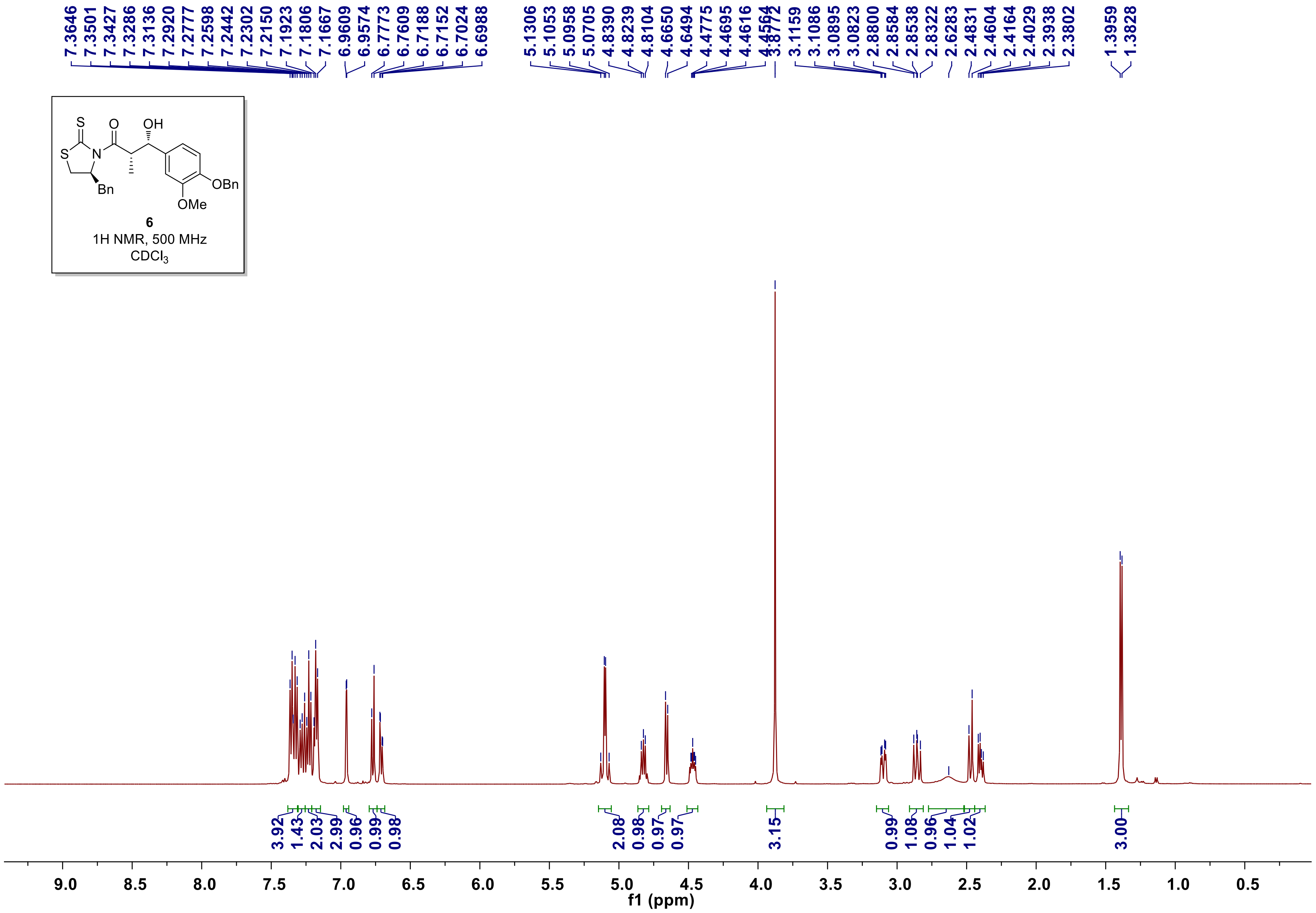

6
1H NMR, 500 MHz
CDCl3

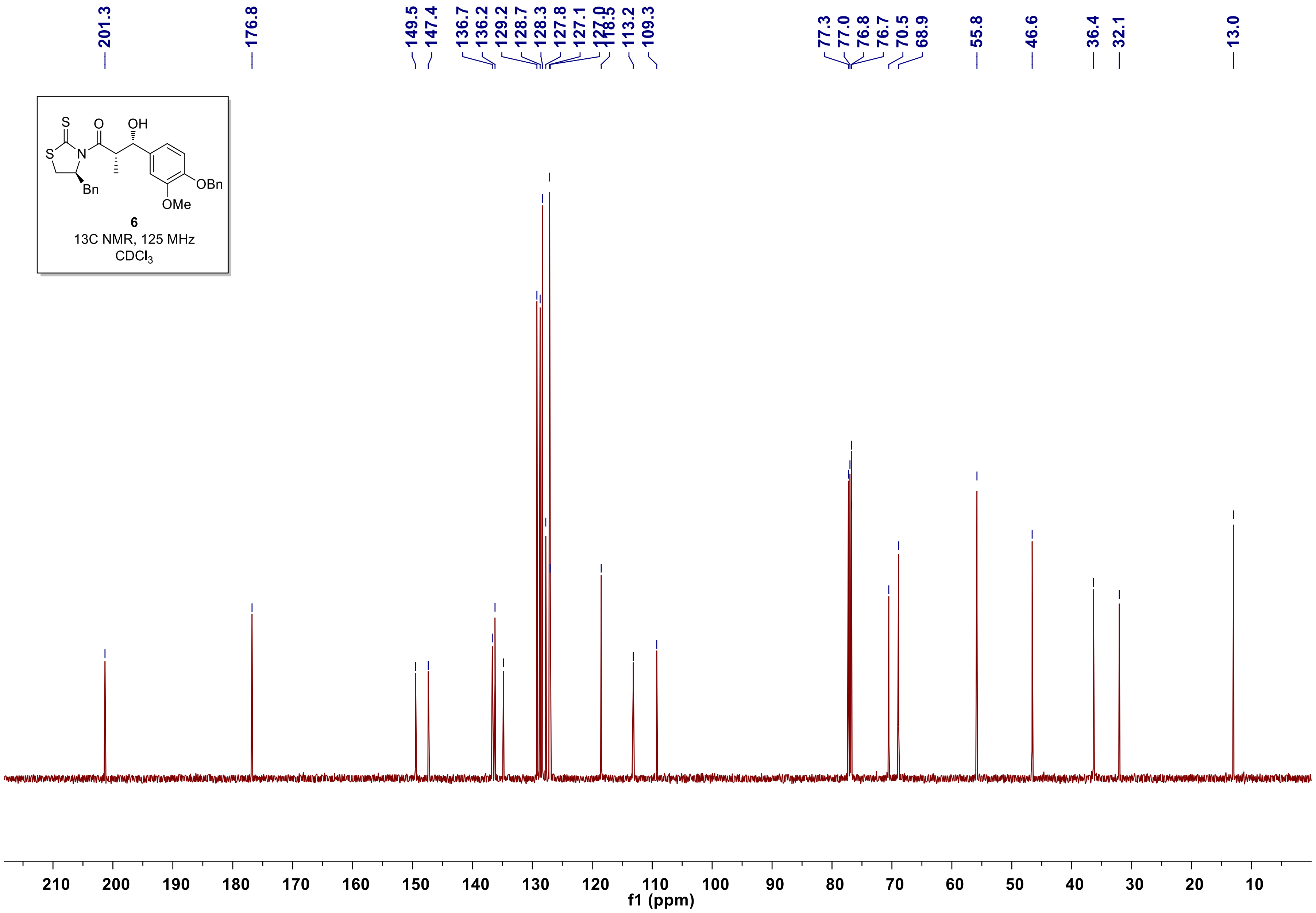
201.3
176.8
149.5
147.4
136.7
136.2
129.2
128.7
128.3
127.8
127.1
127.0
118.5
113.2
109.3
77.3
77.0
76.8
76.7
70.5
68.9
55.8
46.6
36.4
32.1
13.0
S
O
OH
S
N
Bn
OBn
OMe
6
13C NMR, 125 MHz
CDCl3
f1 (ppm)

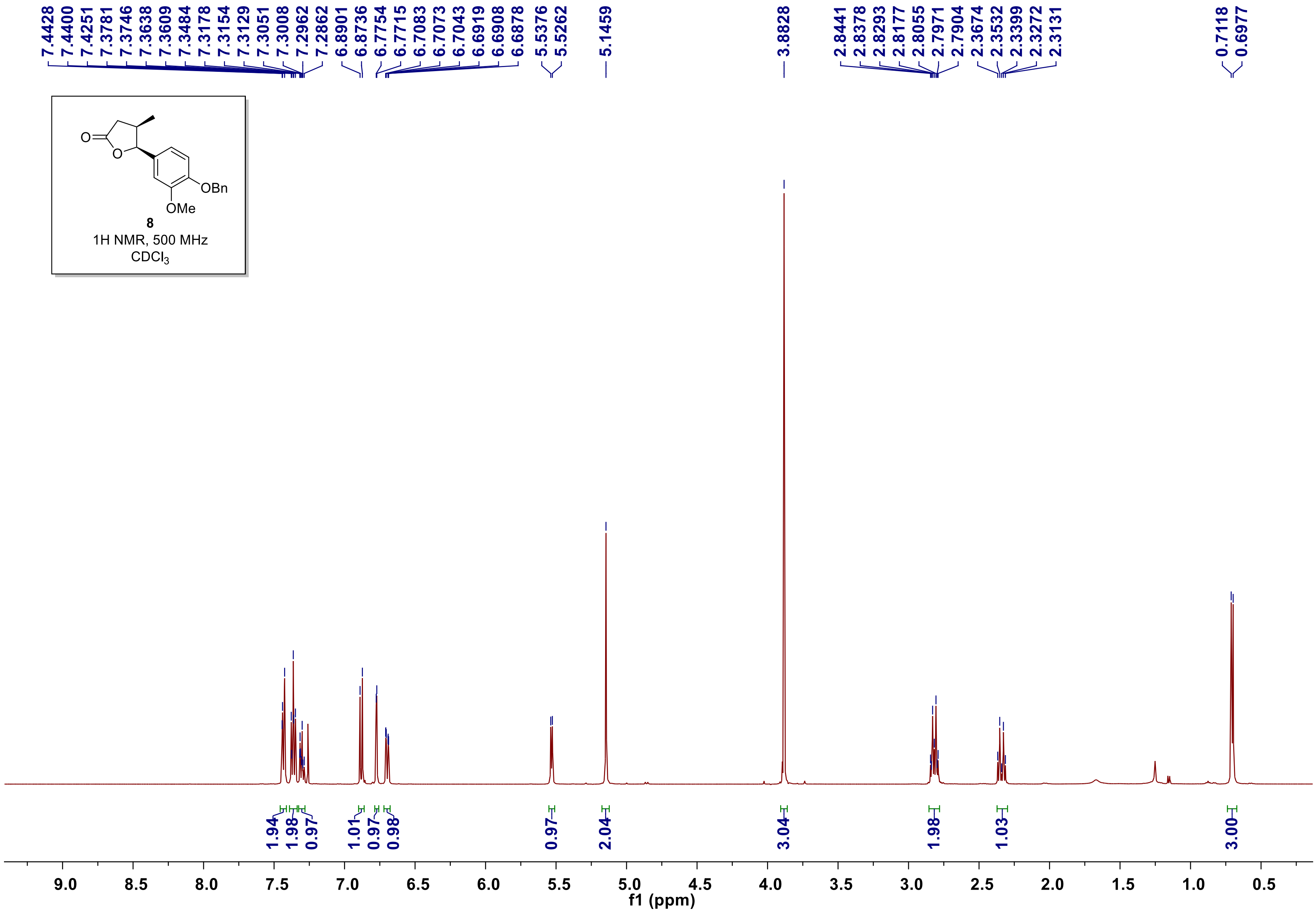

7.4428
7.4400
7.4251
7.3781
7.3746
7.3638
7.3609
7.3484
7.3178
7.3154
7.3129
7.3051
7.3008
7.2962
7.2862
6.8901
6.8736
6.7754
6.7715
6.7083
6.7073
6.7043
6.6919
6.6908
6.6878
5.5376
5.5262
5.1459
3.8828
2.8441
2.8378
2.8293
2.8177
2.8055
2.7971
2.7904
2.3674
2.3532
2.3399
2.3272
2.3131
0.7118
0.6977
O
O
OBn
OMe
8
1H NMR, 500 MHz
CDCl3
1.94
1.98
0.97
1.01
0.97
0.98
0.97
2.04
3.04
1.98
1.03
3.00
9.0
8.5
8.0
7.5
7.0
6.5
6.0
5.5
5.0
4.5
4.0
3.5
3.0
2.5
2.0
1.5
1.0
0.5
f1 (ppm)

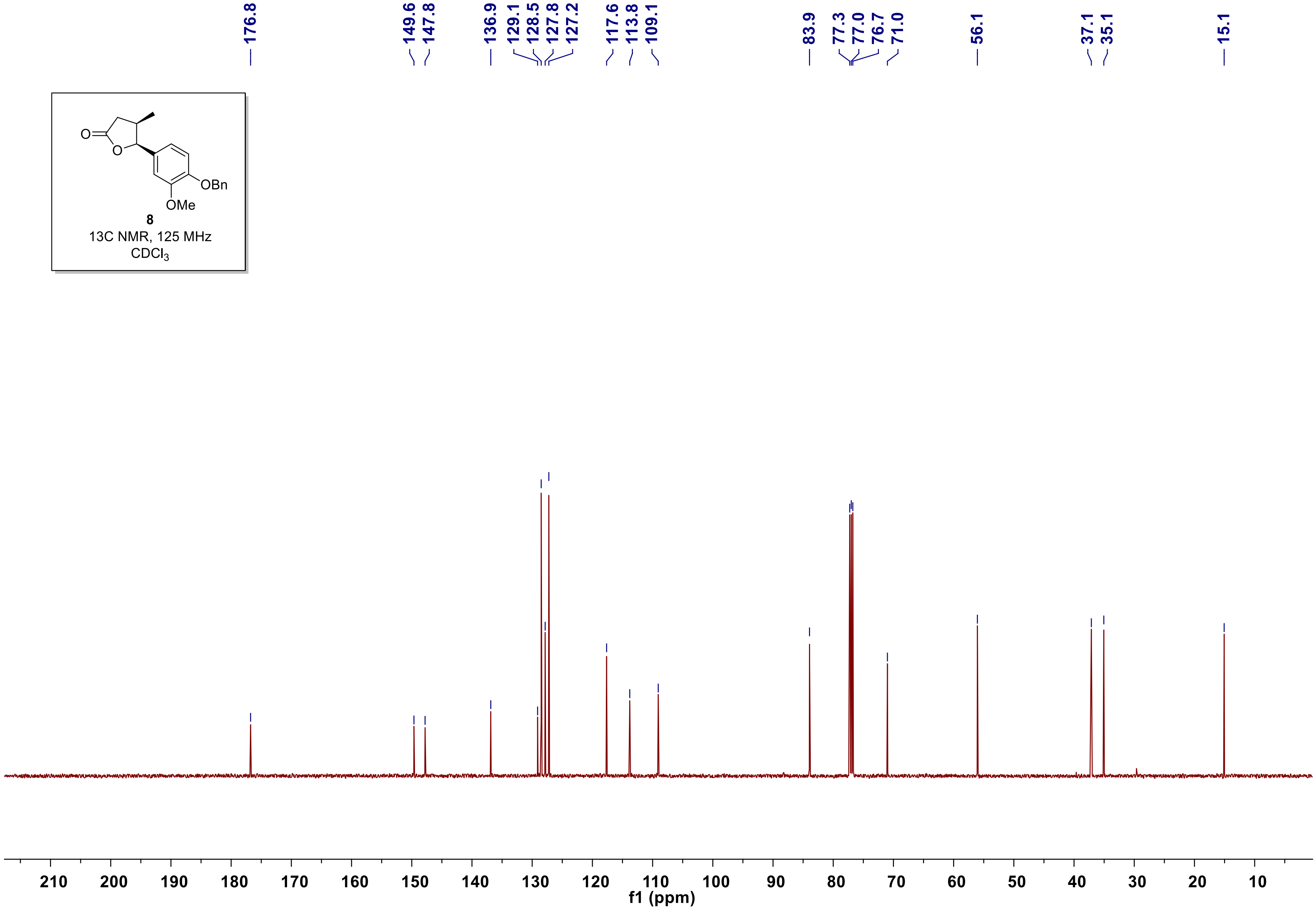
176.8
149.6
147.8
136.9
129.1
128.5
127.8
127.2
117.6
113.8
109.1
83.9
77.3
77.0
76.7
71.0
56.1
37.1
35.1
15.1
O
O
OBn
OMe
8
13C NMR, 125 MHz
$CDCl_3$
210 200 190 180 170 160 150 140 130 120 110 100 90 80 70 60 50 40 30 20 10
f1 (ppm)

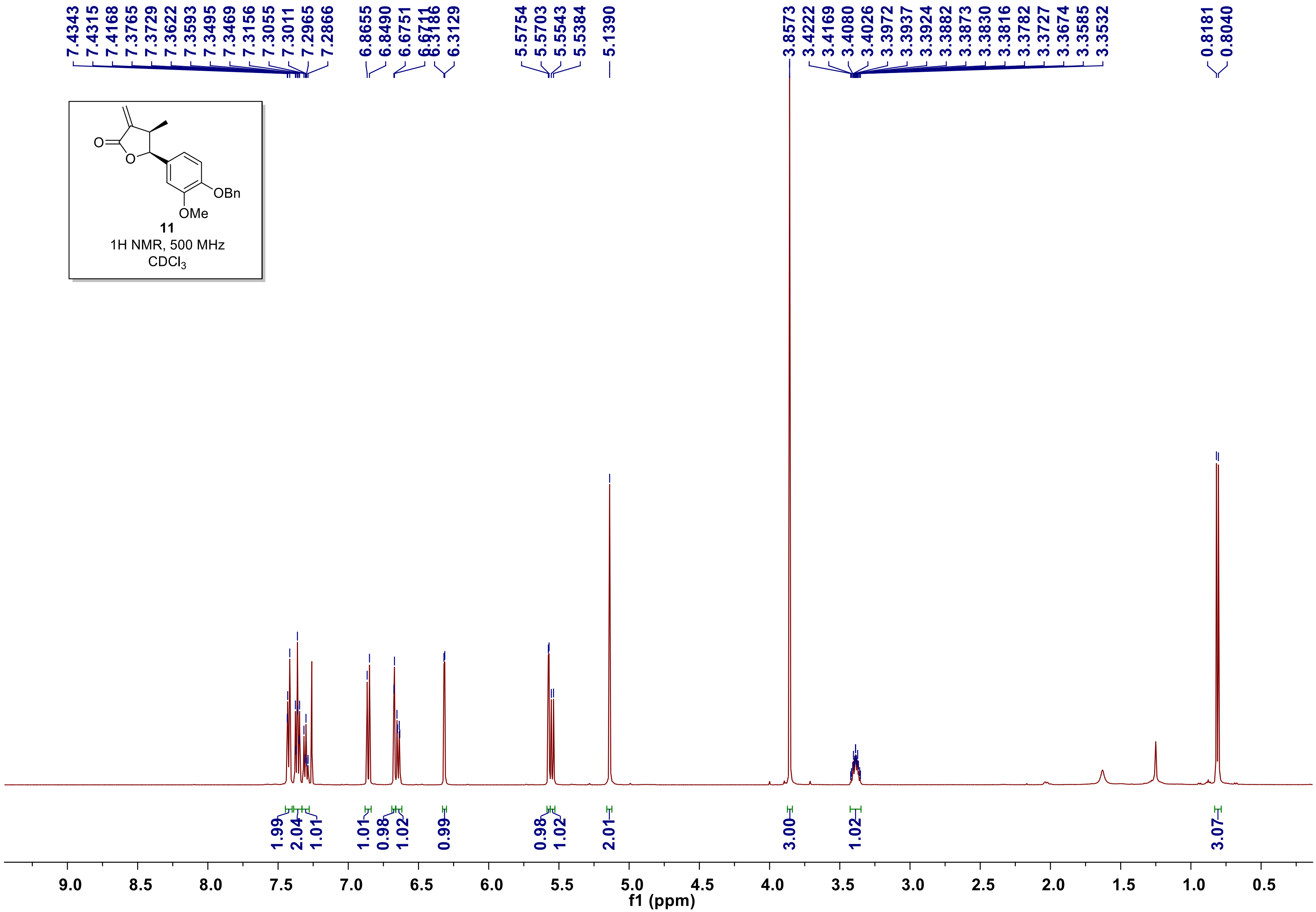

7.4343
7.4315
7.4168
7.3765
7.3729
7.3622
7.3593
7.3495
7.3469
7.3156
7.3055
7.3011
7.2965
7.2866
6.8655
6.8490
6.6751
6.6711
6.3186
6.3129
5.5754
5.5703
5.5543
5.5384
5.1390
3.8573
3.4222
3.4169
3.4080
3.4026
3.3972
3.3937
3.3924
3.3882
3.3873
3.3830
3.3816
3.3782
3.3727
3.3674
3.3585
3.3532
0.8181
0.8040
O
O
OBn
OMe
11
1H NMR, 500 MHz
CDCl3
1.99
2.04
1.01
1.01
0.98
1.02
0.99
0.98
1.02
2.01
3.00
1.02
3.07
9.0
8.5
8.0
7.5
7.0
6.5
6.0
5.5
5.0
4.5
4.0
3.5
3.0
2.5
2.0
1.5
1.0
0.5
f1 (ppm)

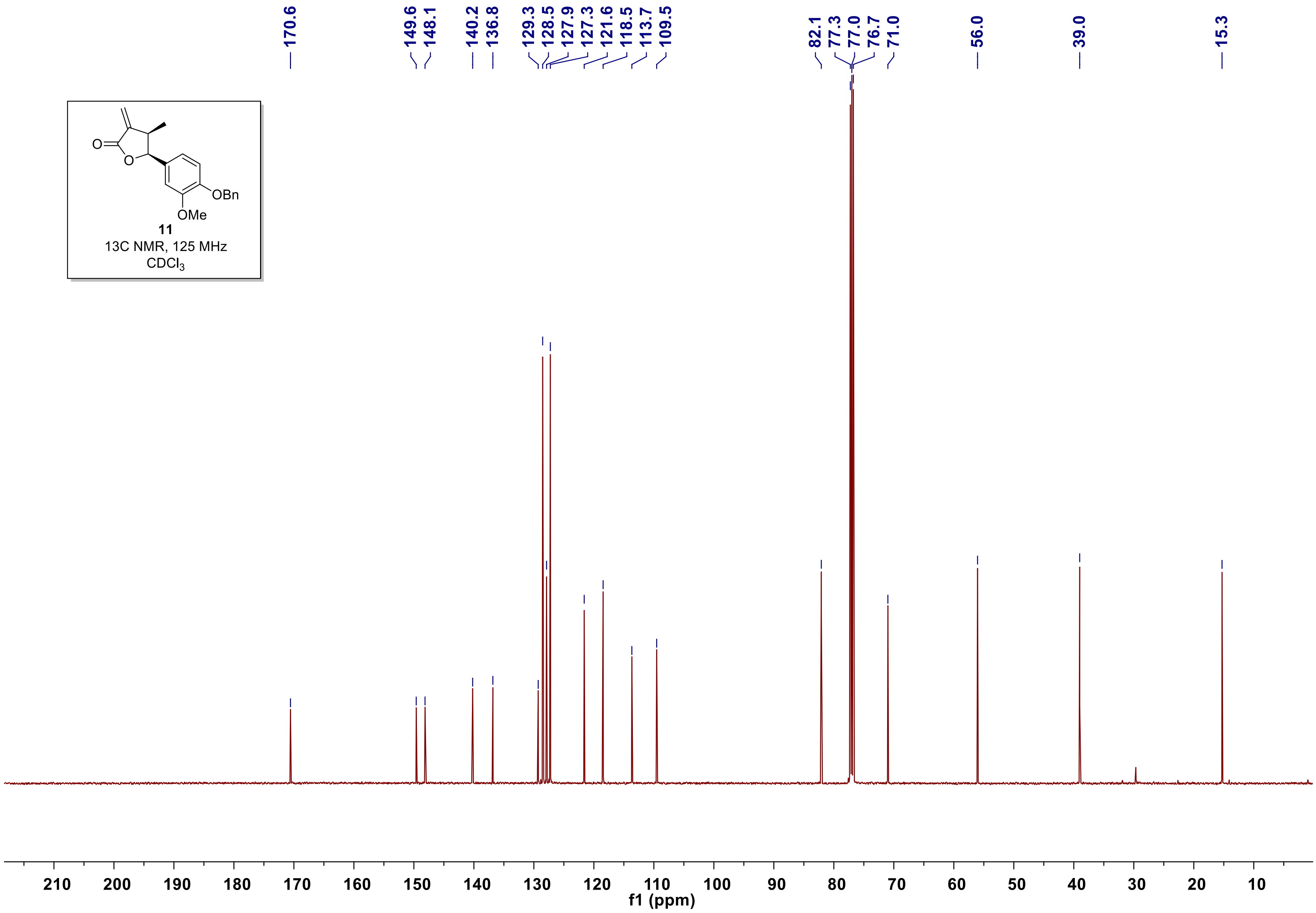
170.6
149.6
148.1
140.2
136.8
129.3
128.5
127.9
127.3
121.6
118.5
113.7
109.5
82.1
77.3
77.0
76.7
71.0
56.0
39.0
15.3
O
O
OBn
OMe
11
13C NMR, 125 MHz
CDCl3
f1 (ppm)

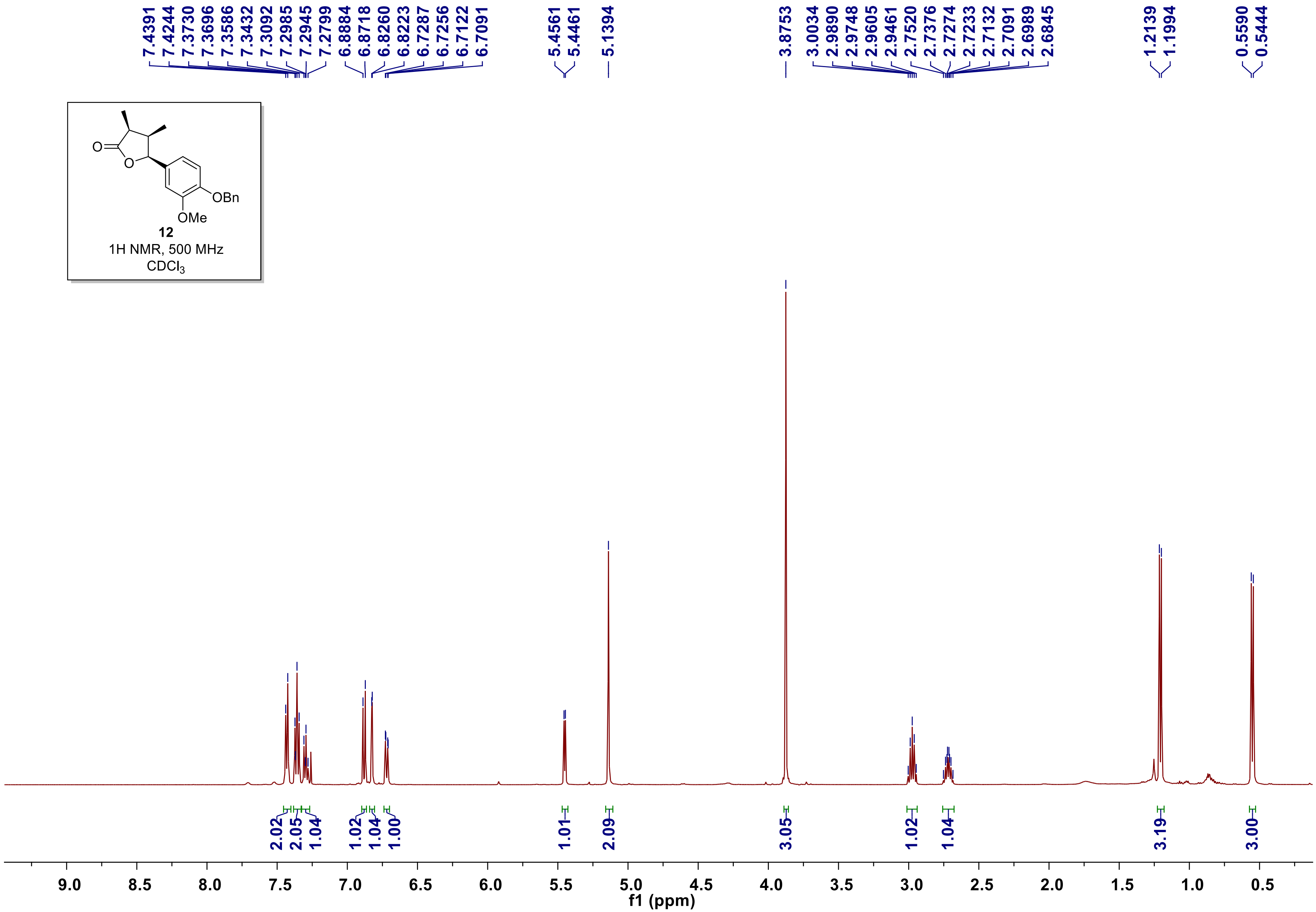
7.4391
7.4244
7.3730
7.3696
7.3586
7.3432
7.3092
7.2985
7.2945
7.2799
6.8884
6.8718
6.8260
6.8223
6.7287
6.7256
6.7122
6.7091
5.4561
5.4461
5.1394
3.8753
3.0034
2.9890
2.9748
2.9605
2.9461
2.7520
2.7376
2.7274
2.7233
2.7132
2.7091
2.6989
2.6845
1.2139
1.1994
0.5590
0.5444
O
O
OBn
OMe
12
1H NMR, 500 MHz
$CDCl_3$
2.02
2.05
1.04
1.02
1.04
1.00
1.01
2.09
3.05
1.02
1.04
3.19
3.00
9.0
8.5
8.0
7.5
7.0
6.5
6.0
5.5
5.0
4.5
4.0
3.5
3.0
2.5
2.0
1.5
1.0
0.5
f1 (ppm)

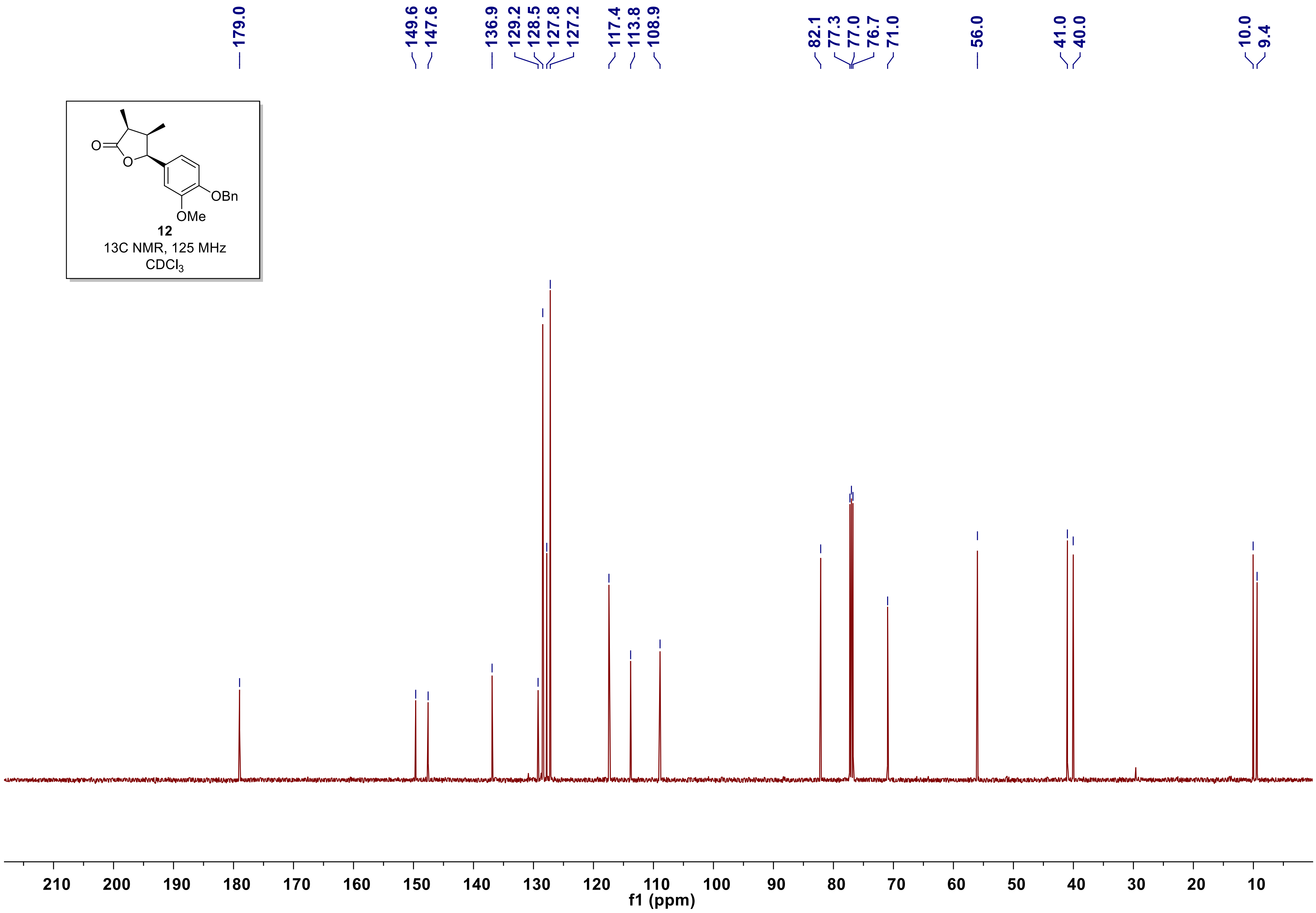
179.0
149.6
147.6
136.9
129.2
128.5
127.8
127.2
117.4
113.8
108.9
82.1
77.3
77.0
76.7
71.0
56.0
41.0
40.0
10.0
9.4
O
O
OBn
OMe
12
13C NMR, 125 MHz
CDCl3
210
200
190
180
170
160
150
140
130
120
110
100
90
80
70
60
50
40
30
20
10
f1 (ppm)

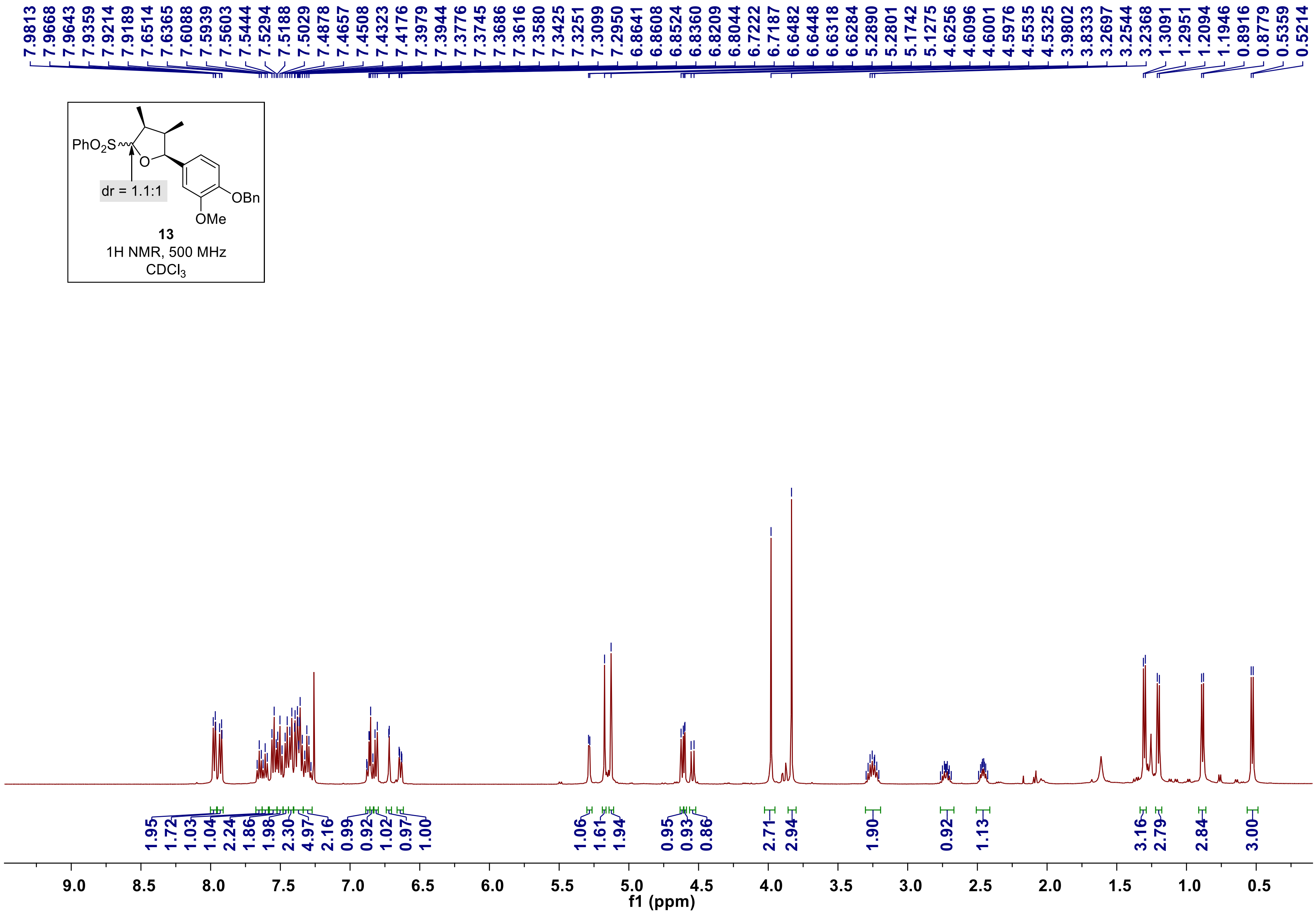
PhO2S
O
OBn
OMe
dr = 1.1:1
13
1H NMR, 500 MHz
CDCl3
f1 (ppm)

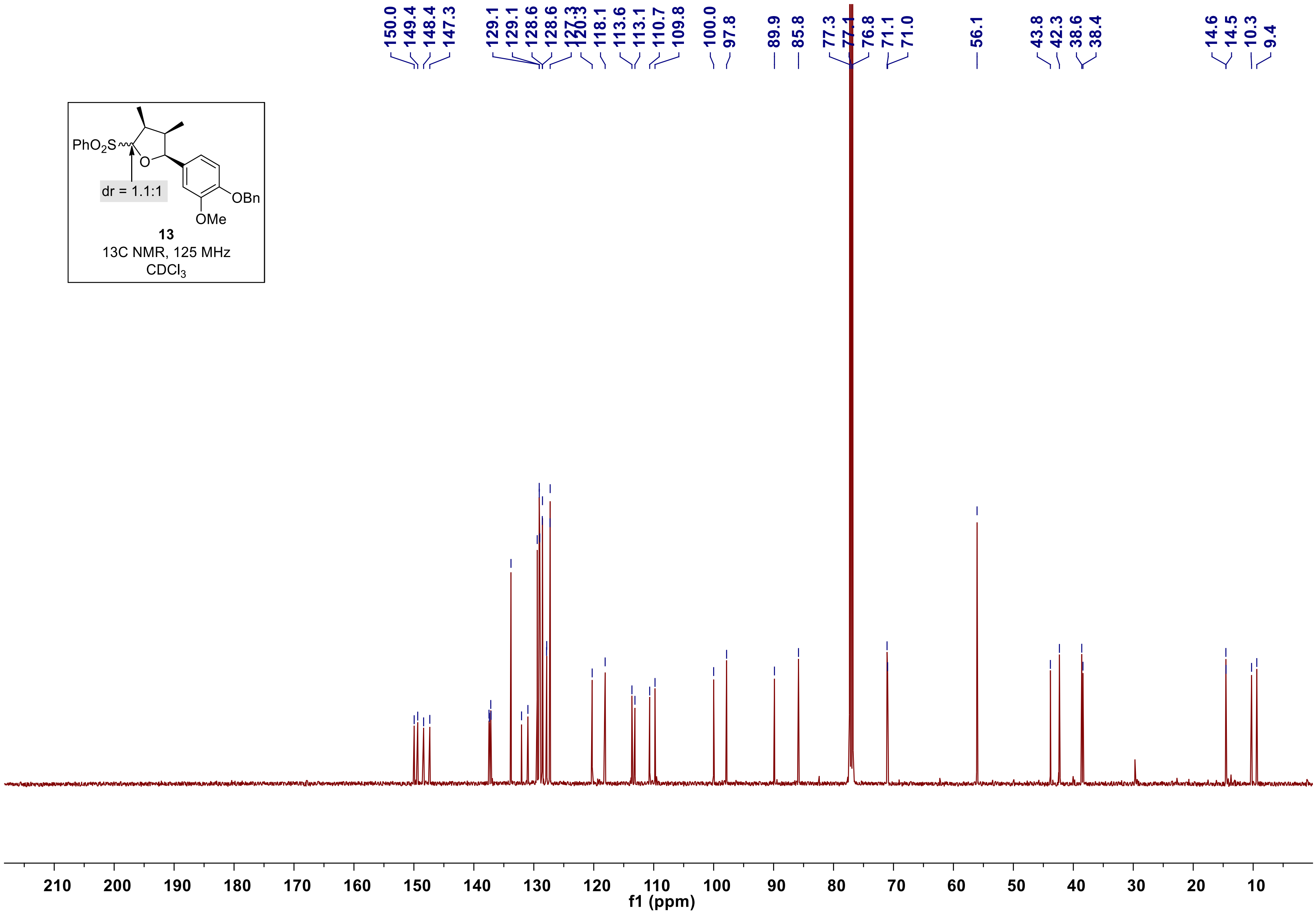
150.0
149.4
148.4
147.3
129.1
129.1
128.6
128.6
127.3
120.3
118.1
113.6
113.1
110.7
109.8
100.0
97.8
89.9
85.8
77.3
77.1
76.8
71.1
71.0
56.1
43.8
42.3
38.6
38.4
14.6
14.5
10.3
9.4
PhO2S
O
dr = 1.1:1
OBn
OMe
13
13C NMR, 125 MHz
CDCl3
210
200
190
180
170
160
150
140
130
120
110
100
90
80
70
60
50
40
30
20
10
f1 (ppm)

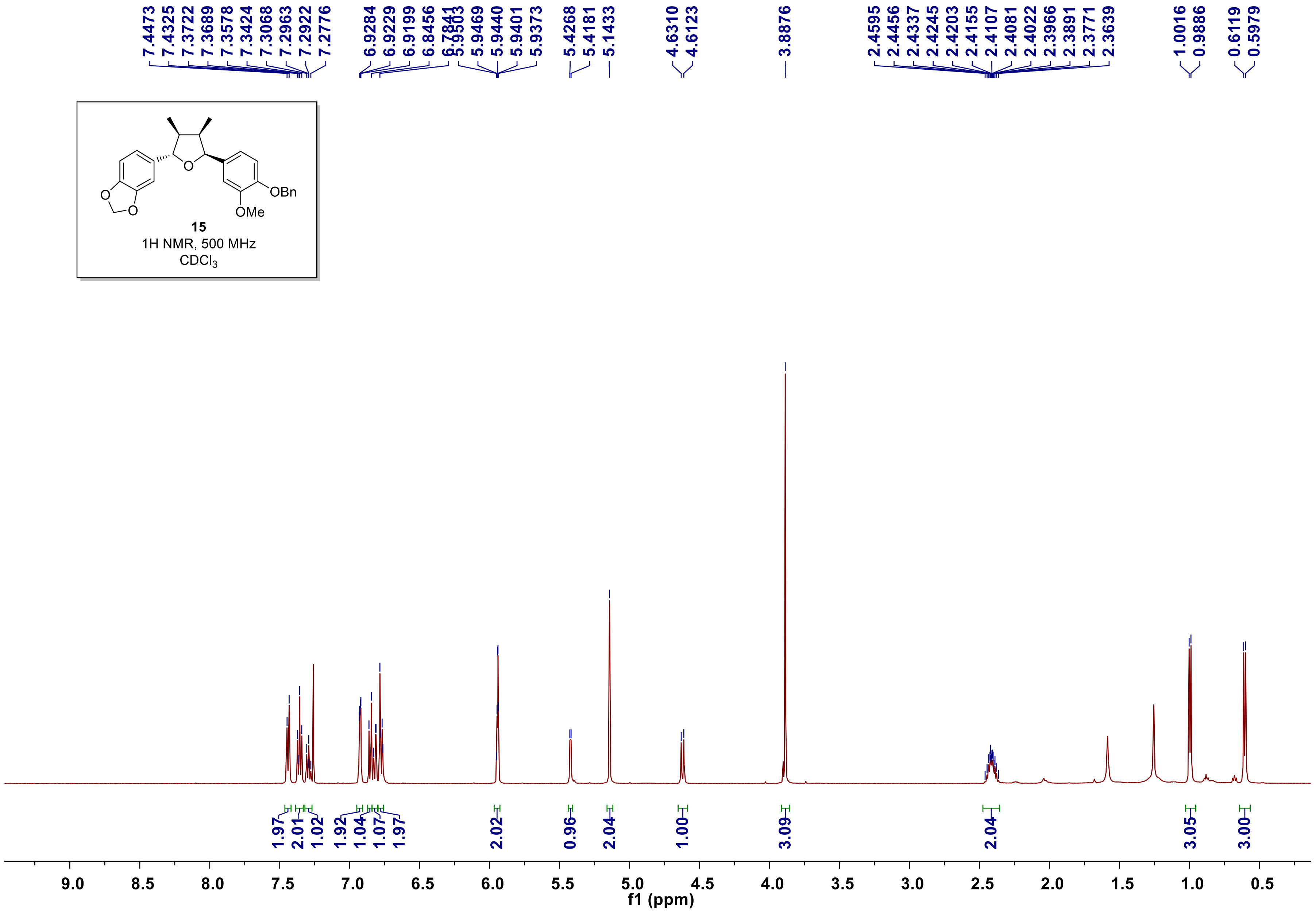
OBn
OMe
15
1H NMR, 500 MHz
$CDCl_3$
f1 (ppm)

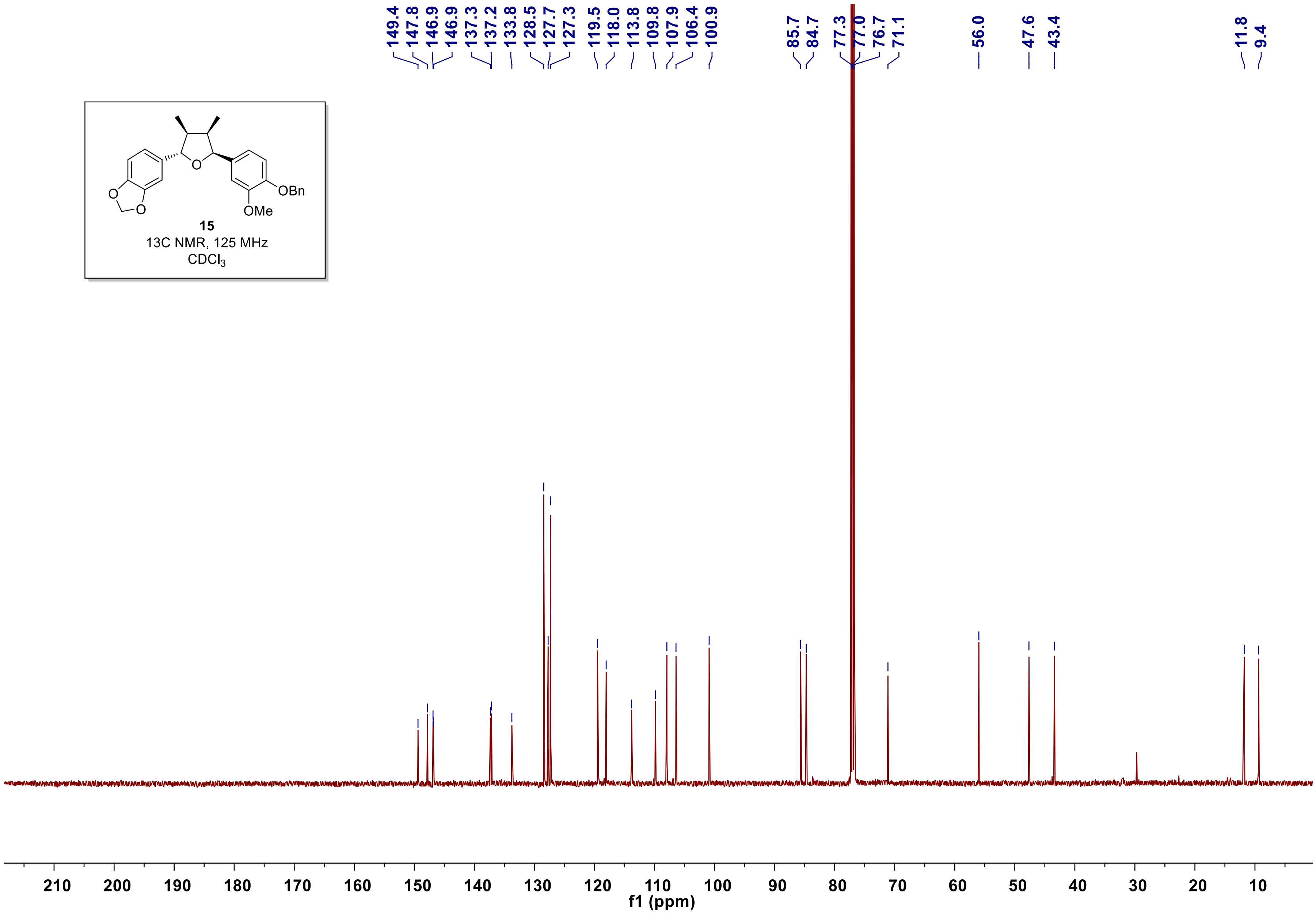
149.4
147.8
146.9
146.9
137.3
137.2
133.8
128.5
127.7
127.3
119.5
118.0
113.8
109.8
107.9
106.4
100.9
85.7
84.7
77.3
77.0
76.7
71.1
56.0
47.6
43.4
11.8
9.4
O
O
O
OBn
OMe
15
13C NMR, 125 MHz
CDCl3
210 200 190 180 170 160 150 140 130 120 110 100 90 80 70 60 50 40 30 20 10
f1 (ppm)

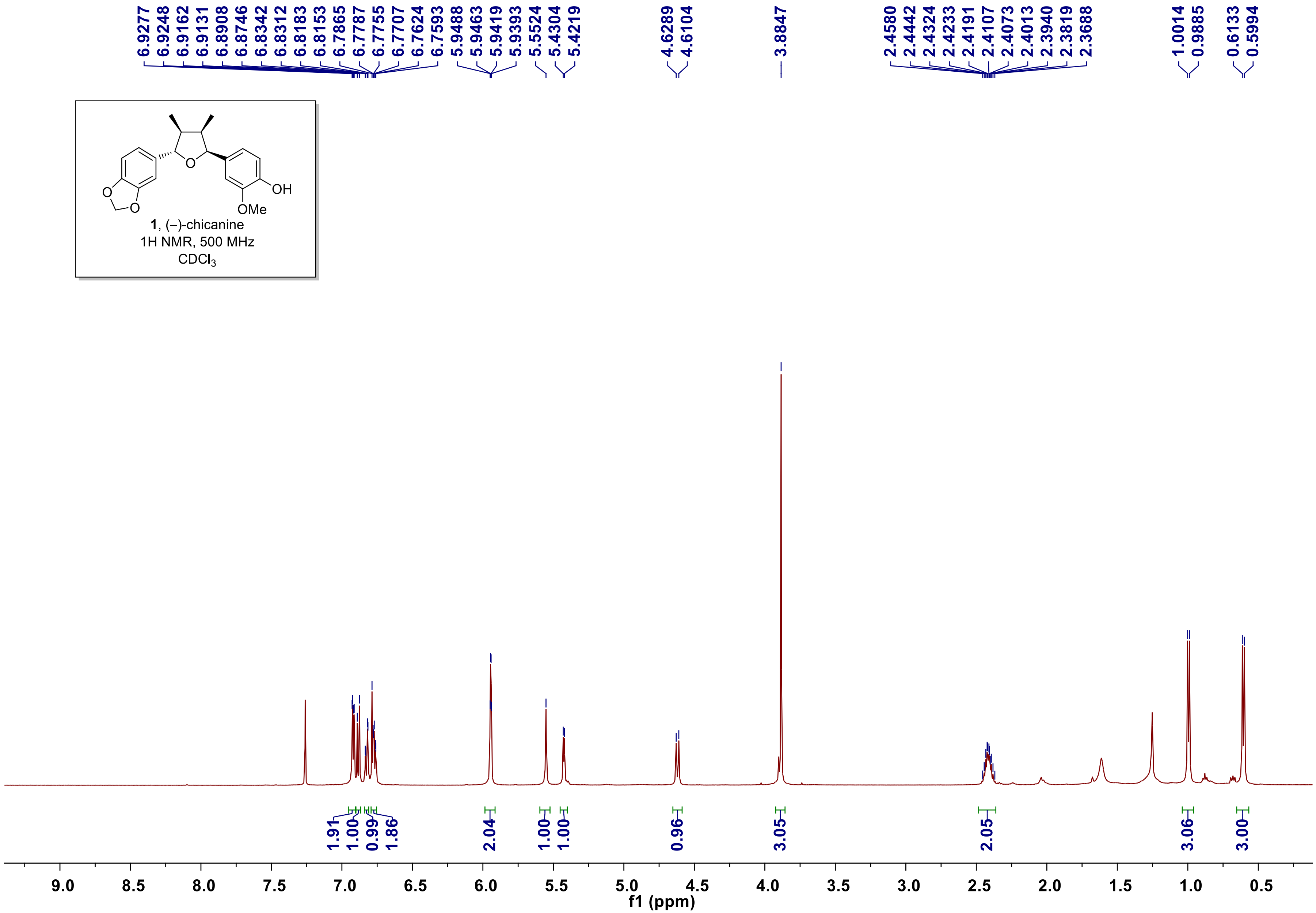
6.9277
6.9248
6.9162
6.9131
6.8908
6.8746
6.8342
6.8312
6.8183
6.8153
6.7865
6.7787
6.7755
6.7707
6.7624
6.7593
5.9488
5.9463
5.9419
5.9393
5.5524
5.4304
5.4219
4.6289
4.6104
3.8847
2.4580
2.4442
2.4324
2.4233
2.4191
2.4107
2.4073
2.4013
2.3940
2.3819
2.3688
1.0014
0.9885
0.6133
0.5994
OH
OMe
O
O
O
1, (–)-chicanine
1H NMR, 500 MHz
$CDCl_3$
1.91
1.00
0.99
1.86
2.04
1.00
1.00
0.96
3.05
2.05
3.06
3.00
9.0 8.5 8.0 7.5 7.0 6.5 6.0 5.5 5.0 4.5 4.0 3.5 3.0 2.5 2.0 1.5 1.0 0.5
f1 (ppm)

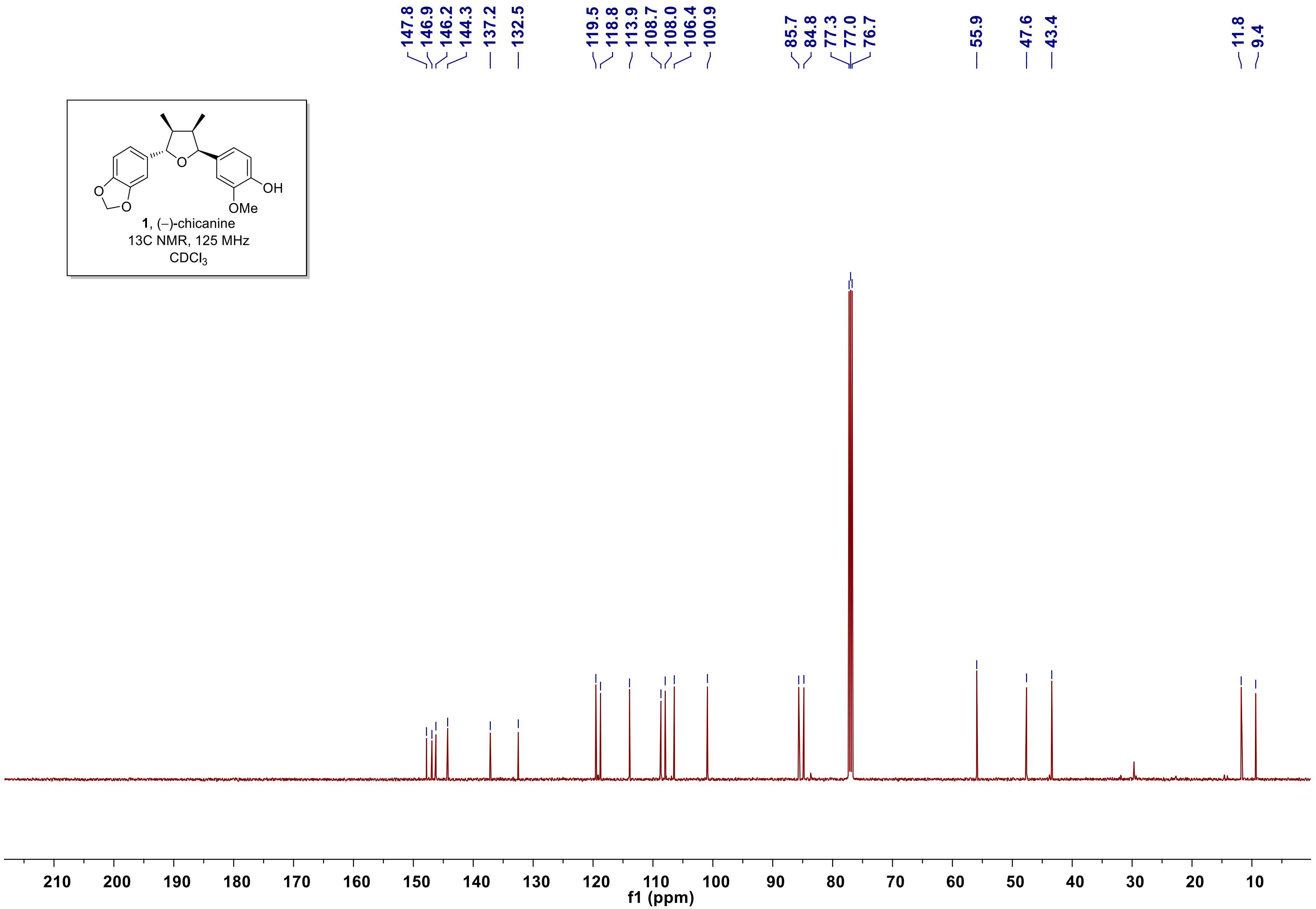
147.8
146.9
146.2
144.3
137.2
132.5
119.5
118.8
113.9
108.7
108.0
106.4
100.9
85.7
84.8
77.3
77.0
76.7
55.9
47.6
43.4
11.8
9.4
O
O
O
OH
OMe
1, (–)-chicanine
13C NMR, 125 MHz
CDCl3
210
200
190
180
170
160
150
140
130
120
110
100
90
80
70
60
50
40
30
20
10
f1 (ppm)

Fukuyama's
***synthetic* (–)-chicanine**
(400 MHz, $CDCl_3$)
Tetrahedron Lett. **2011**, *52,* 3005

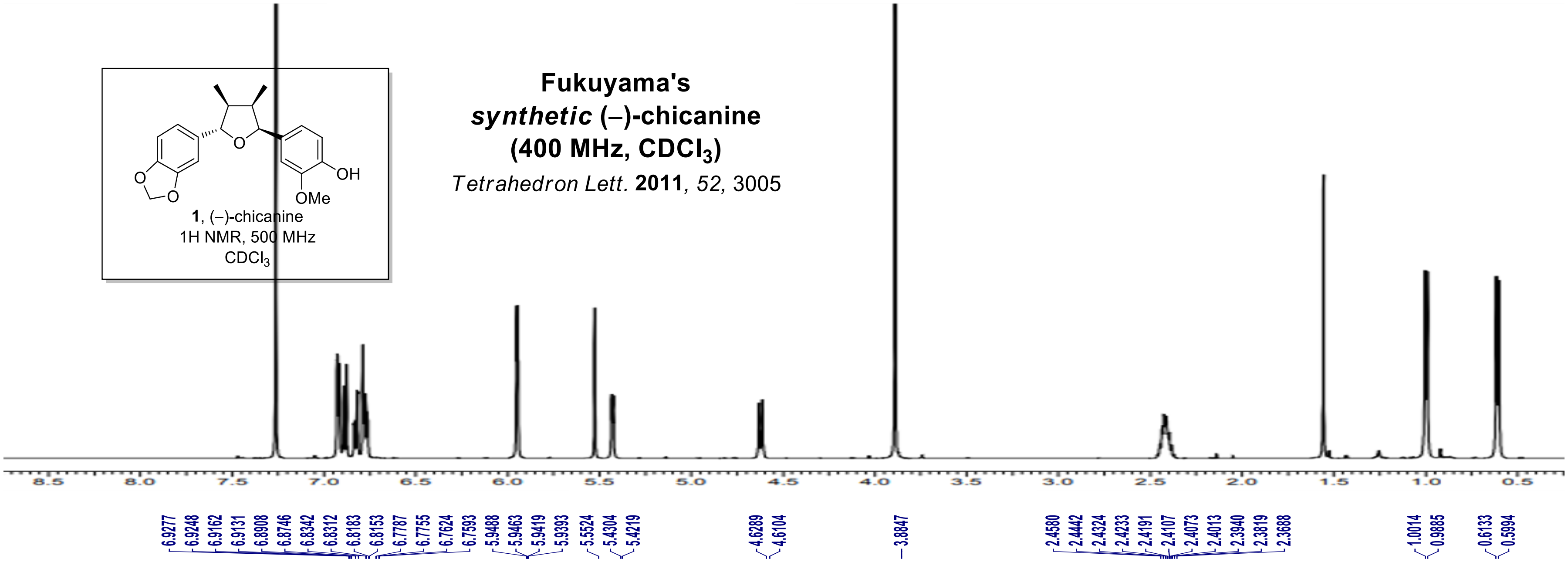

***Synthetic* (–)-chicanine**
(500 MHz, $CDCl_3$)

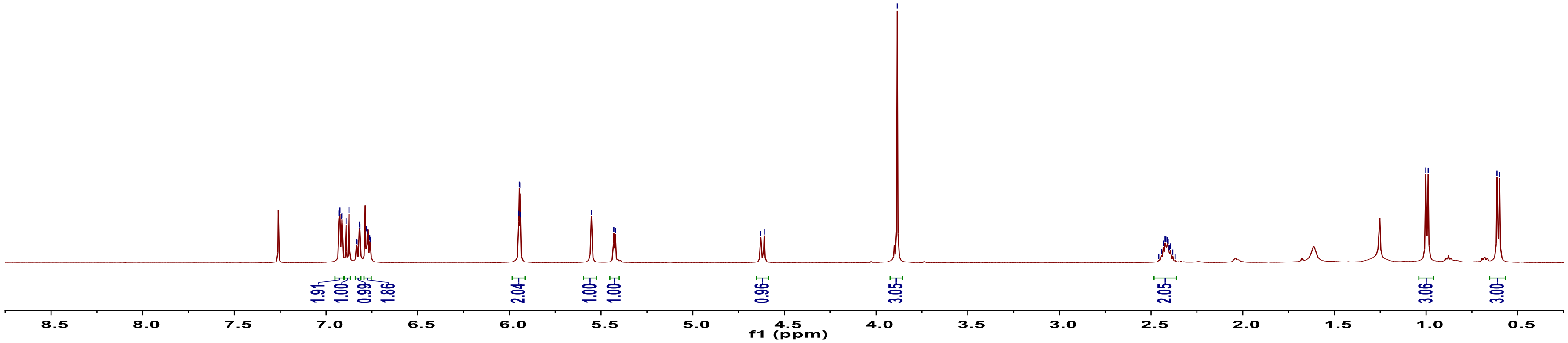

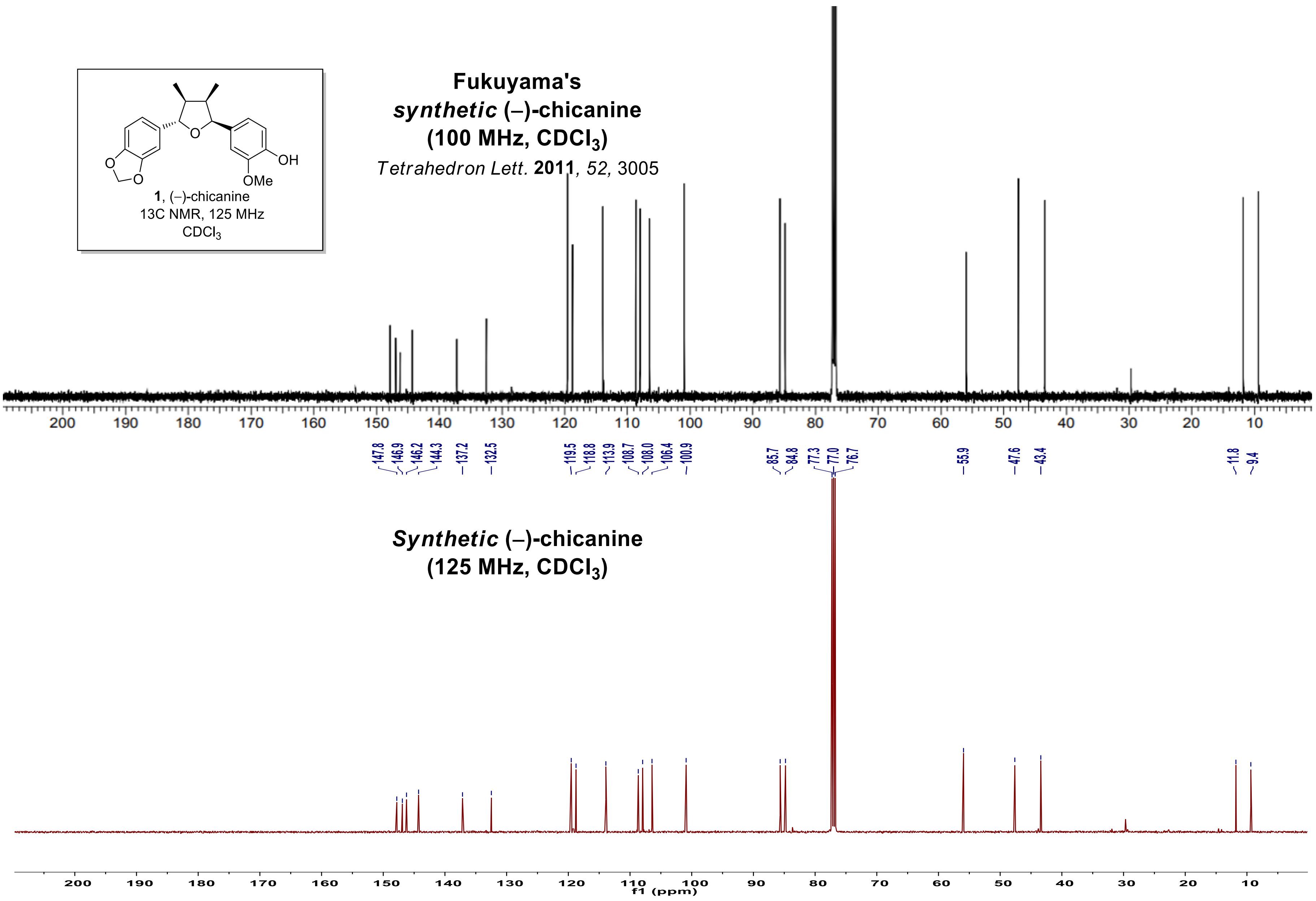

OH
OMe
O
O
O
1, (–)-chicanine
13C NMR, 125 MHz
CDCl3
Fukuyama's
synthetic (–)-chicanine
(100 MHz, CDCl3)
Tetrahedron Lett. 2011, 52, 3005
147.8 146.9 146.2 144.3 137.2 132.5 119.5 118.8 113.9 108.7 108.0 106.4 100.9 85.7 84.8 77.3 77.0 76.7 55.9 47.6 43.4 11.8 9.4
Synthetic (–)-chicanine
(125 MHz, CDCl3)
200 190 180 170 160 150 140 130 120 110 100 90 80 70 60 50 40 30 20 10
f1 (ppm)

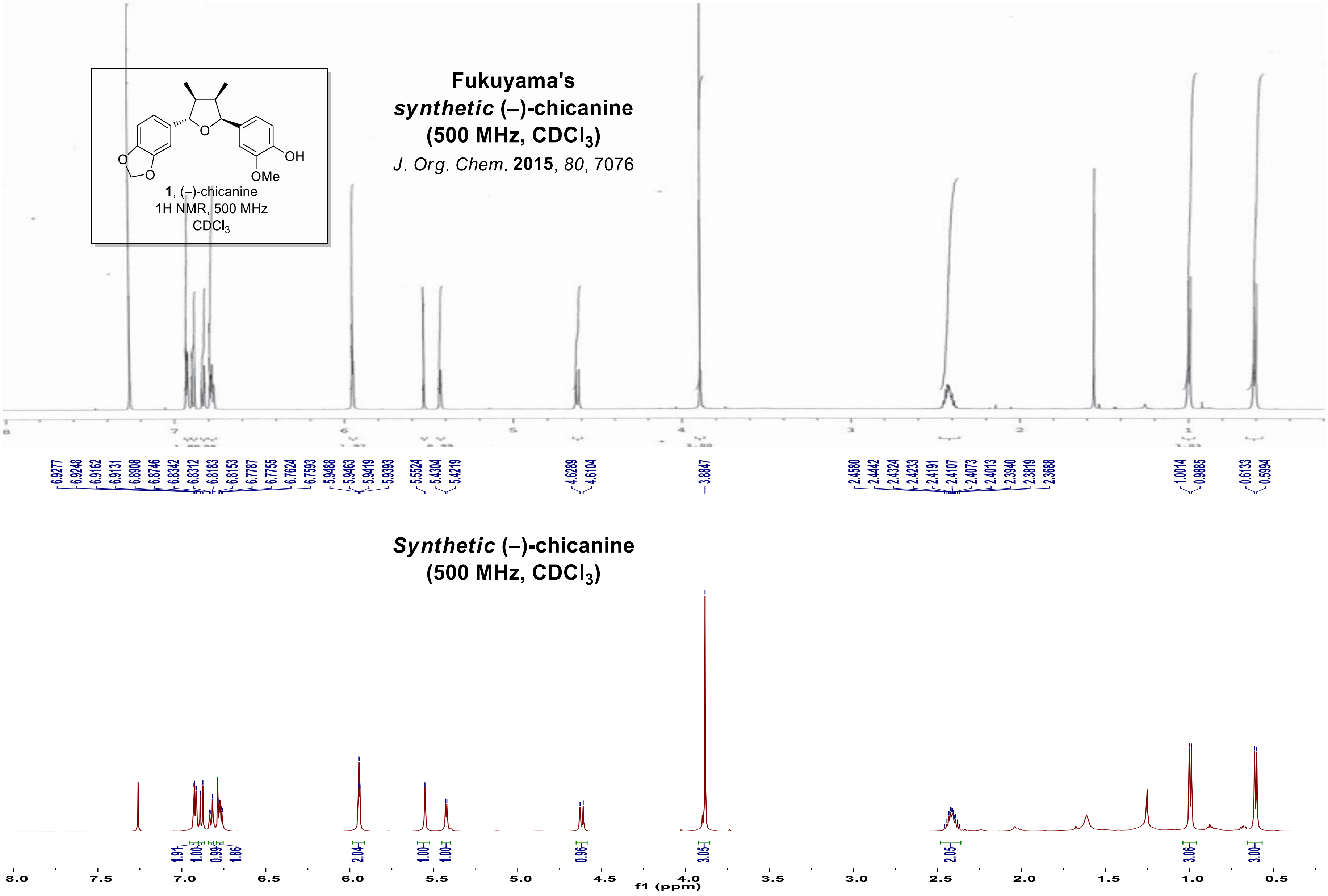
Fukuyama's
synthetic (–)-chicanine
(500 MHz, $CDCl_3$)
J. Org. Chem. **2015**, *80*, 7076
OH
OMe
O
1, (–)-chicanine
1H NMR, 500 MHz
$CDCl_3$
6.9277 6.9248 6.9162 6.9131 6.8908 6.8746 6.8342 6.8312 6.8183 6.8153 6.7787 6.7755 6.7624 6.7593 5.9488 5.9463 5.9419 5.9393 5.5524 5.4304 5.4219
4.6289 4.6104
3.8847
2.4580 2.4442 2.4324 2.4233 2.4191 2.4107 2.4073 2.4013 2.3940 2.3819 2.3688
1.0014 0.9885
0.6133 0.5994
Synthetic (–)-chicanine
(500 MHz, $CDCl_3$)
1.91 1.00 0.99 1.86 2.04 1.00 1.00 0.96 3.05 2.05 3.06 3.00
8.0 7.5 7.0 6.5 6.0 5.5 5.0 4.5 4.0 3.5 3.0 2.5 2.0 1.5 1.0 0.5
f1 (ppm)

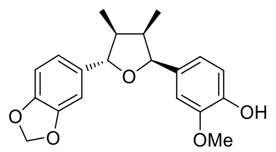

1, (–)-chicanine
13C NMR, 125 MHz
$CDCl_3$

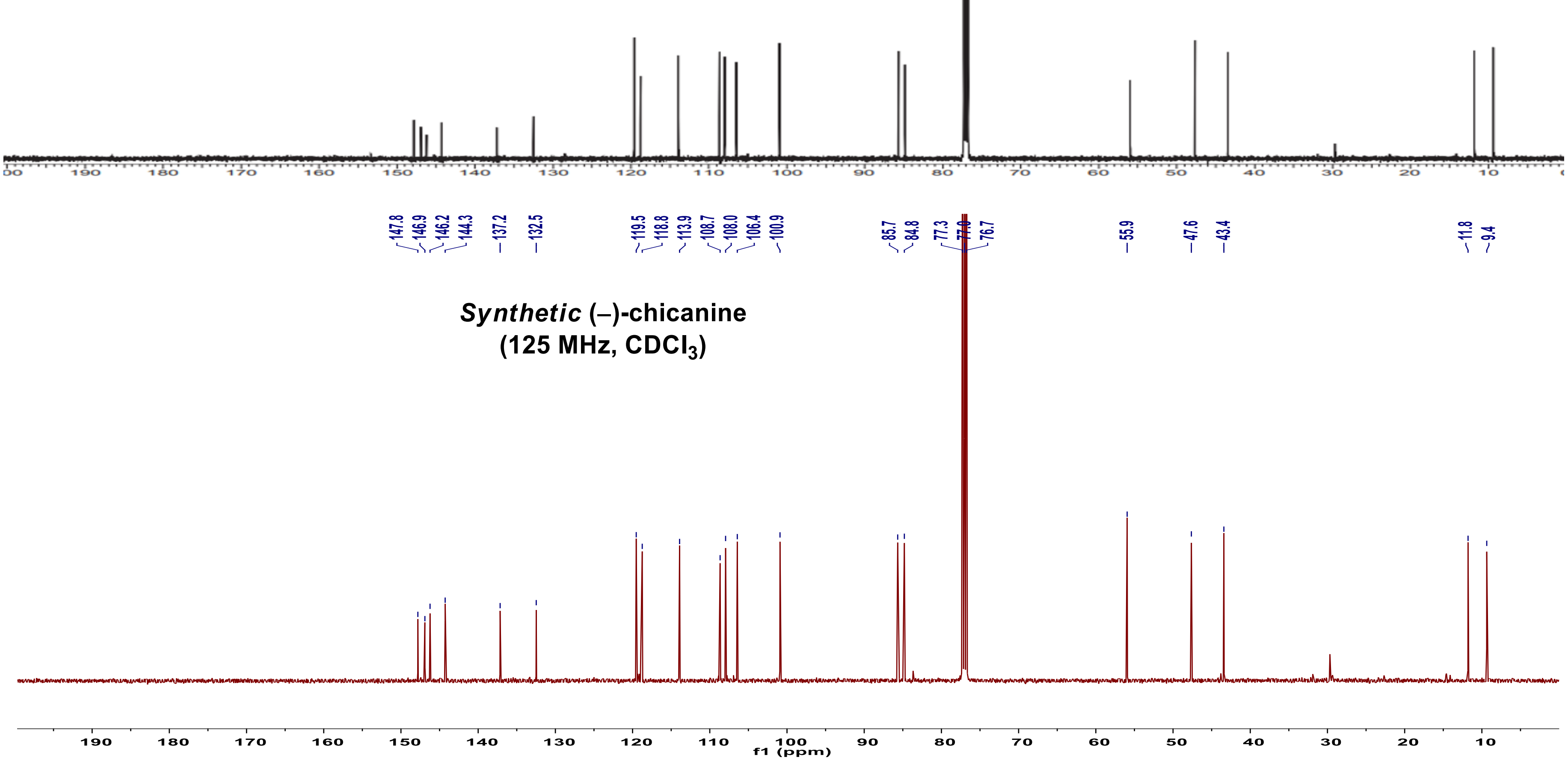

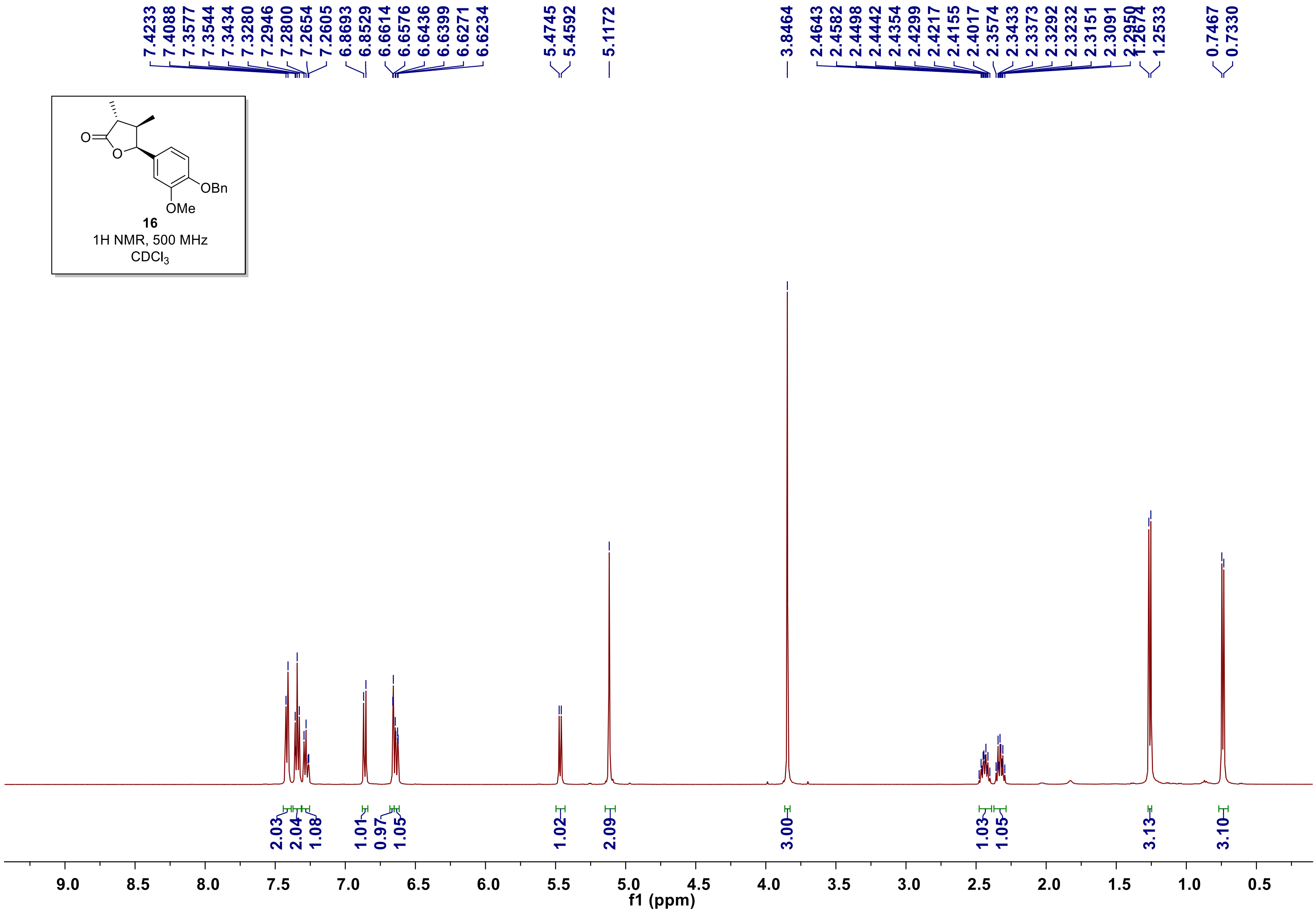
16
1H NMR, 500 MHz
CDCl3
f1 (ppm)

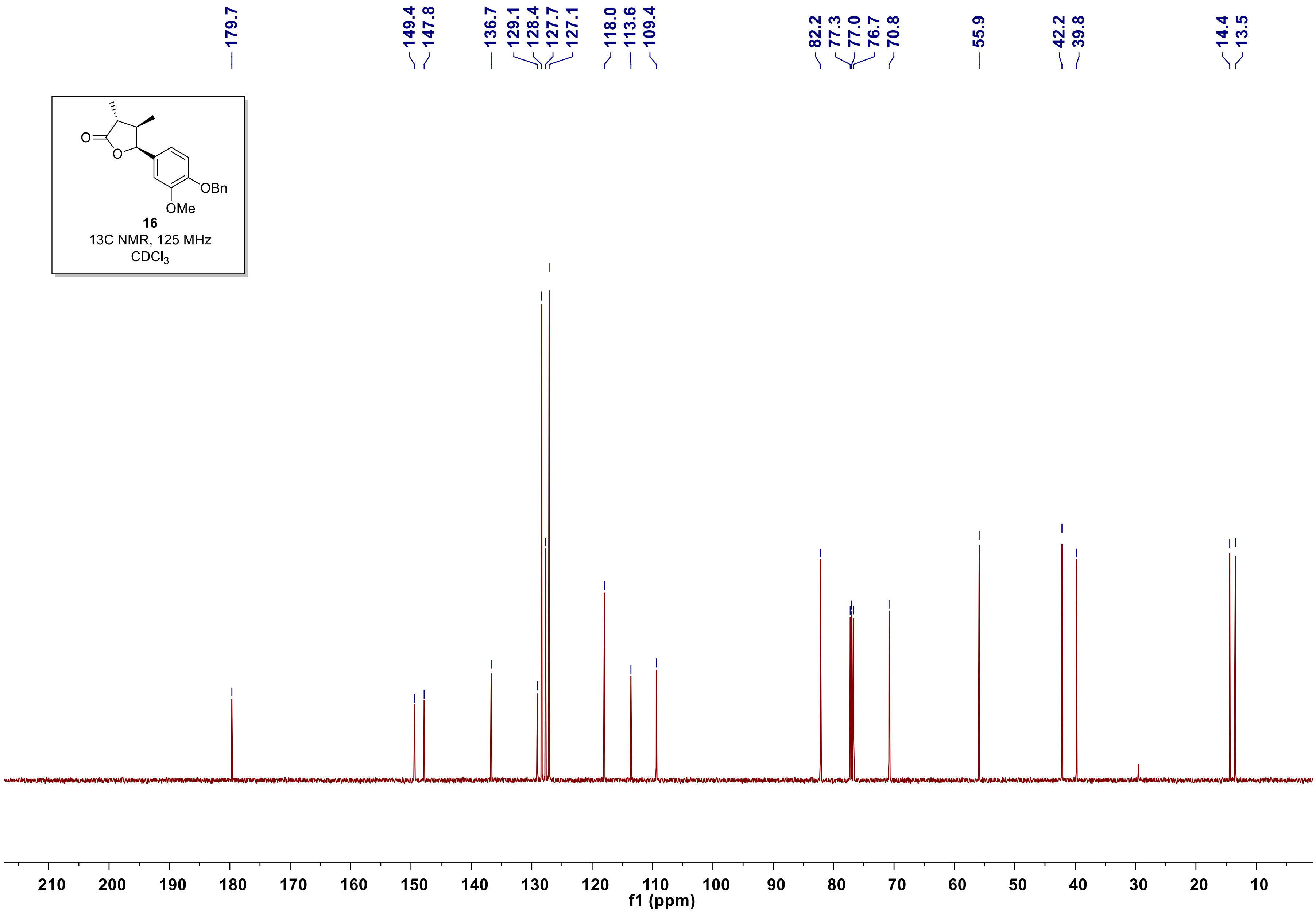
179.7
149.4
147.8
136.7
129.1
128.4
127.7
127.1
118.0
113.6
109.4
82.2
77.3
77.0
76.7
70.8
55.9
42.2
39.8
14.4
13.5
O
O
OBn
OMe
16
13C NMR, 125 MHz
$CDCl_3$
210
200
190
180
170
160
150
140
130
120
110
100
90
80
70
60
50
40
30
20
10
f1 (ppm)

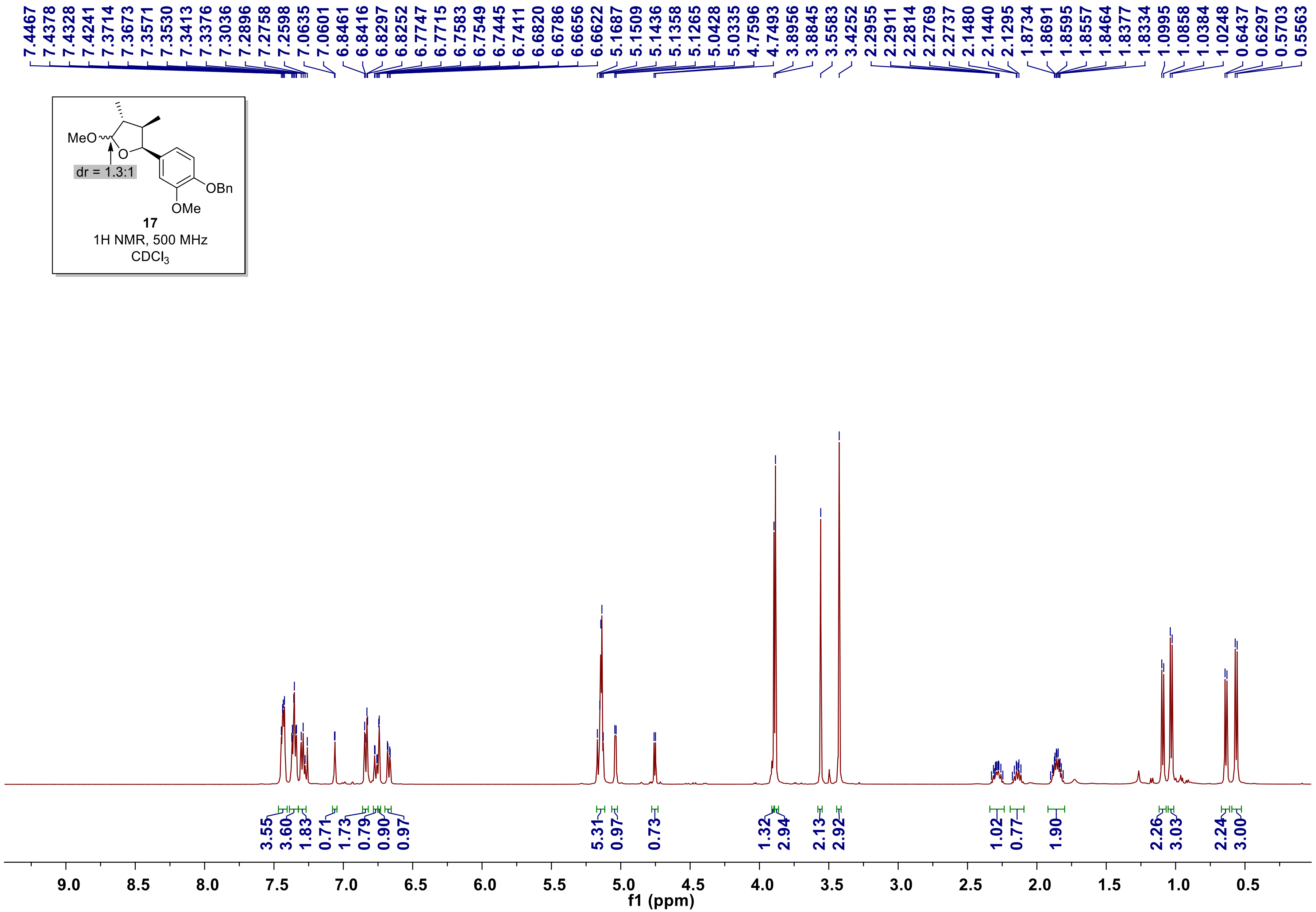
MeO
O
dr = 1.3:1
OBn
OMe
17
1H NMR, 500 MHz
$CDCl_3$
f1 (ppm)

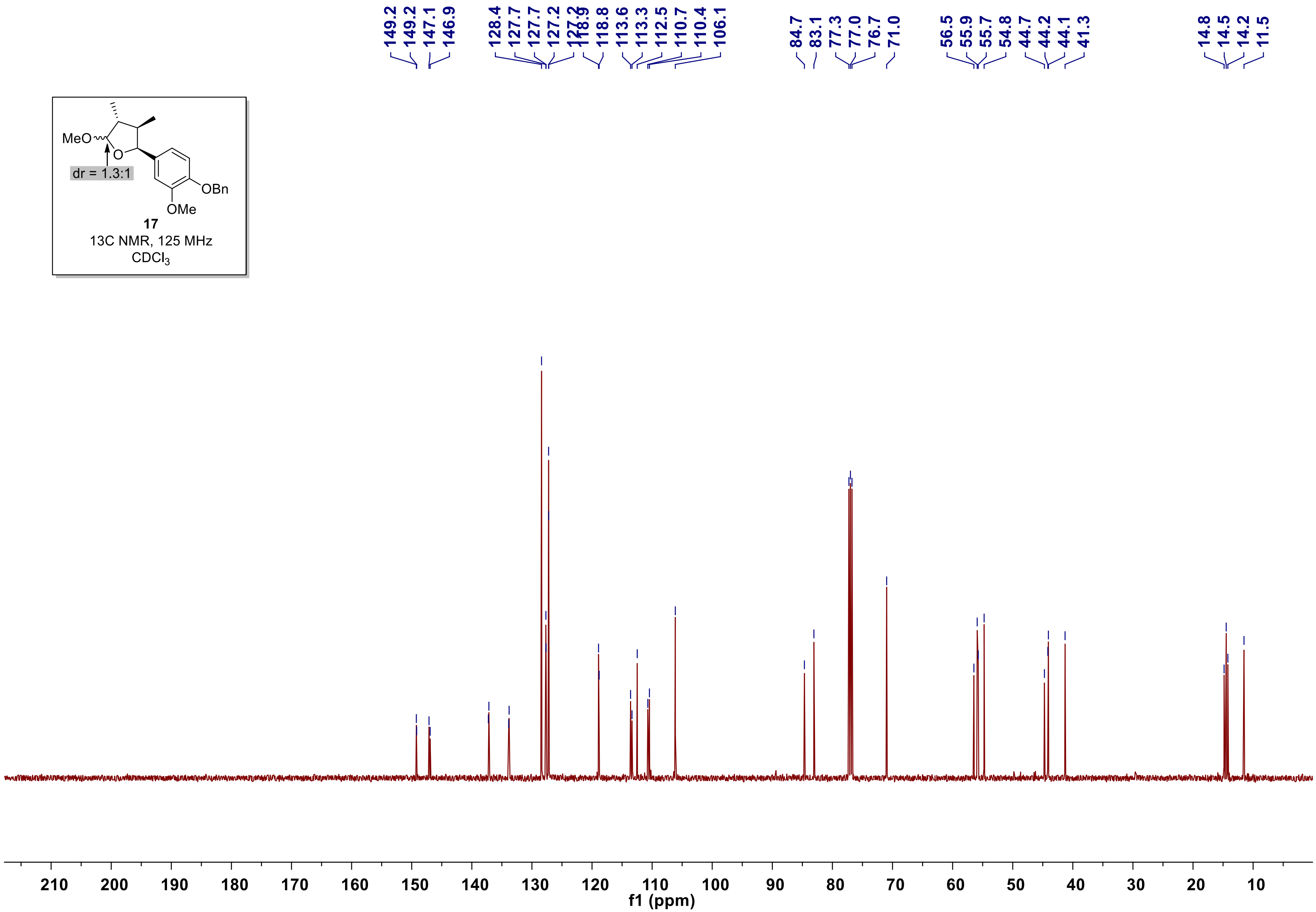

149.2
149.2
147.1
146.9
128.4
127.7
127.7
127.2
127.2
118.9
118.8
113.6
113.3
112.5
110.7
110.4
106.1
84.7
83.1
77.3
77.0
76.7
71.0
56.5
55.9
55.7
54.8
44.7
44.2
44.1
41.3
14.8
14.5
14.2
11.5
MeO
O
OBn
OMe
dr = 1.3:1
17
13C NMR, 125 MHz
CDCl3
210 200 190 180 170 160 150 140 130 120 110 100 90 80 70 60 50 40 30 20 10
f1 (ppm)

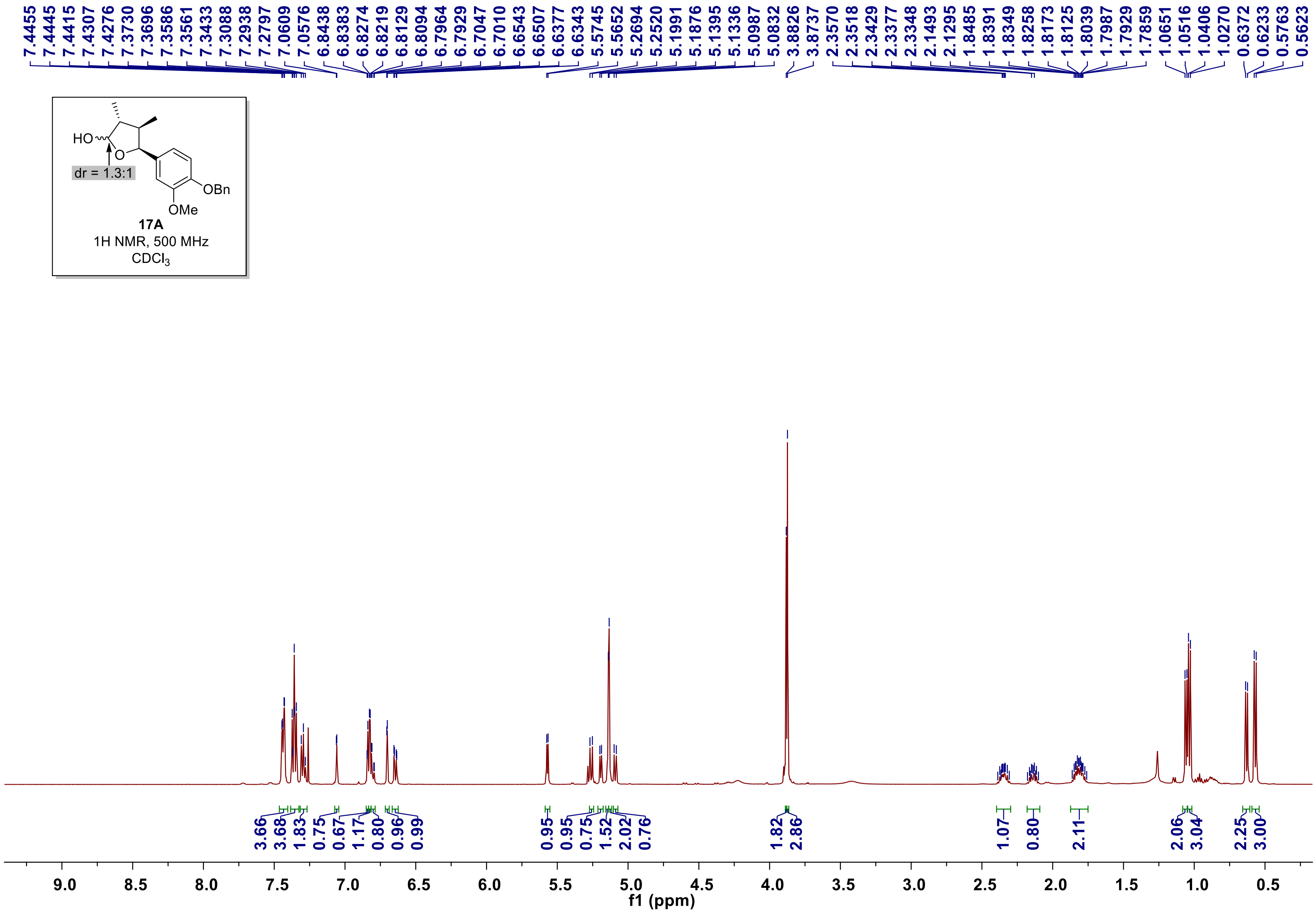
7.4455 7.4445 7.4415 7.4307 7.4276 7.3730 7.3696 7.3586 7.3561 7.3433 7.3088 7.2938 7.2797 7.0609 7.0576 6.8438 6.8383 6.8274 6.8219 6.8129 6.8094 6.7964 6.7929 6.7047 6.7010 6.6543 6.6507 6.6377 6.6343 5.5745 5.5652 5.2694 5.2520 5.1991 5.1876 5.1395 5.1336 5.0987 5.0832 3.8826 3.8737 2.3570 2.3518 2.3429 2.3377 2.3348 2.1493 2.1295 1.8485 1.8391 1.8349 1.8258 1.8173 1.8125 1.8039 1.7987 1.7929 1.7859 1.0651 1.0516 1.0406 1.0270 0.6372 0.6233 0.5763 0.5623
HO
O
OBn
OMe
dr = 1.3:1
17A
1H NMR, 500 MHz
CDCl3
3.66 3.68 1.83 0.75 0.67 1.17 0.80 0.96 0.99 0.95 0.95 0.75 1.52 2.02 0.76 1.82 2.86 1.07 0.80 2.11 2.06 3.04 2.25 3.00
9.0 8.5 8.0 7.5 7.0 6.5 6.0 5.5 5.0 4.5 4.0 3.5 3.0 2.5 2.0 1.5 1.0 0.5
f1 (ppm)

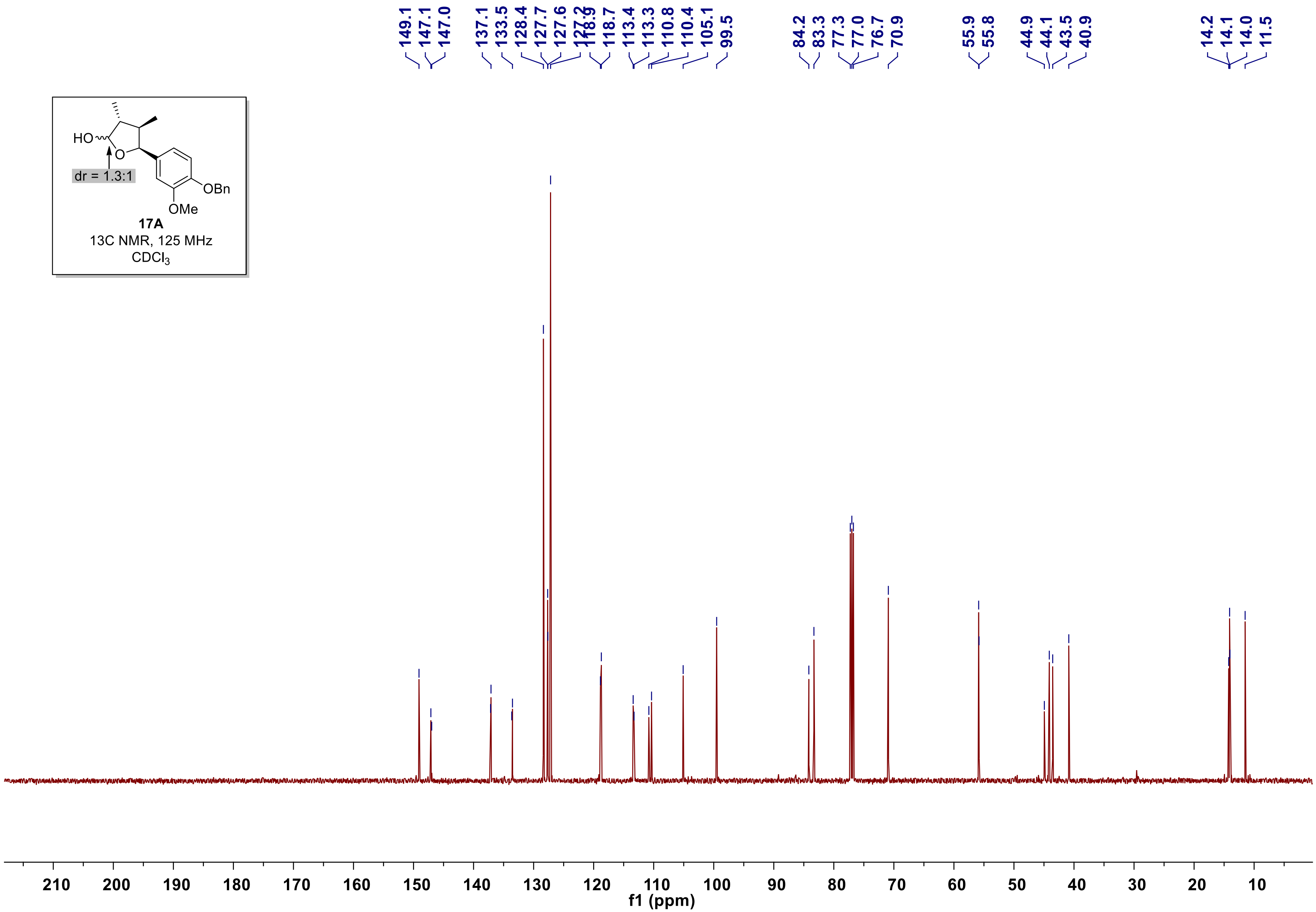
149.1
147.1
147.0
137.1
133.5
128.4
127.7
127.6
127.2
118.9
118.7
113.4
113.3
110.8
110.4
105.1
99.5
84.2
83.3
77.3
77.0
76.7
70.9
55.9
55.8
44.9
44.1
43.5
40.9
14.2
14.1
14.0
11.5
HO
O
OBn
OMe
dr = 1.3:1
17A
13C NMR, 125 MHz
CDCl3
210
200
190
180
170
160
150
140
130
120
110
100
90
80
70
60
50
40
30
20
10
f1 (ppm)

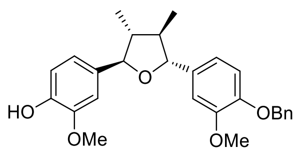

18A
1H NMR, 500 MHz
$CDCl_3$

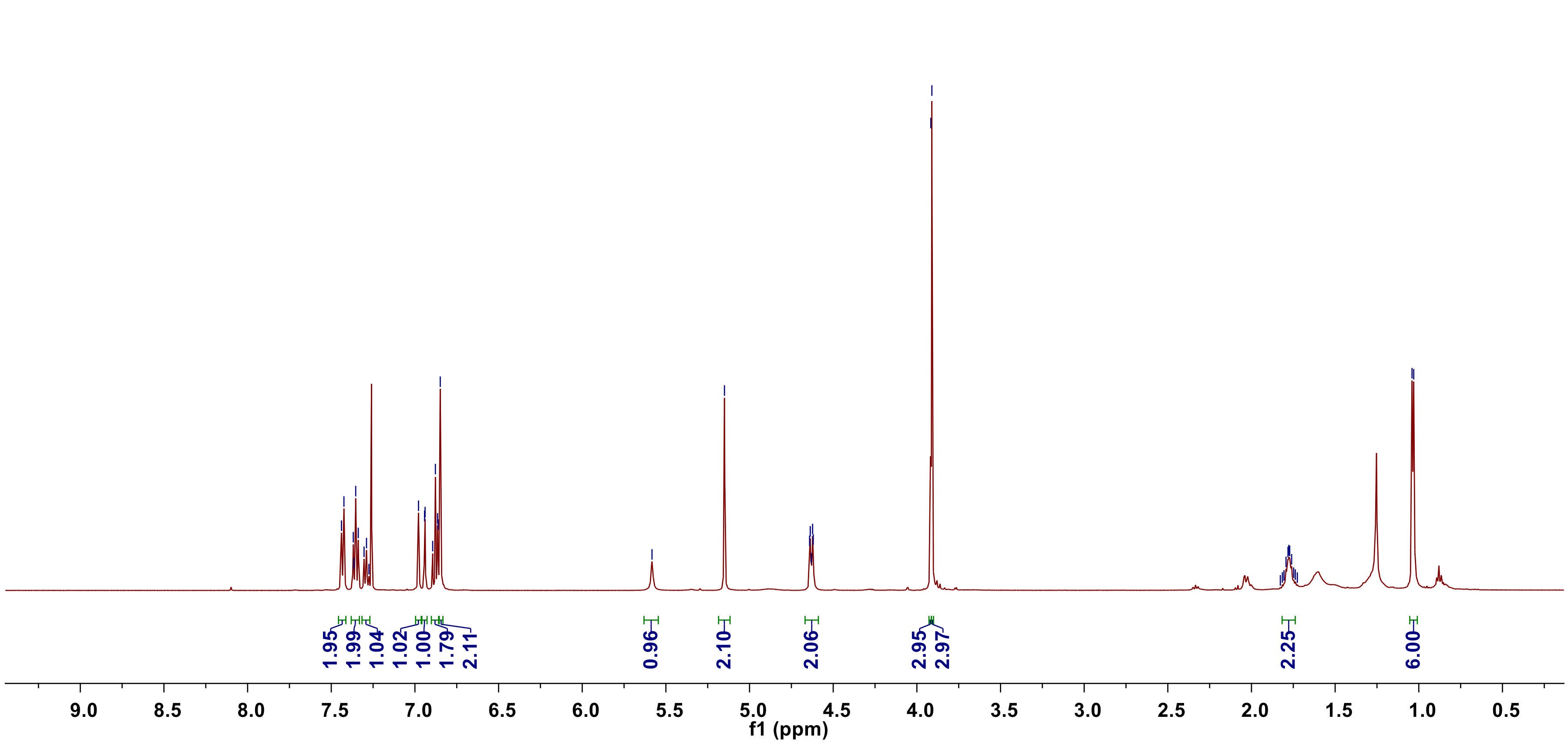

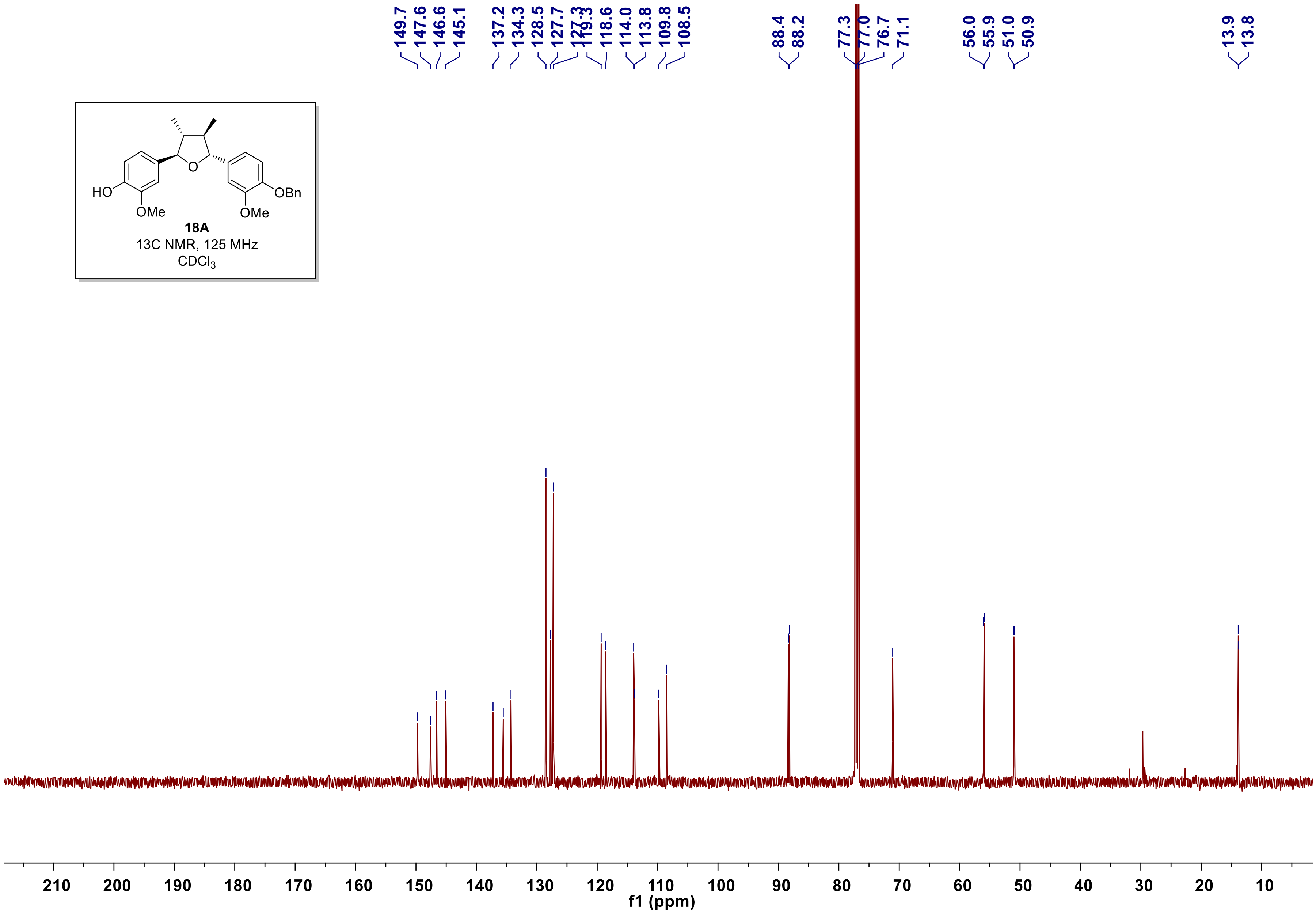

149.7
147.6
146.6
145.1
137.2
134.3
128.5
127.7
127.3
119.3
118.6
114.0
113.8
109.8
108.5
88.4
88.2
77.3
77.0
76.7
71.1
56.0
55.9
51.0
50.9
13.9
13.8
HO
OMe
O
OMe
OBn
18A
13C NMR, 125 MHz
$CDCl_3$
210
200
190
180
170
160
150
140
130
120
110
100
90
80
70
60
50
40
30
20
10
f1 (ppm)

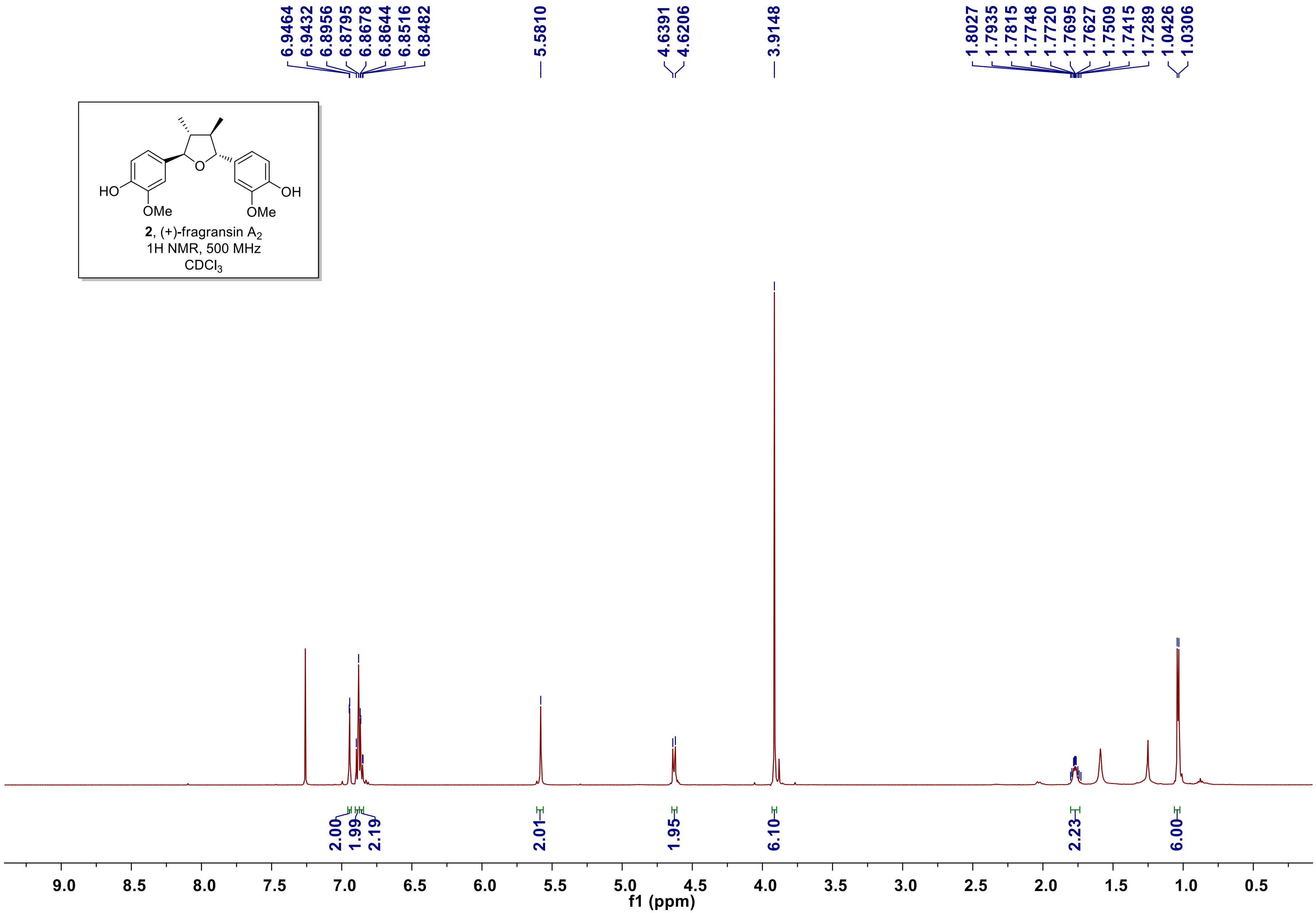
6.9464
6.9432
6.8956
6.8795
6.8678
6.8644
6.8516
6.8482
5.5810
4.6391
4.6206
3.9148
1.8027
1.7935
1.7815
1.7748
1.7720
1.7695
1.7627
1.7509
1.7415
1.7289
1.0426
1.0306
HO
OMe
O
OMe
OH
2, (+)-fragransin A2
1H NMR, 500 MHz
CDCl3
2.00
1.99
2.19
2.01
1.95
6.10
2.23
6.00
9.0
8.5
8.0
7.5
7.0
6.5
6.0
5.5
5.0
4.5
4.0
3.5
3.0
2.5
2.0
1.5
1.0
0.5
f1 (ppm)

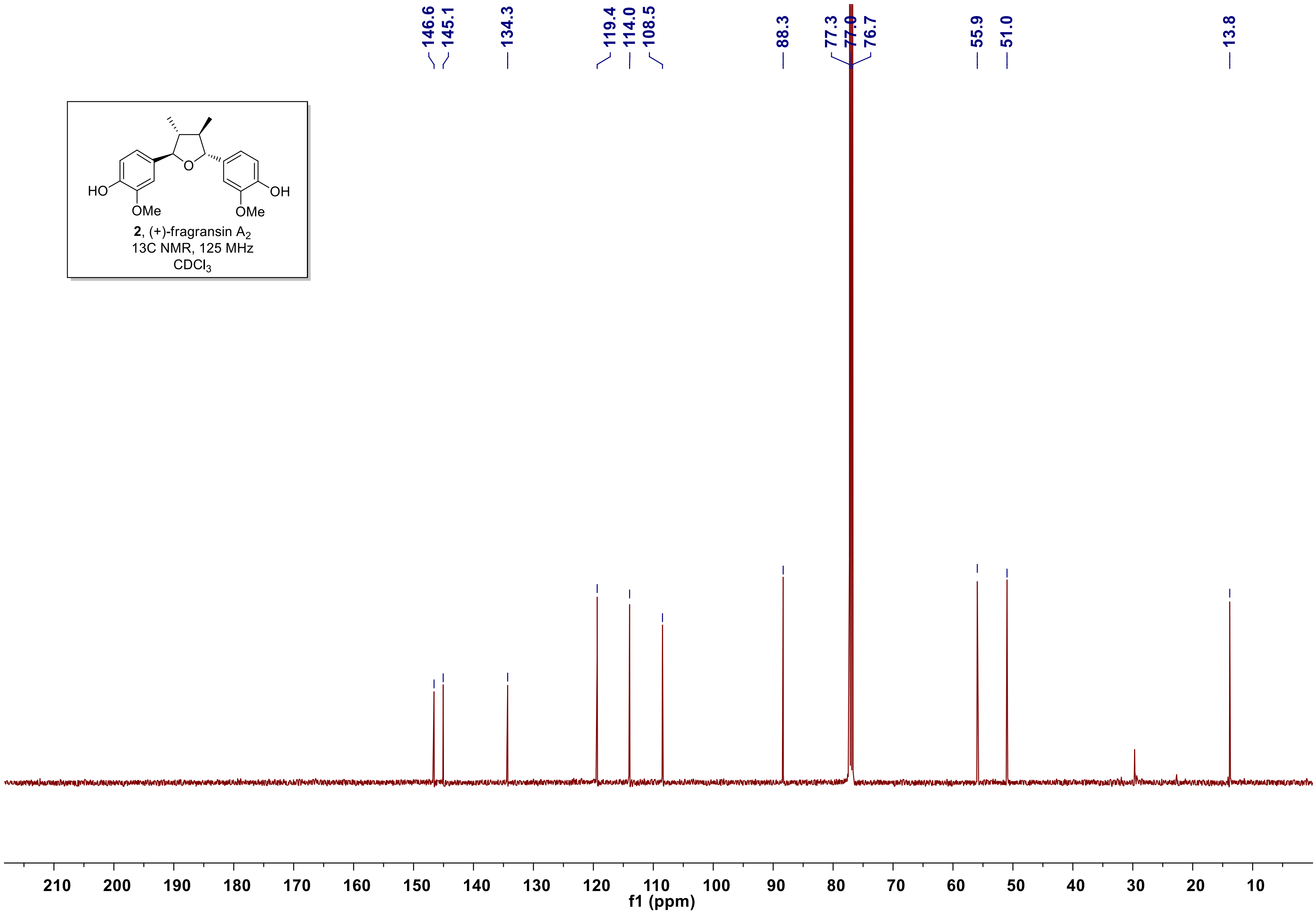

HO
OMe
O
OH
OMe
2, (+)-fragransin A2
13C NMR, 125 MHz
CDCl3
146.6
145.1
134.3
119.4
114.0
108.5
88.3
77.3
77.0
76.7
55.9
51.0
13.8
210
200
190
180
170
160
150
140
130
120
110
100
90
80
70
60
50
40
30
20
10
f1 (ppm)

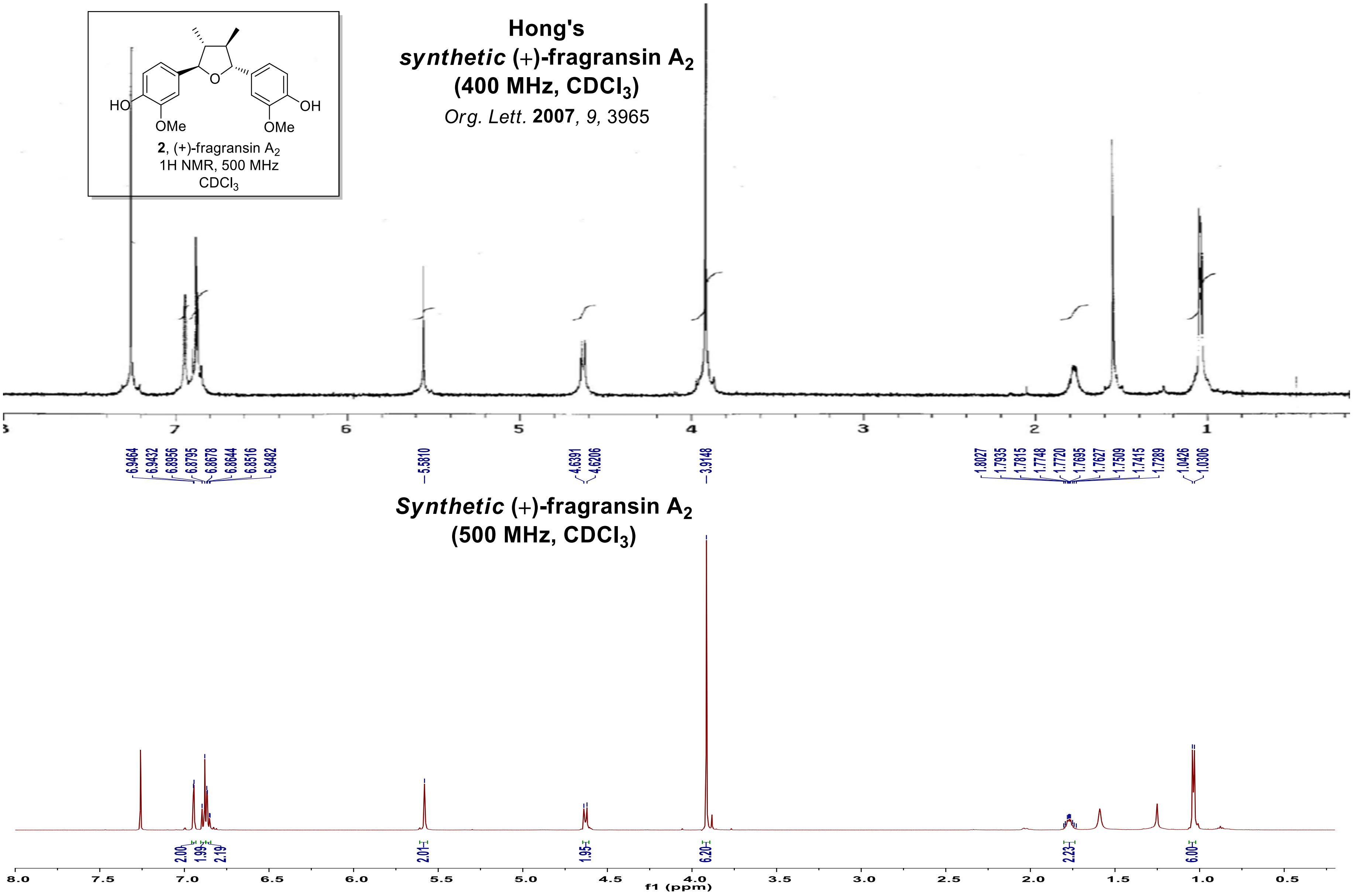
Hong's
synthetic (+)-fragransin A2
(400 MHz, CDCl3)
Org. Lett. 2007, 9, 3965
HO
OH
OMe
OMe
O
2, (+)-fragransin A2
1H NMR, 500 MHz
CDCl3
Synthetic (+)-fragransin A2
(500 MHz, CDCl3)
6.9464
6.9432
6.8956
6.8795
6.8678
6.8644
6.8516
6.8482
5.5810
4.6391
4.6206
3.9148
1.8027
1.7935
1.7815
1.7748
1.7720
1.7695
1.7627
1.7509
1.7415
1.7289
1.0426
1.0306
2.00
1.99
2.19
2.01
1.95
6.20
2.23
6.00
f1 (ppm)

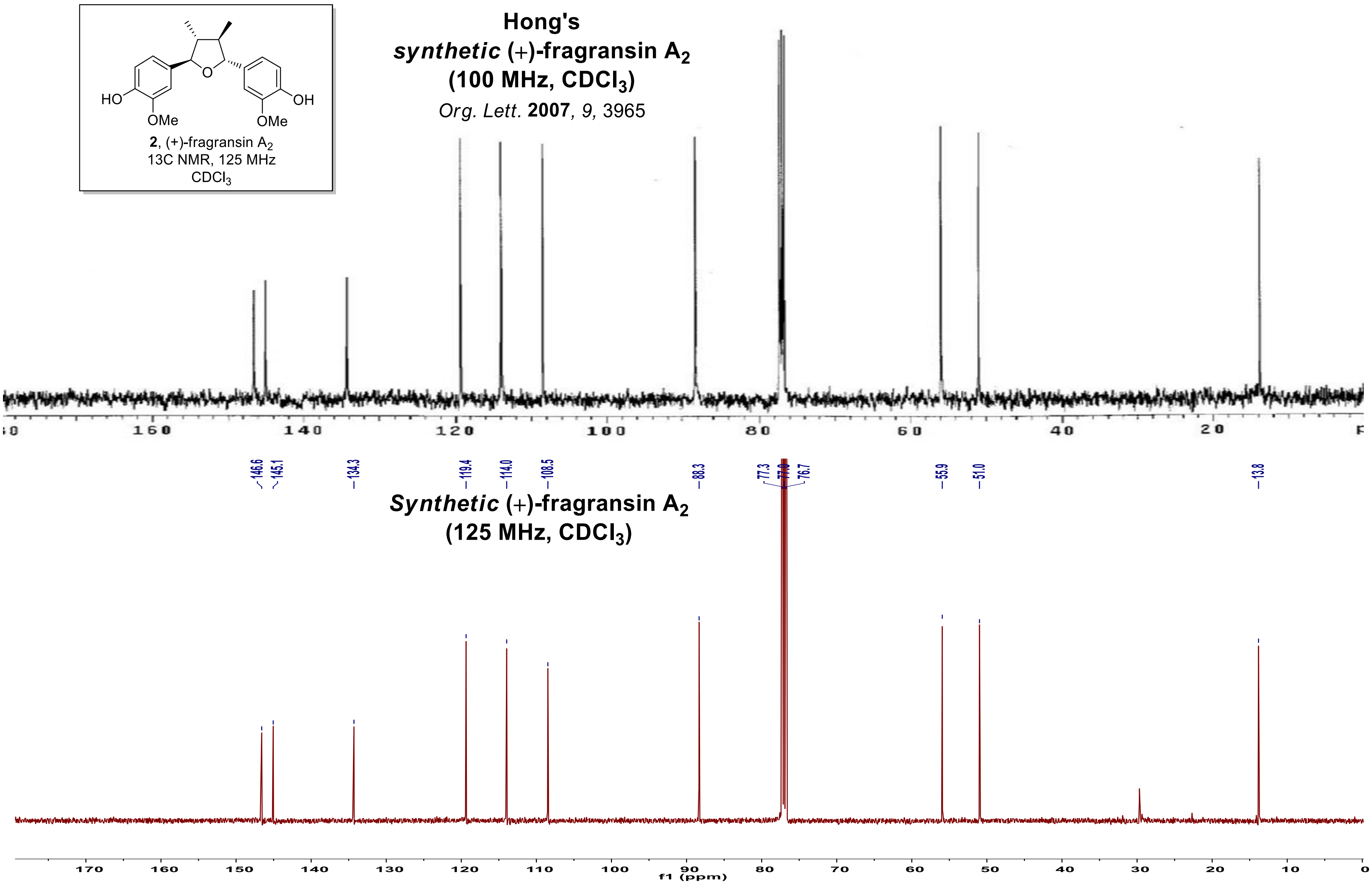
HO
OMe
OMe
OH
2, (+)-fragransin A2
13C NMR, 125 MHz
CDCl3
Hong's
synthetic (+)-fragransin A2
(100 MHz, CDCl3)
Org. Lett. 2007, 9, 3965
160
140
120
100
80
60
40
20
146.6
145.1
134.3
119.4
114.0
108.5
88.3
77.3
77.0
76.7
55.9
51.0
13.8
Synthetic (+)-fragransin A2
(125 MHz, CDCl3)
170
160
150
140
130
120
110
100
90
80
70
60
50
40
30
20
10
0
f1 (ppm)

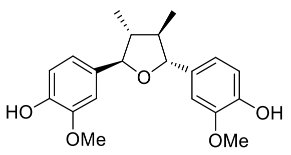

2, (+)-fragransin A_2
1H NMR, 500 MHz
$CDCl_3$

Jahn's
synthetic **(+)-fragransin A_2**
(400 MHz, $CDCl_3$)
Org. Biomol. Chem. **2018**, *16*, 750

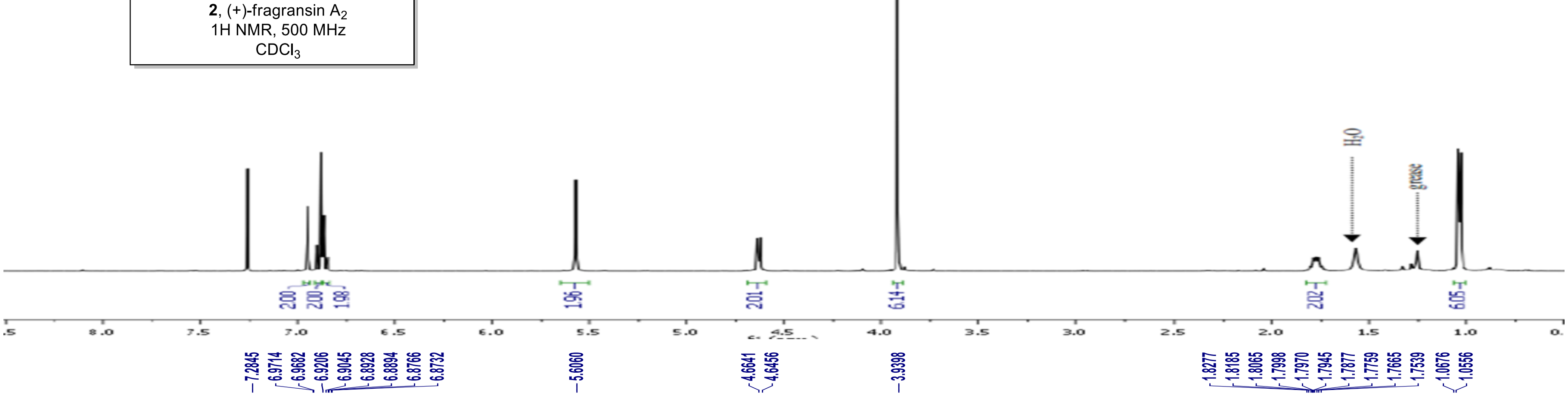

Synthetic **(+)-fragransin A_2**
(500 MHz, $CDCl_3$)

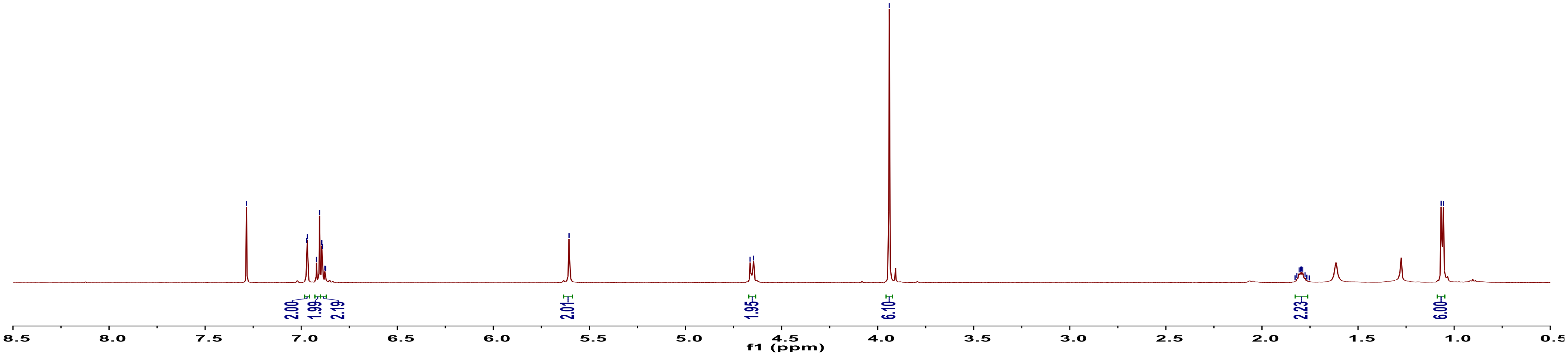

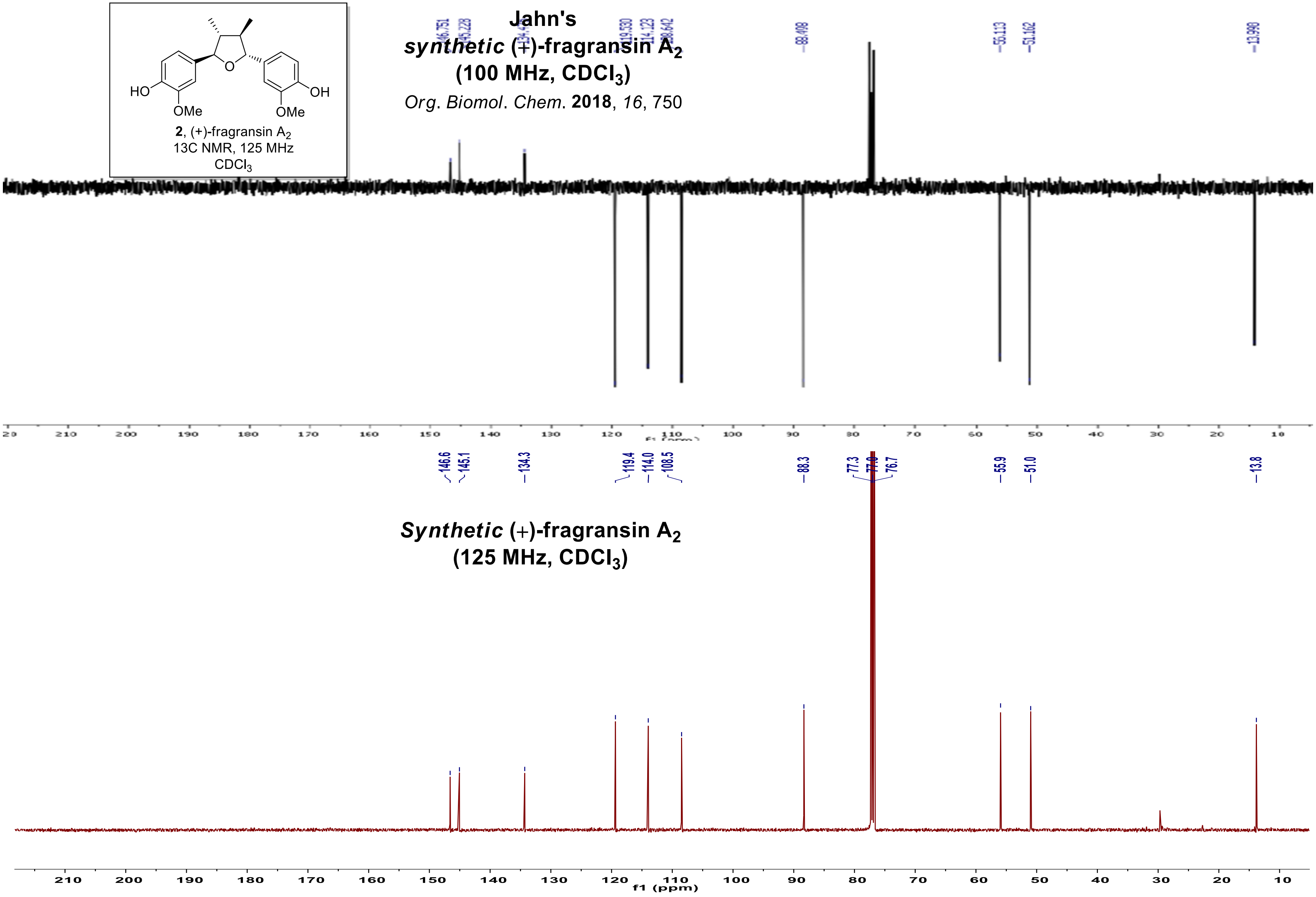
HO
OMe
OMe
OH
2, (+)-fragransin A$_2$
13C NMR, 125 MHz
CDCl$_3$
Jahn's
synthetic (+)-fragransin A$_2$
(100 MHz, CDCl$_3$)
Org. Biomol. Chem. **2018**, *16*, 750
146.751
145.228
119.530
114.123
108.642
88.408
55.133
51.162
13.990
146.6
145.1
134.3
119.4
114.0
108.5
88.3
77.3
77.0
76.7
55.9
51.0
13.8
Synthetic (+)-fragransin A$_2$
(125 MHz, CDCl$_3$)
f1 (ppm)

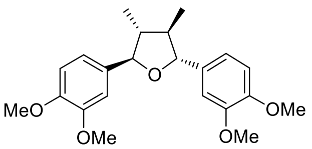

3, (+)-galbelgin
1H NMR, 500 MHz
$CDCl_3$

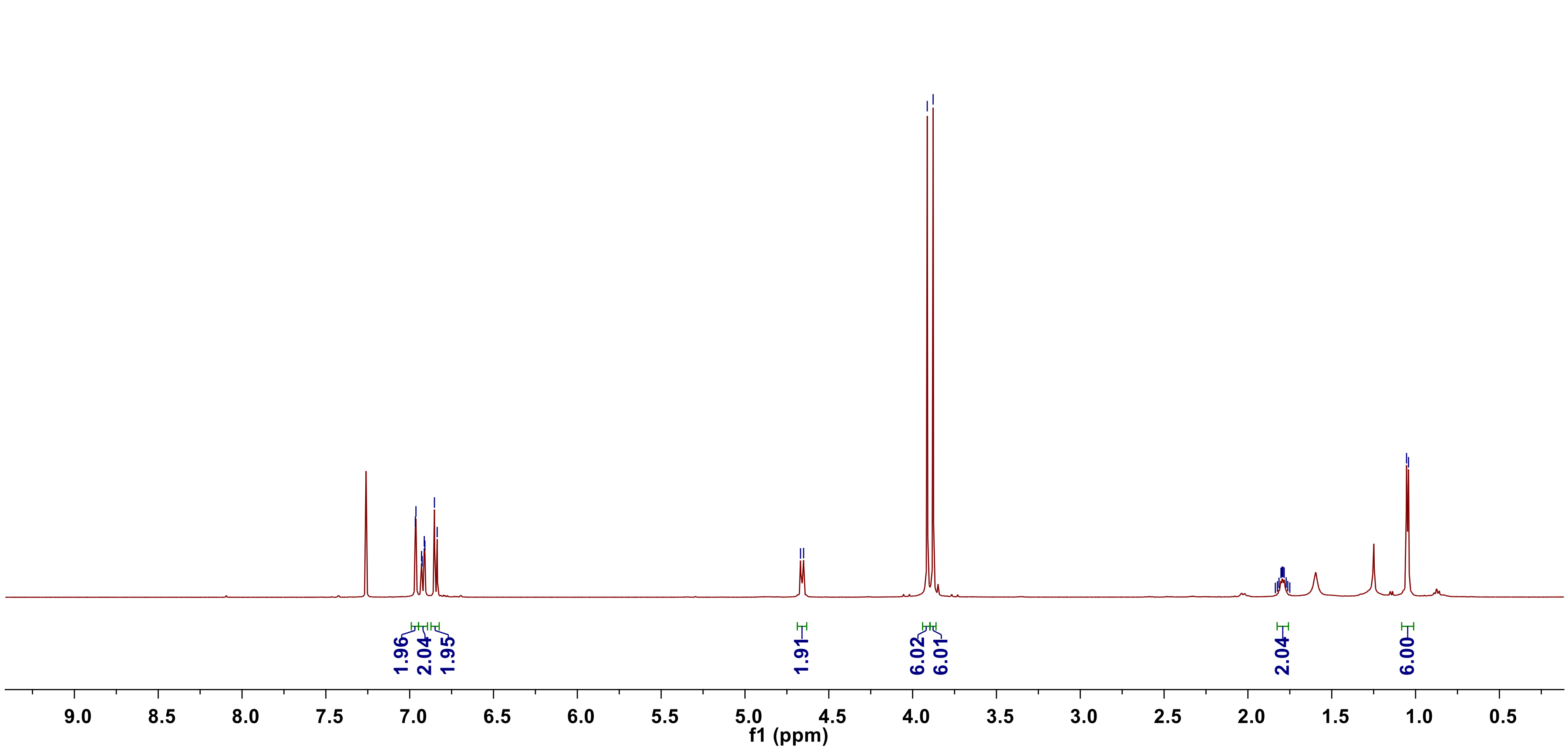

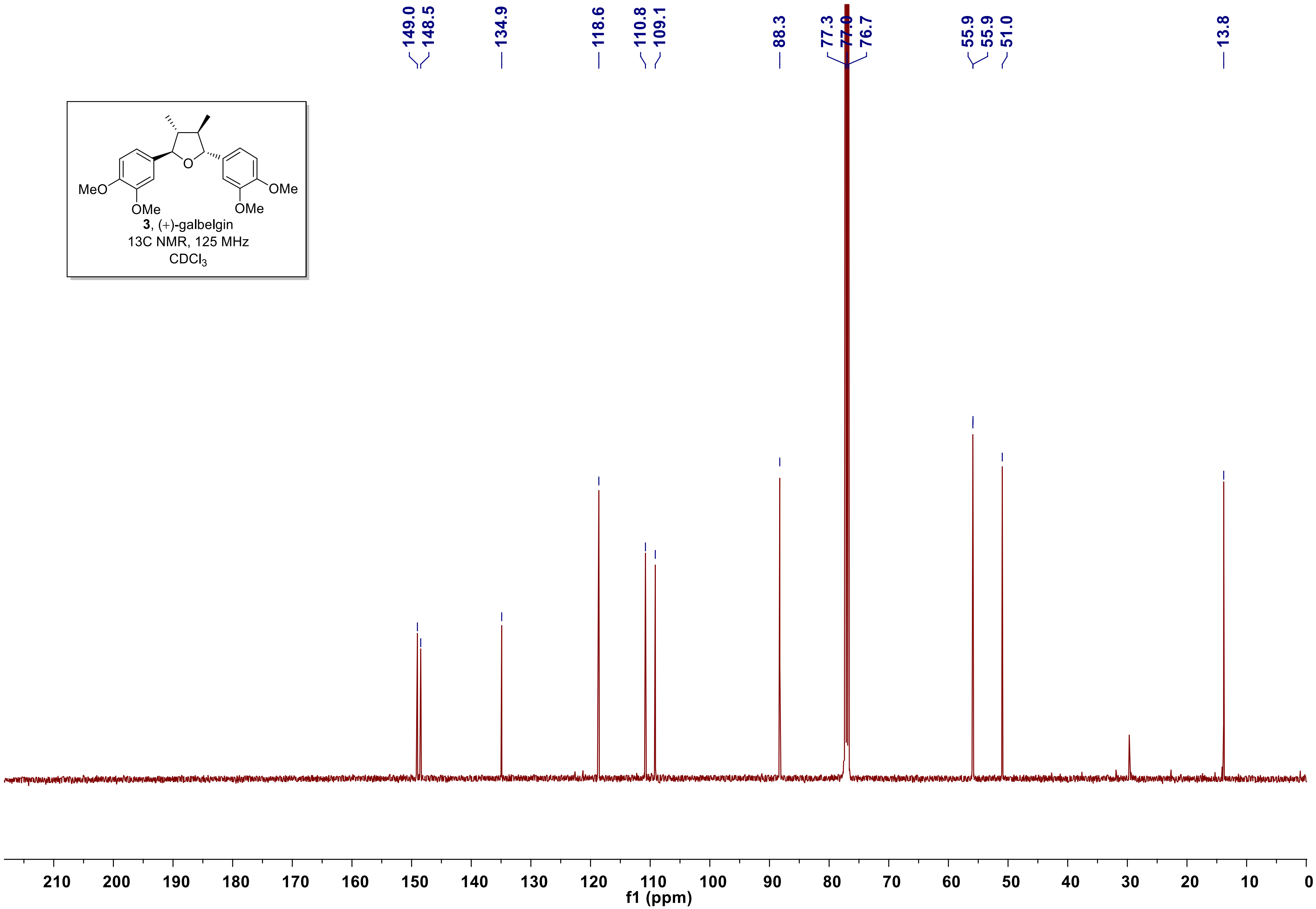
149.0
148.5
134.9
118.6
110.8
109.1
88.3
77.3
77.0
76.7
55.9
55.9
51.0
13.8
MeO
OMe
OMe
OMe
O
3, (+)-galbelgin
13C NMR, 125 MHz
CDCl3
210
200
190
180
170
160
150
140
130
120
110
100
90
80
70
60
50
40
30
20
10
0
f1 (ppm)

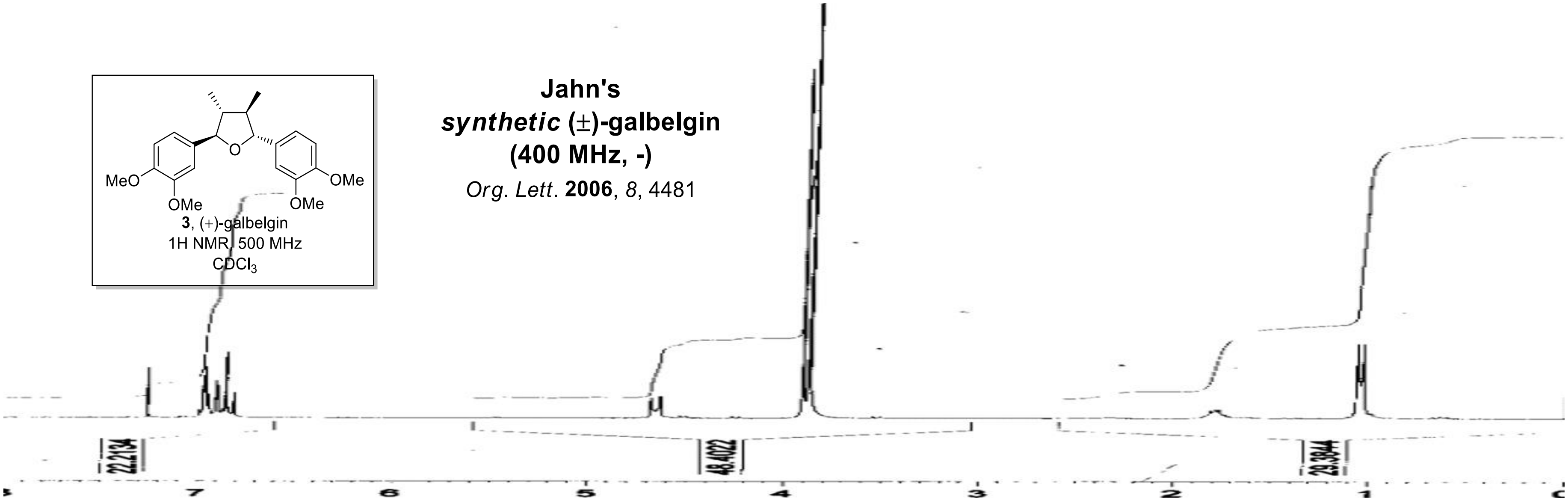
MeO
OMe
OMe
OMe
3, (+)-galbelgin
1H NMR, 500 MHz
CDCl3
Jahn's
synthetic (±)-galbelgin
(400 MHz, -)
Org. Lett. 2006, 8, 4481
22.2134
48.4022
29.3844

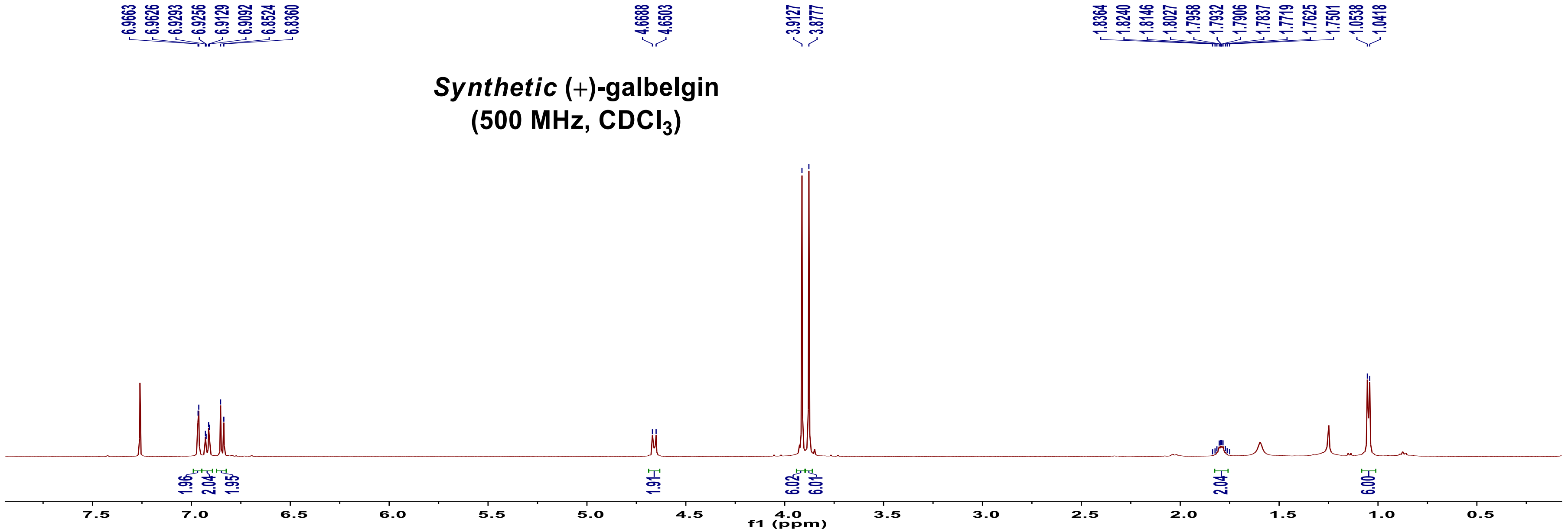
6.9663
6.9626
6.9293
6.9256
6.9129
6.9092
6.8524
6.8360
4.6688
4.6503
3.9127
3.8777
1.8364
1.8240
1.8146
1.8027
1.7958
1.7932
1.7906
1.7837
1.7719
1.7625
1.7501
1.0538
1.0418
Synthetic (+)-galbelgin
(500 MHz, CDCl3)
1.96
2.04
1.95
1.91
6.02
6.01
2.04
6.00
f1 (ppm)

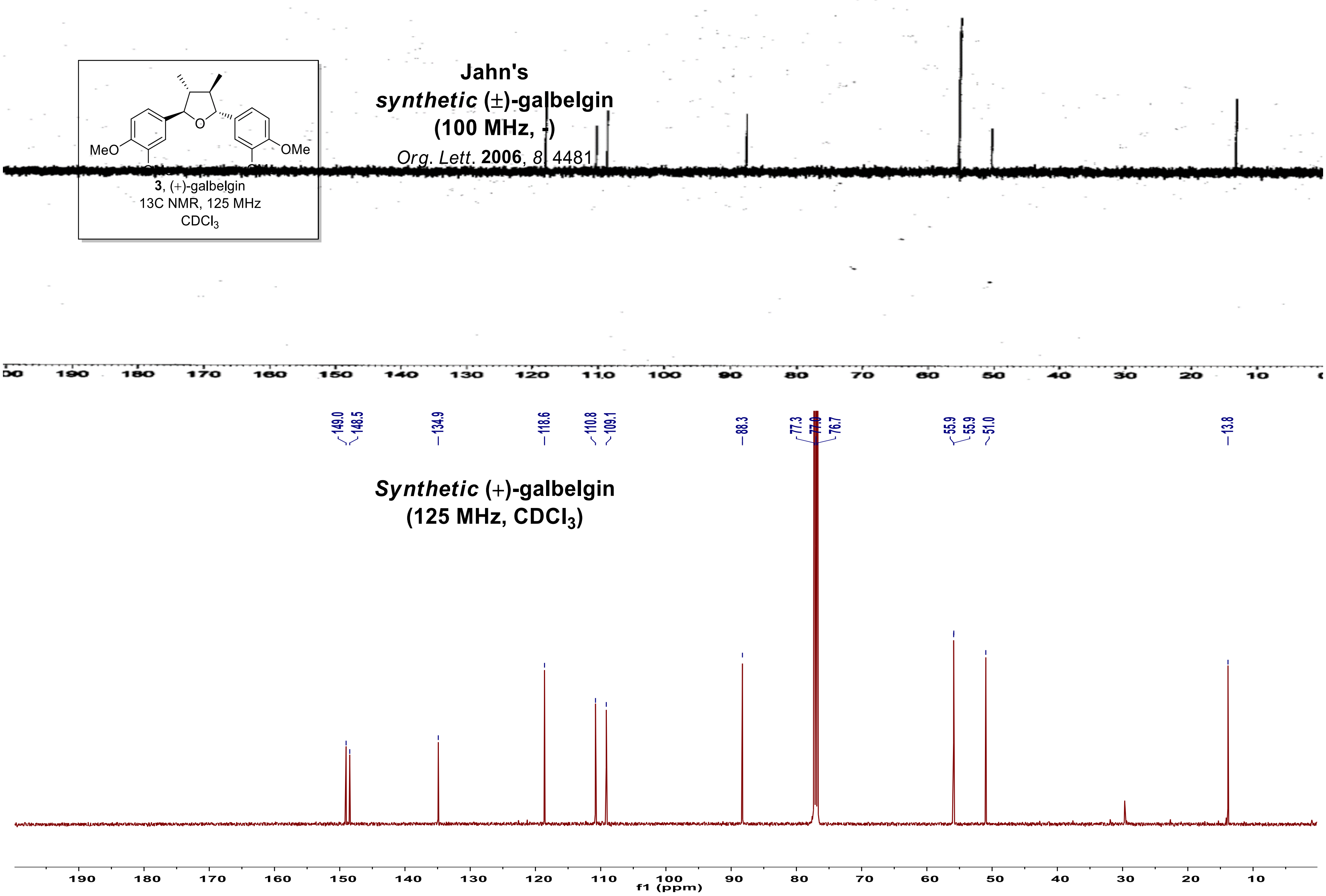
Jahn's
synthetic (±)-galbelgin
(100 MHz, -)
Org. Lett. 2006, 8, 4481
MeO
OMe
3, (+)-galbelgin
13C NMR, 125 MHz
CDCl3
149.0
148.5
134.9
118.6
110.8
109.1
88.3
77.3
77.0
76.7
55.9
55.9
51.0
13.8
Synthetic (+)-galbelgin
(125 MHz, CDCl3)
f1 (ppm)

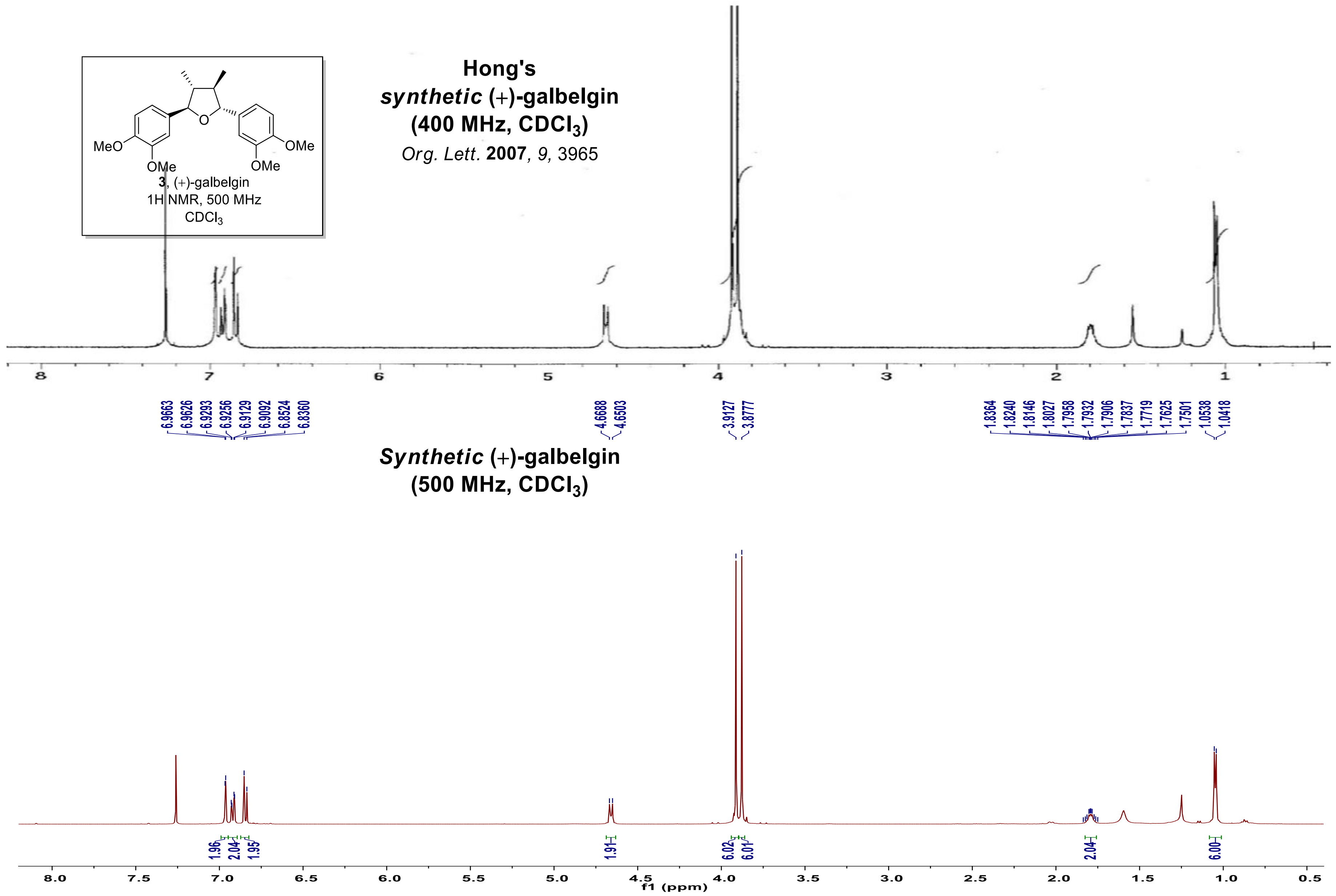

MeO
OMe
OMe
OMe
O
3, (+)-galbelgin
1H NMR, 500 MHz
CDCl3
Hong's
synthetic (+)-galbelgin
(400 MHz, CDCl3)
Org. Lett. 2007, 9, 3965
8
7
6
5
4
3
2
1
6.9663
6.9626
6.9293
6.9256
6.9129
6.9092
6.8524
6.8360
4.6688
4.6503
3.9127
3.8777
1.8364
1.8240
1.8146
1.8027
1.7958
1.7932
1.7906
1.7837
1.7719
1.7625
1.7501
1.0538
1.0418
Synthetic (+)-galbelgin
(500 MHz, CDCl3)
1.96
2.04
1.95
1.91
6.02
6.01
2.04
6.00
8.0
7.5
7.0
6.5
6.0
5.5
5.0
4.5
4.0
3.5
3.0
2.5
2.0
1.5
1.0
0.5
f1 (ppm)

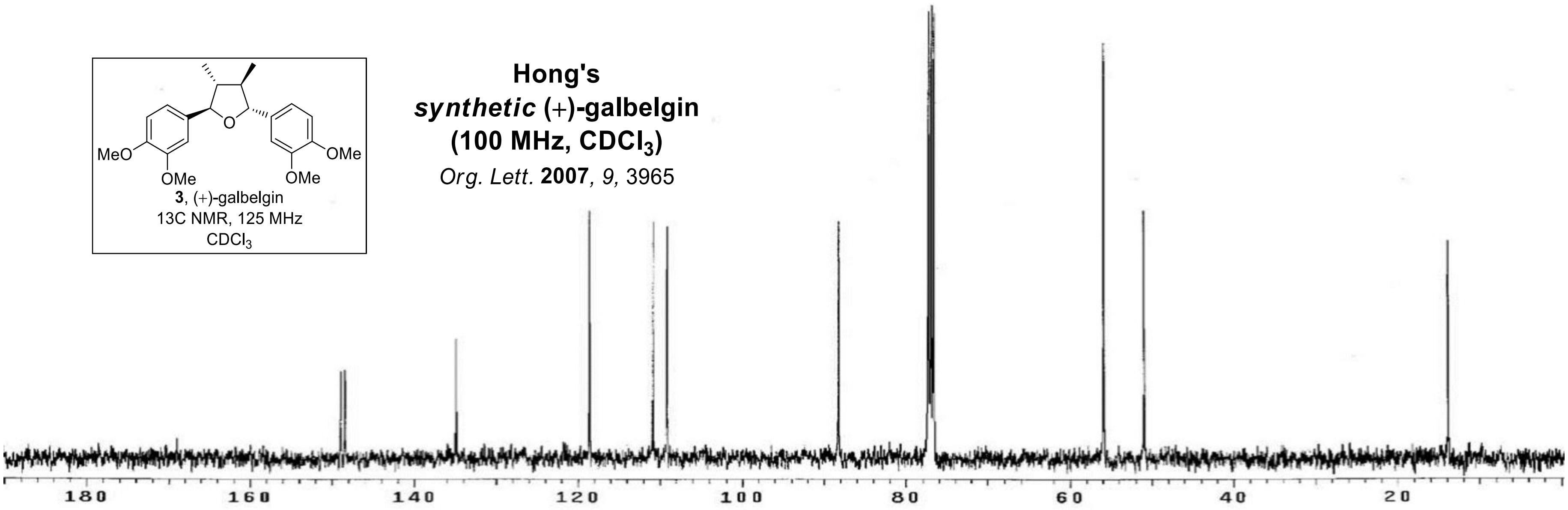

3, (+)-galbelgin
13C NMR, 125 MHz
$CDCl_3$

Hong's
***synthetic* (+)-galbelgin**
(100 MHz, $CDCl_3$)
Org. Lett. **2007**, *9,* 3965

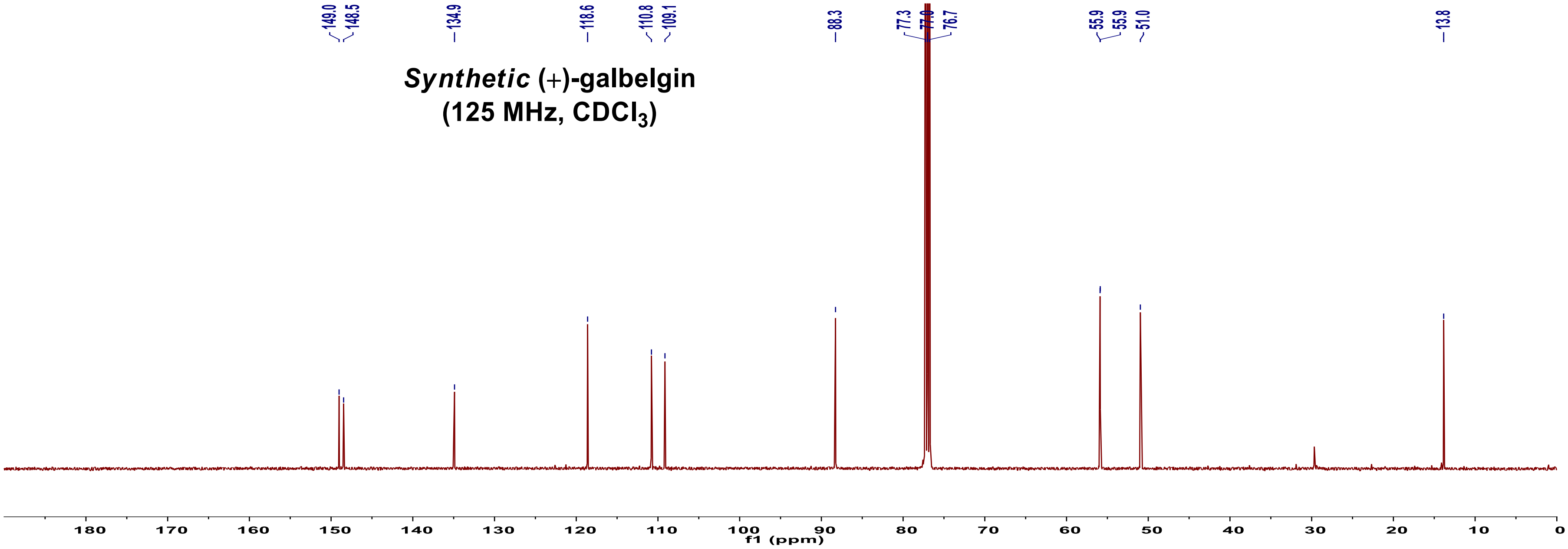

***Synthetic* (+)-galbelgin**
(125 MHz, $CDCl_3$)

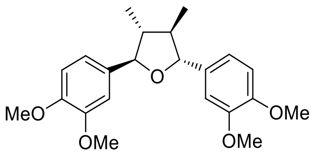

3, (+)-galbelgin
1H NMR, 500 MHz
$CDCl_3$

Rye's
***synthetic* (+)-galbelgin**
(400 MHz, $CDCl_3$)
J. Org. Chem. **2011**, *76*, 6636

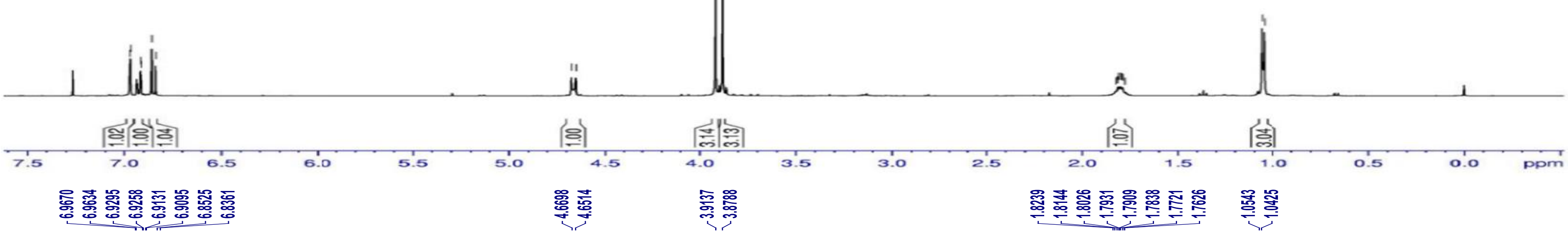

***Synthetic* (+)-galbelgin**
(500 MHz, $CDCl_3$)

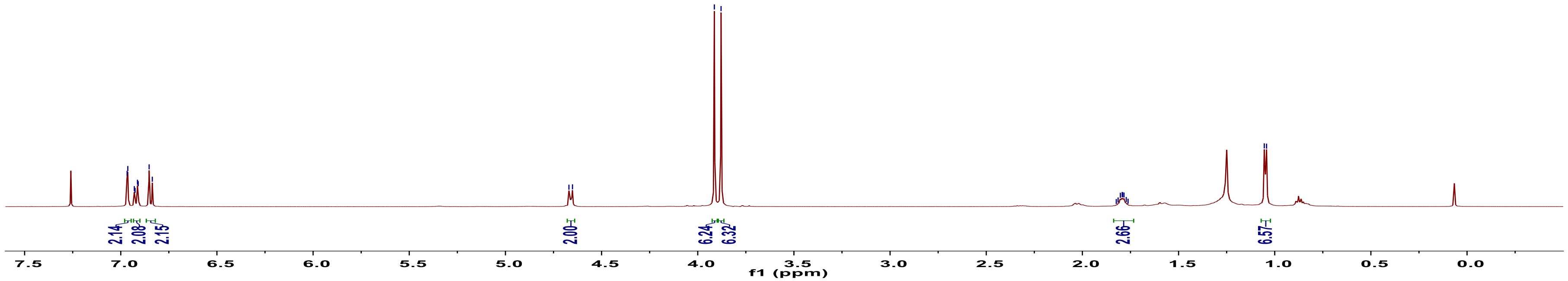

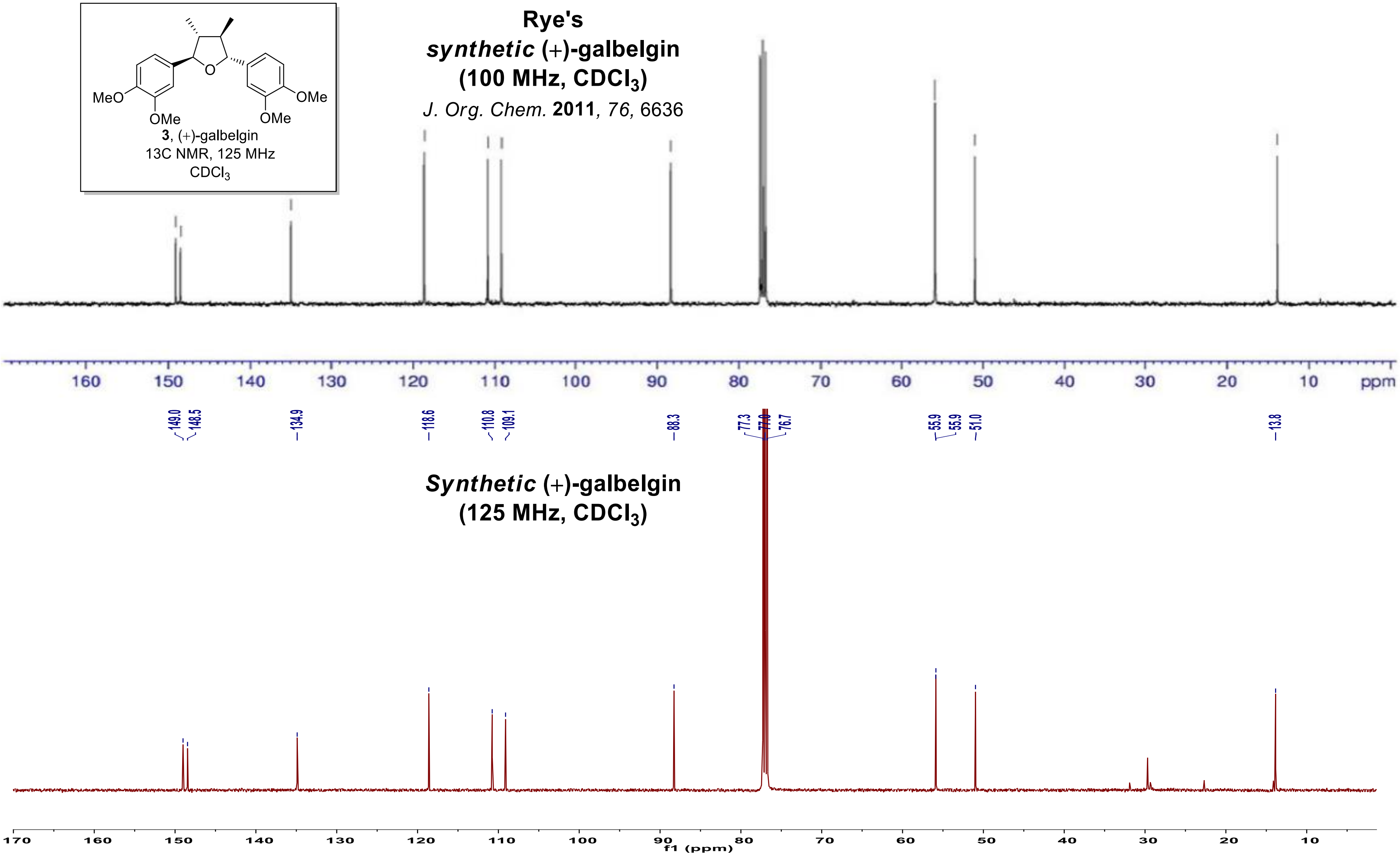
MeO
OMe
OMe
OMe
3, (+)-galbelgin
13C NMR, 125 MHz
CDCl3
Rye's
synthetic (+)-galbelgin
(100 MHz, CDCl3)
J. Org. Chem. 2011, 76, 6636
160 150 140 130 120 110 100 90 80 70 60 50 40 30 20 10 ppm
149.0
148.5
134.9
118.6
110.8
109.1
88.3
77.3
77.0
76.7
55.9
55.9
51.0
13.8
Synthetic (+)-galbelgin
(125 MHz, CDCl3)
170 160 150 140 130 120 110 100 90 80 70 60 50 40 30 20 10
f1 (ppm)

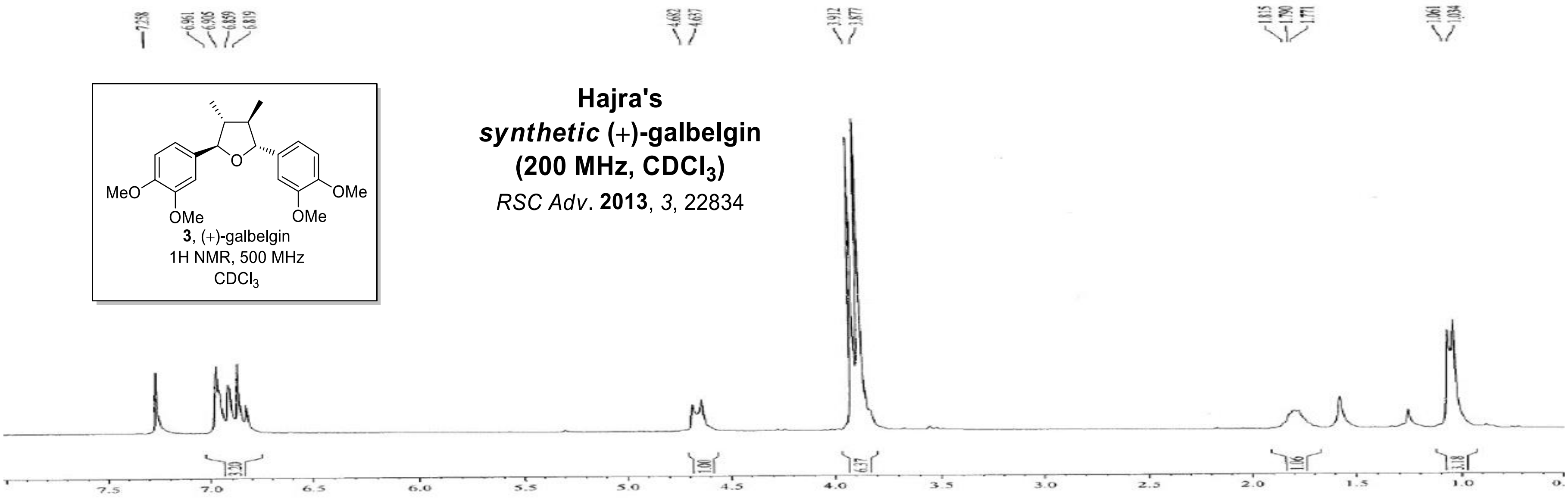

3, (+)-galbelgin
1H NMR, 500 MHz
$CDCl_3$

Hajra's
***synthetic* (+)-galbelgin**
(200 MHz, $CDCl_3$)
RSC Adv. **2013**, *3*, 22834

***Synthetic* (+)-galbelgin**
(500 MHz, $CDCl_3$)

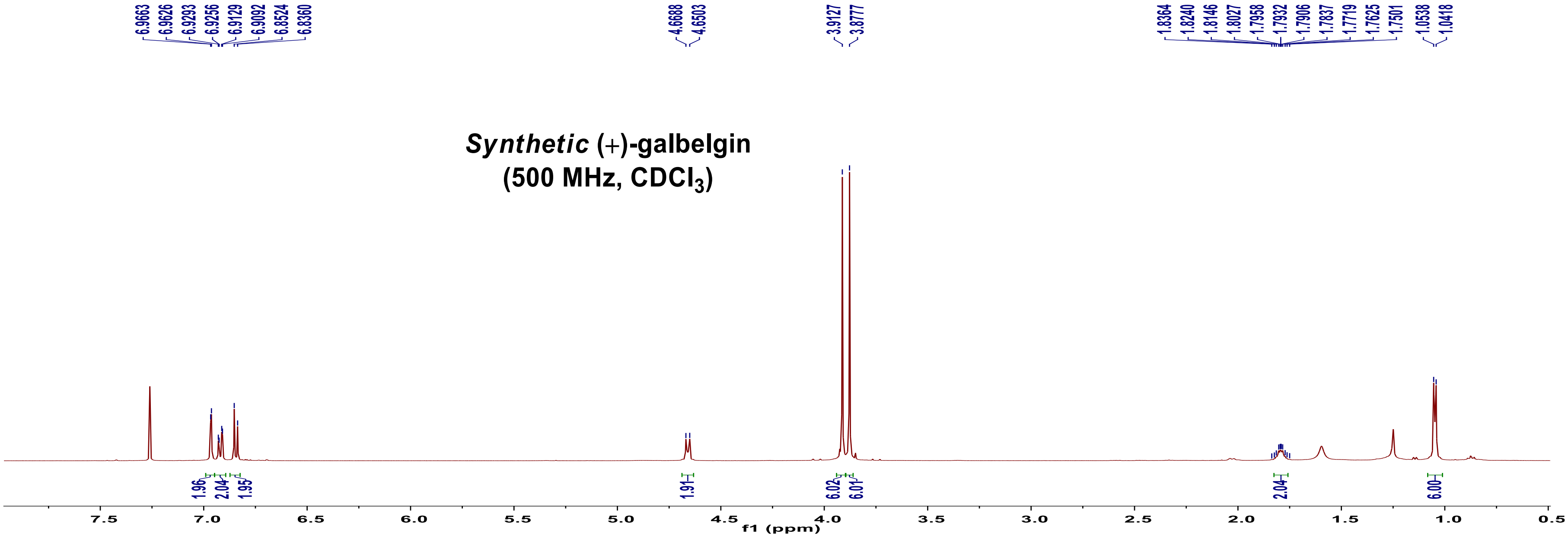

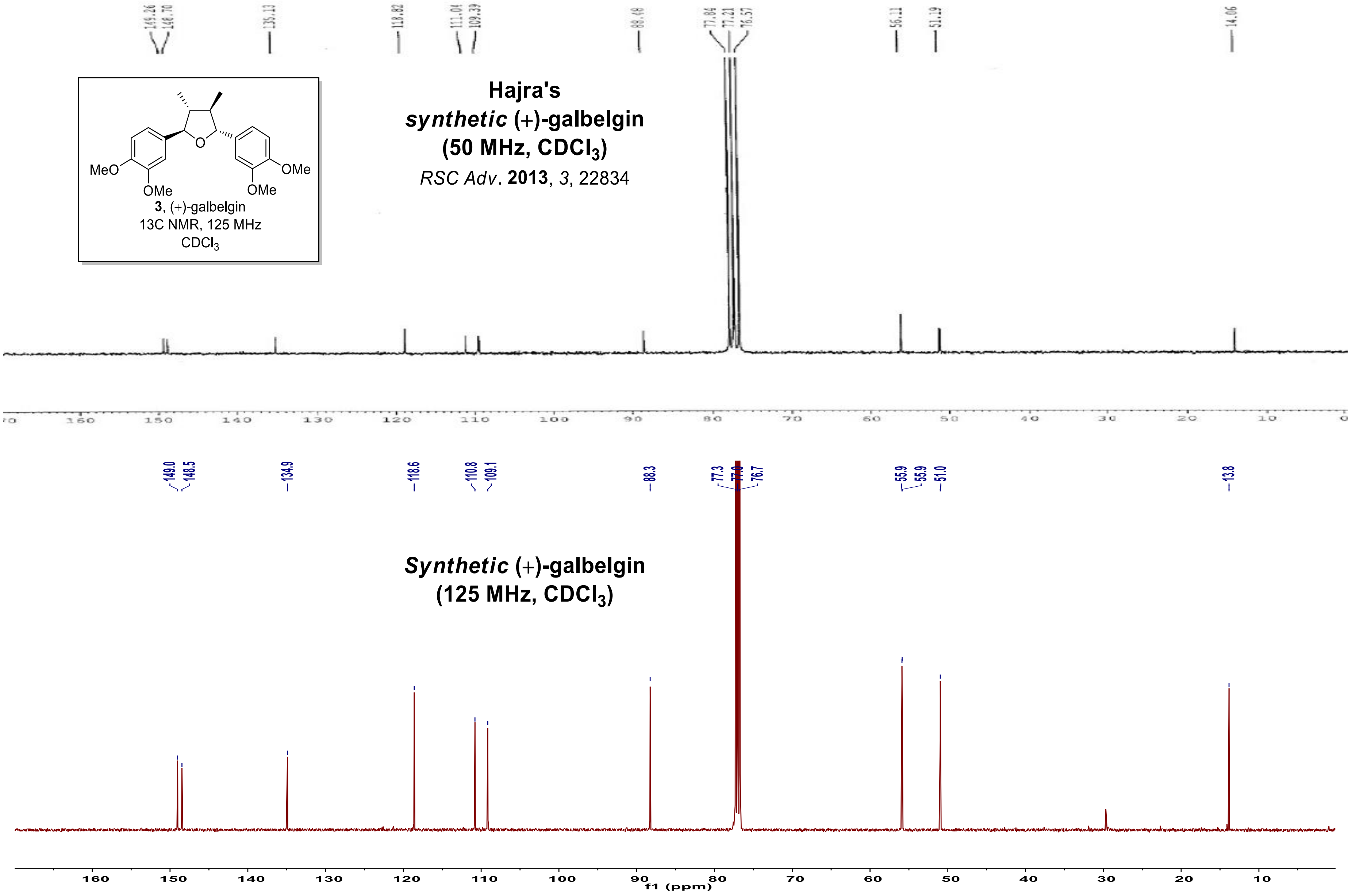

149.26
148.70
135.13
118.82
111.04
109.39
88.48
77.84
77.21
76.57
56.11
51.19
14.06
Hajra's
synthetic (+)-galbelgin
(50 MHz, CDCl3)
RSC Adv. 2013, 3, 22834
MeO
OMe
OMe
OMe
O
3, (+)-galbelgin
13C NMR, 125 MHz
CDCl3
149.0
148.5
134.9
118.6
110.8
109.1
88.3
77.3
76.7
55.9
55.9
51.0
13.8
Synthetic (+)-galbelgin
(125 MHz, CDCl3)
f1 (ppm)

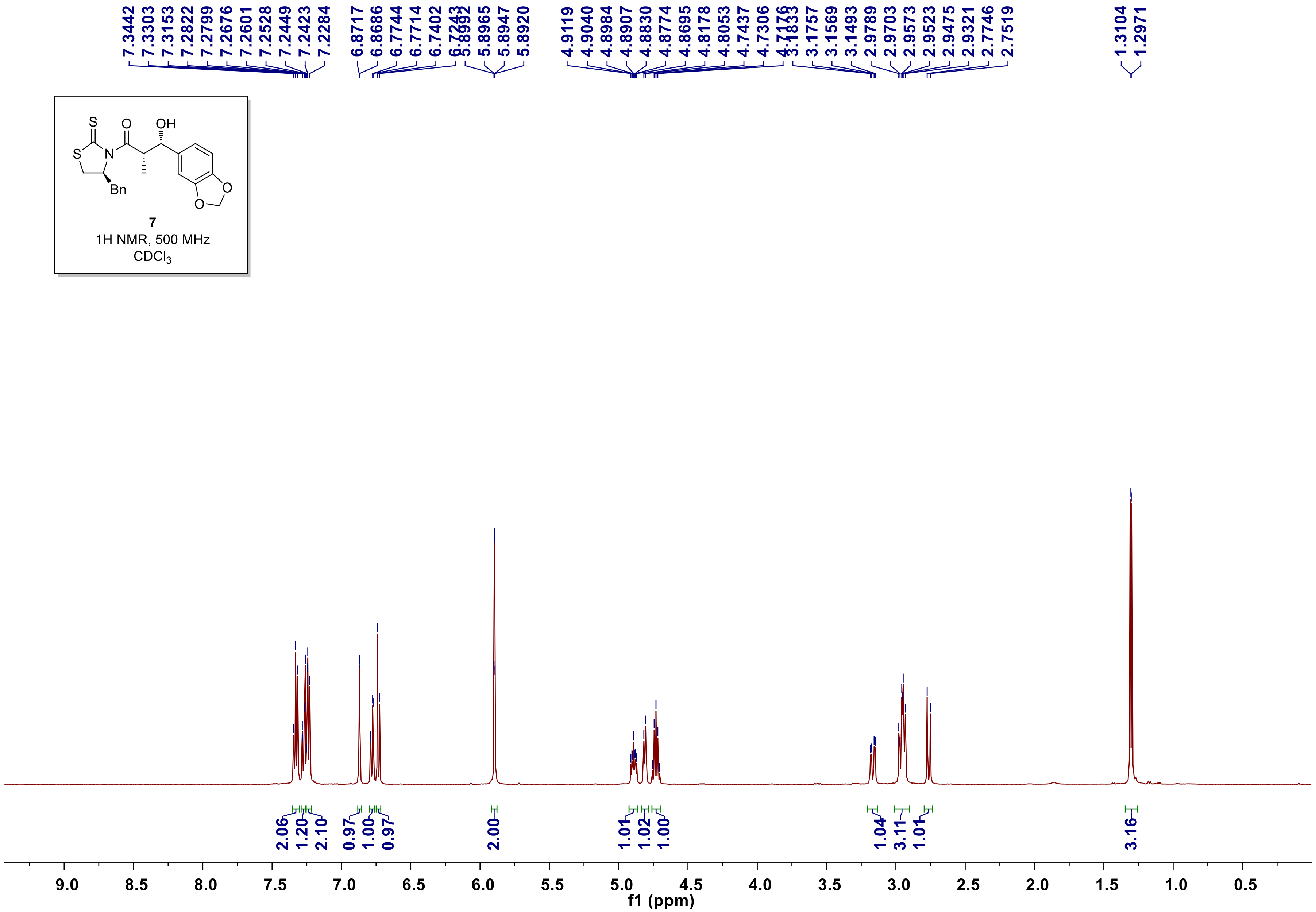
7.3442
7.3303
7.3153
7.2822
7.2799
7.2676
7.2601
7.2528
7.2449
7.2423
7.2284
6.8717
6.8686
6.7744
6.7714
6.7402
6.7243
5.8992
5.8965
5.8947
5.8920
4.9119
4.9040
4.8984
4.8907
4.8830
4.8774
4.8695
4.8178
4.8053
4.7437
4.7306
4.7176
3.1833
3.1757
3.1569
3.1493
2.9789
2.9703
2.9573
2.9523
2.9475
2.9321
2.7746
2.7519
1.3104
1.2971
S
O
OH
S
N
Bn
O
O
7
1H NMR, 500 MHz
CDCl3
2.06
1.20
2.10
0.97
1.00
0.97
2.00
1.01
1.02
1.00
1.04
3.11
1.01
3.16
9.0
8.5
8.0
7.5
7.0
6.5
6.0
5.5
5.0
4.5
4.0
3.5
3.0
2.5
2.0
1.5
1.0
0.5
f1 (ppm)

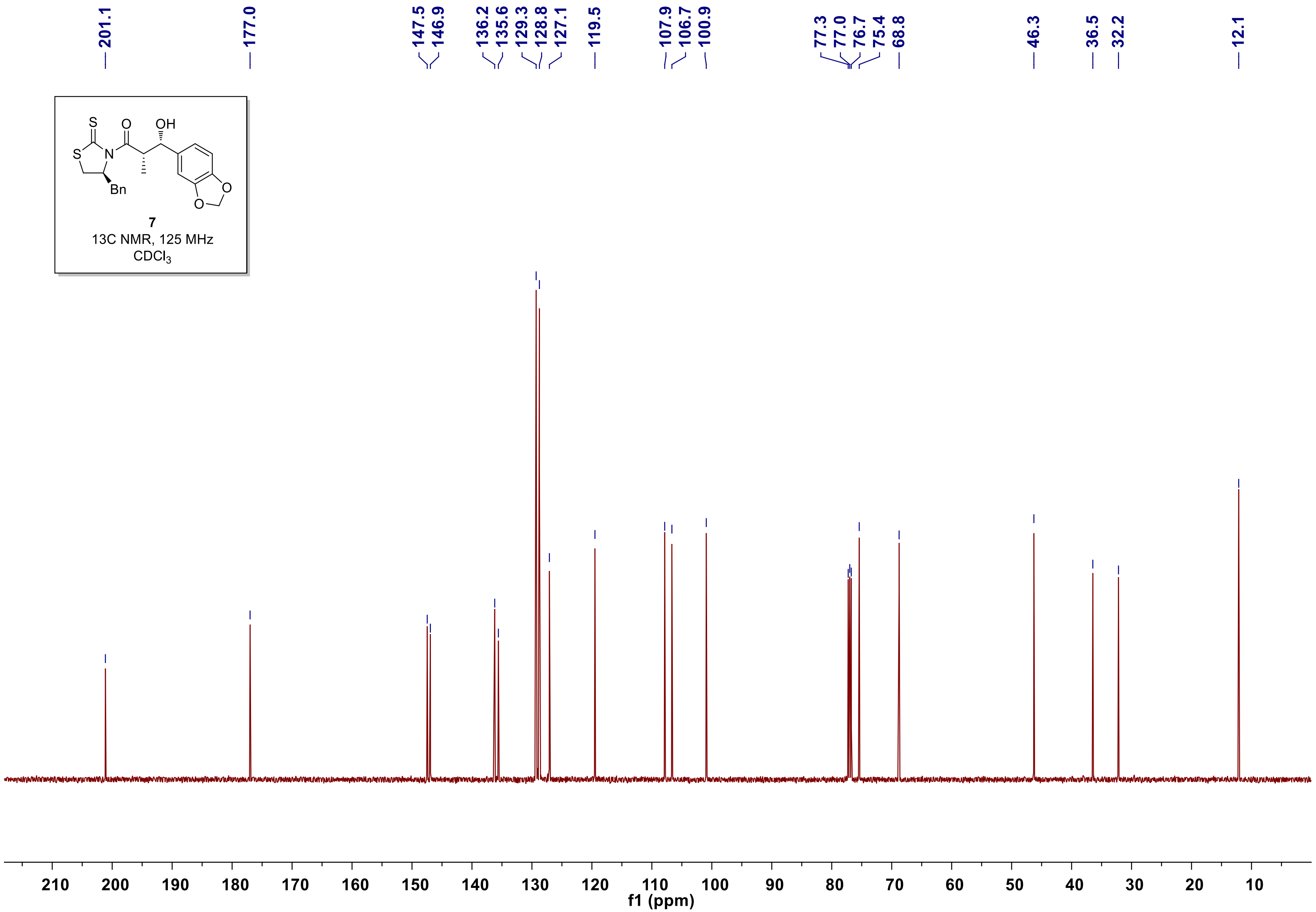
201.1
177.0
147.5
146.9
136.2
135.6
129.3
128.8
127.1
119.5
107.9
106.7
100.9
77.3
77.0
76.7
75.4
68.8
46.3
36.5
32.2
12.1
7
13C NMR, 125 MHz
CDCl3
f1 (ppm)

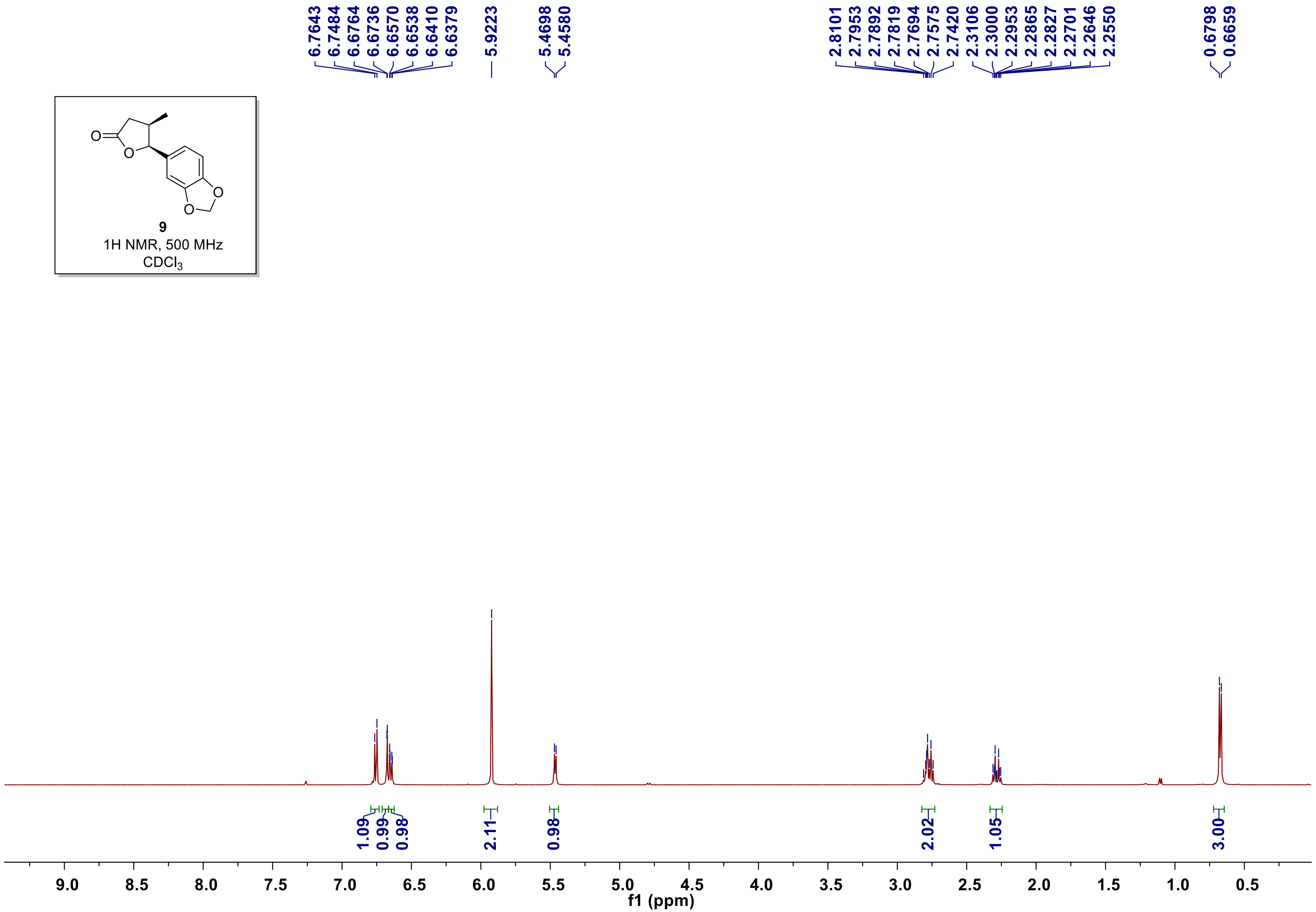
6.7643
6.7484
6.6764
6.6736
6.6570
6.6538
6.6410
6.6379
5.9223
5.4698
5.4580
2.8101
2.7953
2.7892
2.7819
2.7694
2.7575
2.7420
2.3106
2.3000
2.2953
2.2865
2.2827
2.2701
2.2646
2.2550
0.6798
0.6659
9
1H NMR, 500 MHz
$CDCl_3$
1.09
0.99
0.98
2.11
0.98
2.02
1.05
3.00
9.0
8.5
8.0
7.5
7.0
6.5
6.0
5.5
5.0
4.5
4.0
3.5
3.0
2.5
2.0
1.5
1.0
0.5
f1 (ppm)

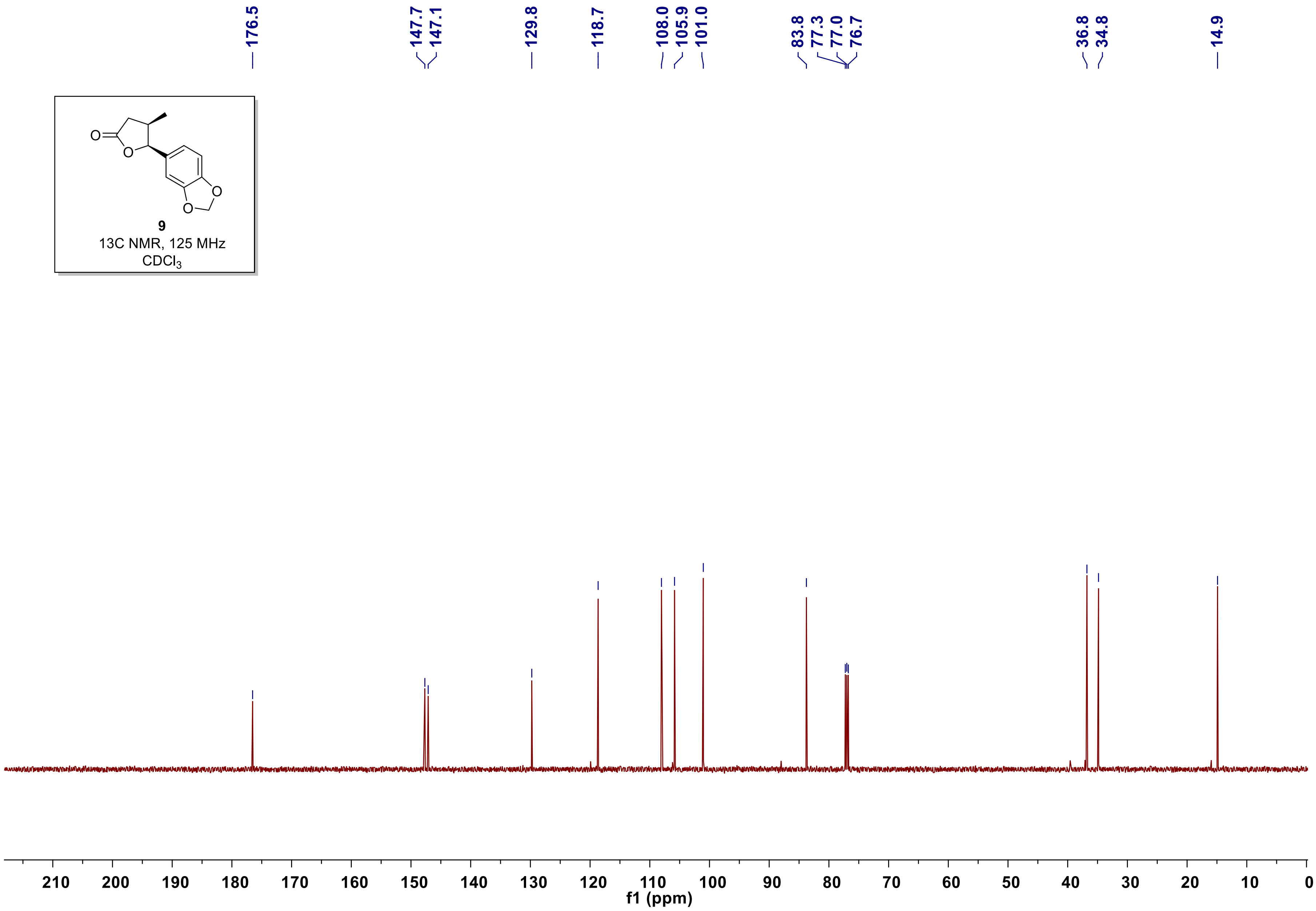
176.5
147.7
147.1
129.8
118.7
108.0
105.9
101.0
83.8
77.3
77.0
76.7
36.8
34.8
14.9
9
13C NMR, 125 MHz
$CDCl_3$
210 200 190 180 170 160 150 140 130 120 110 100 90 80 70 60 50 40 30 20 10 0
f1 (ppm)

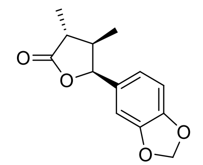

9A

1H NMR, 500 MHz

$CDCl_3$

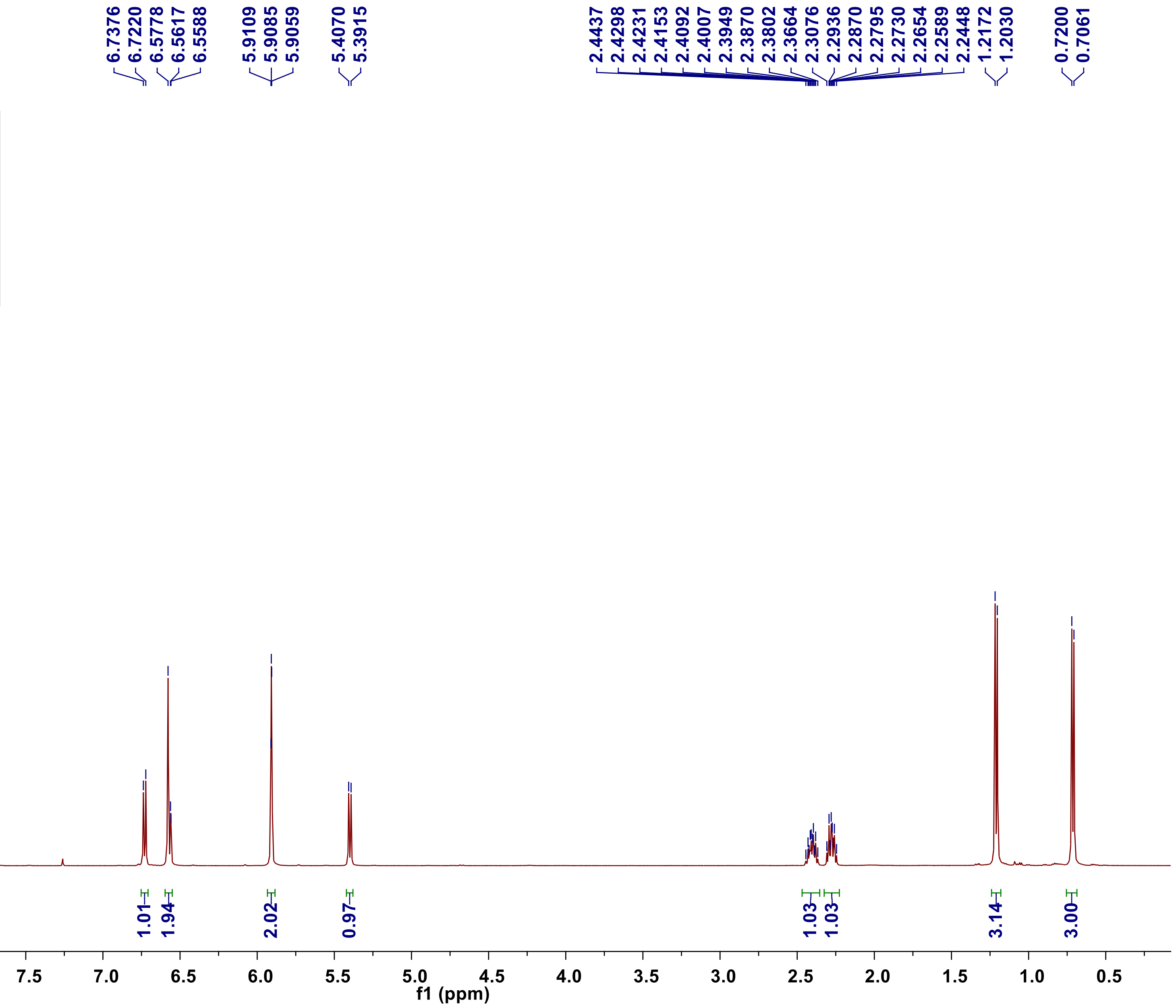

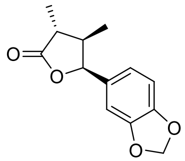

9A
13C NMR, 125 MHz
$CDCl_3$

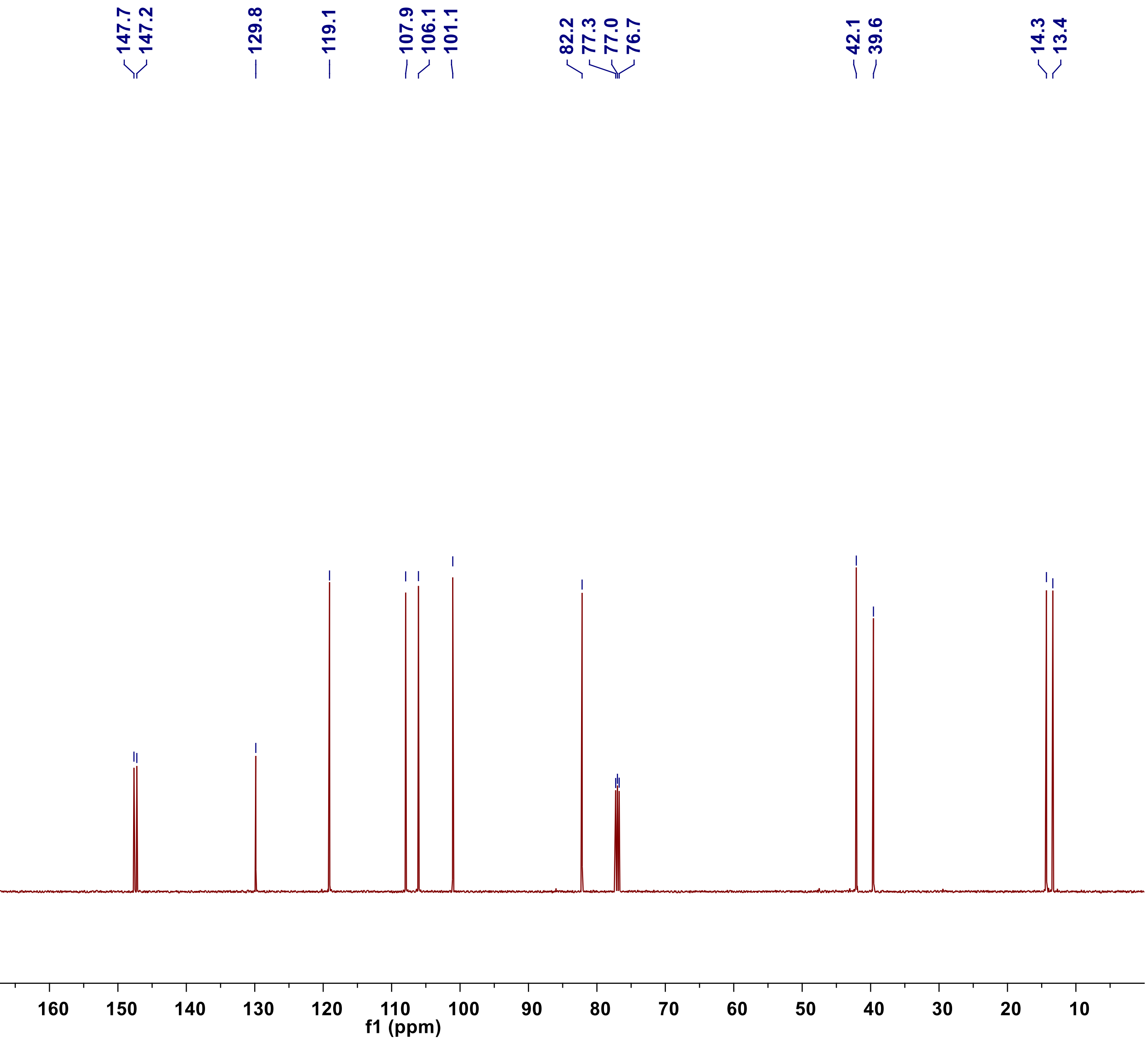

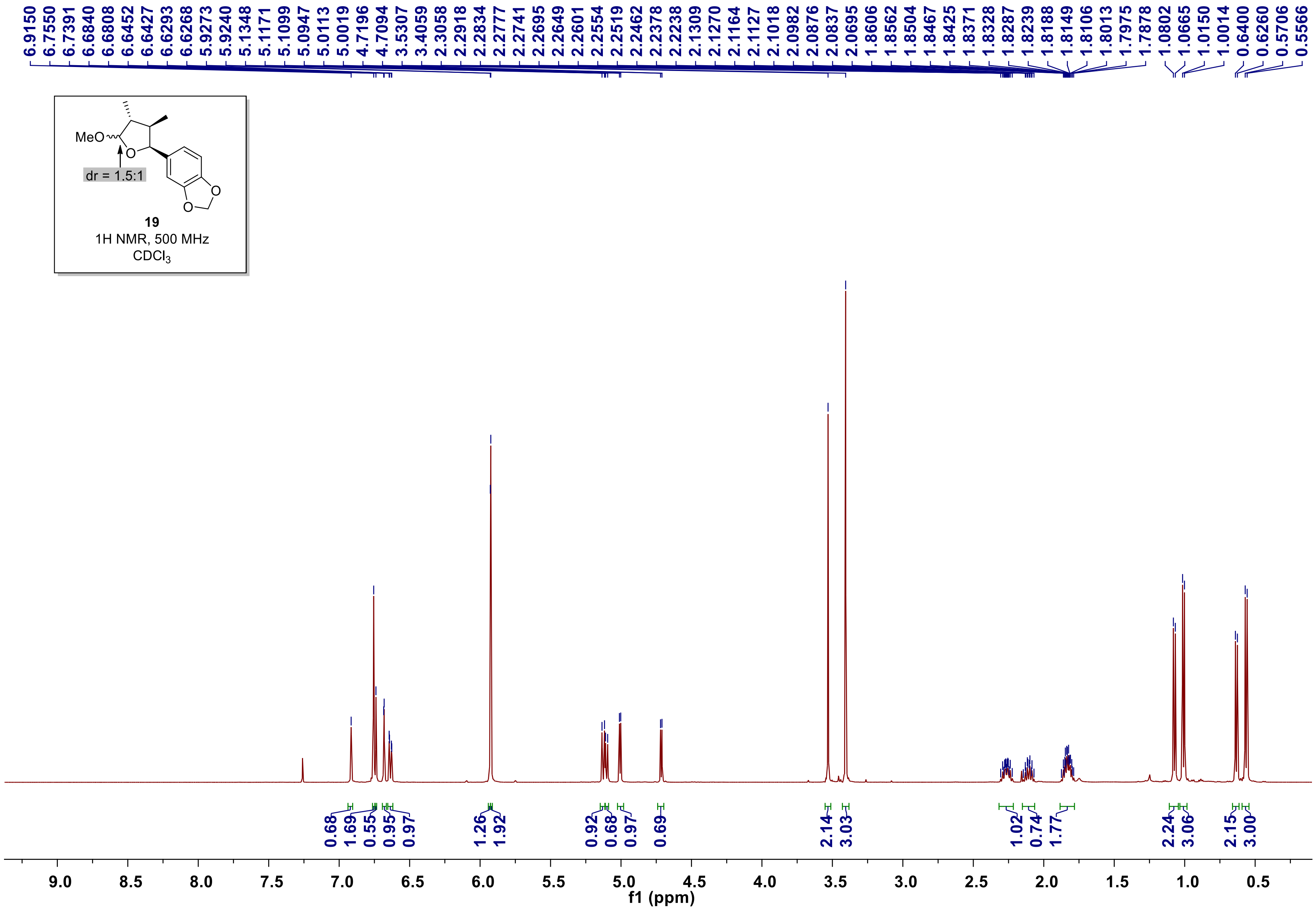
6.9150 6.7550 6.7391 6.6840 6.6808 6.6452 6.6427 6.6293 6.6268 5.9273 5.9240 5.1348 5.1171 5.1099 5.0947 5.0113 5.0019 4.7196 4.7094 3.5307 3.4059 2.3058 2.2918 2.2834 2.2777 2.2741 2.2695 2.2649 2.2601 2.2554 2.2519 2.2462 2.2378 2.2238 2.1309 2.1270 2.1164 2.1127 2.1018 2.0982 2.0876 2.0837 2.0695 1.8606 1.8562 1.8504 1.8467 1.8425 1.8371 1.8328 1.8287 1.8239 1.8188 1.8149 1.8106 1.8013 1.7975 1.7878 1.0802 1.0665 1.0150 1.0014 0.6400 0.6260 0.5706 0.5566
MeO
O
O
O
dr = 1.5:1
19
1H NMR, 500 MHz
CDCl3
0.68 1.69 0.55 0.95 0.97 1.26 1.92 0.92 0.68 0.97 0.69 2.14 3.03 1.02 0.74 1.77 2.24 3.06 2.15 3.00
9.0 8.5 8.0 7.5 7.0 6.5 6.0 5.5 5.0 4.5 4.0 3.5 3.0 2.5 2.0 1.5 1.0 0.5
f1 (ppm)

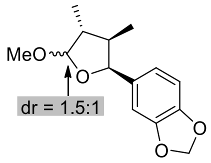

19
13C NMR, 125 MHz
$CDCl_3$

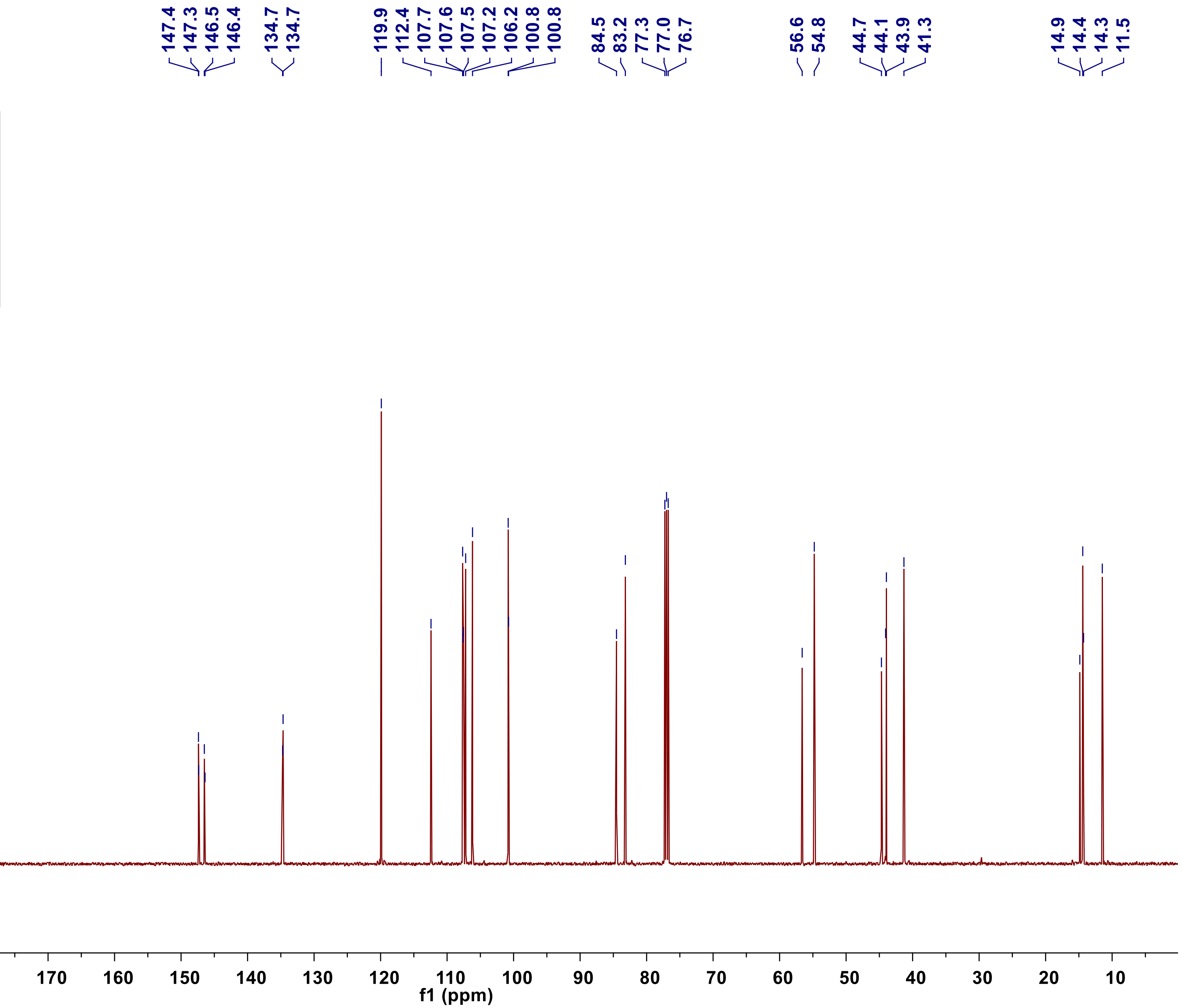

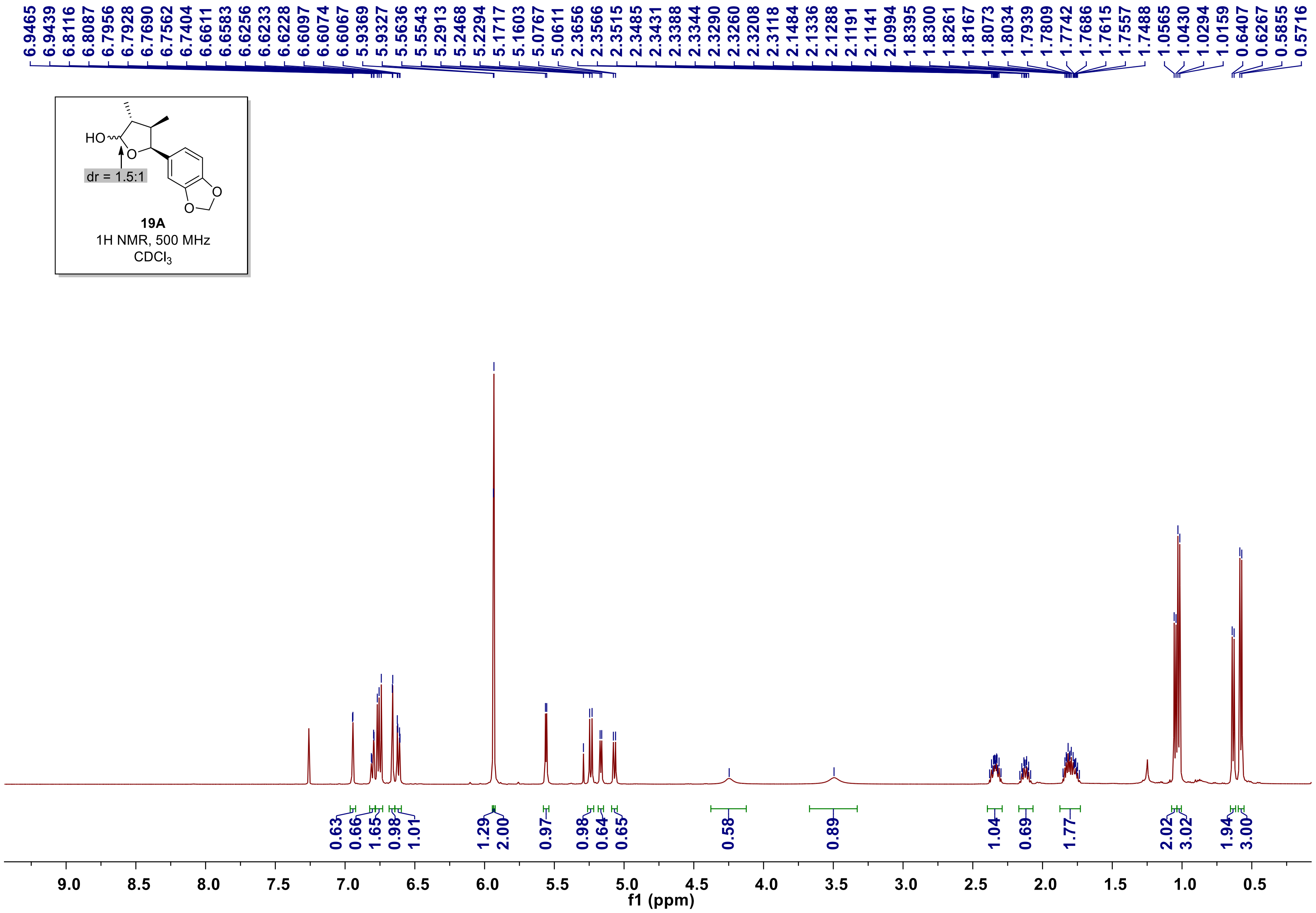

19A
1H NMR, 500 MHz
$CDCl_3$

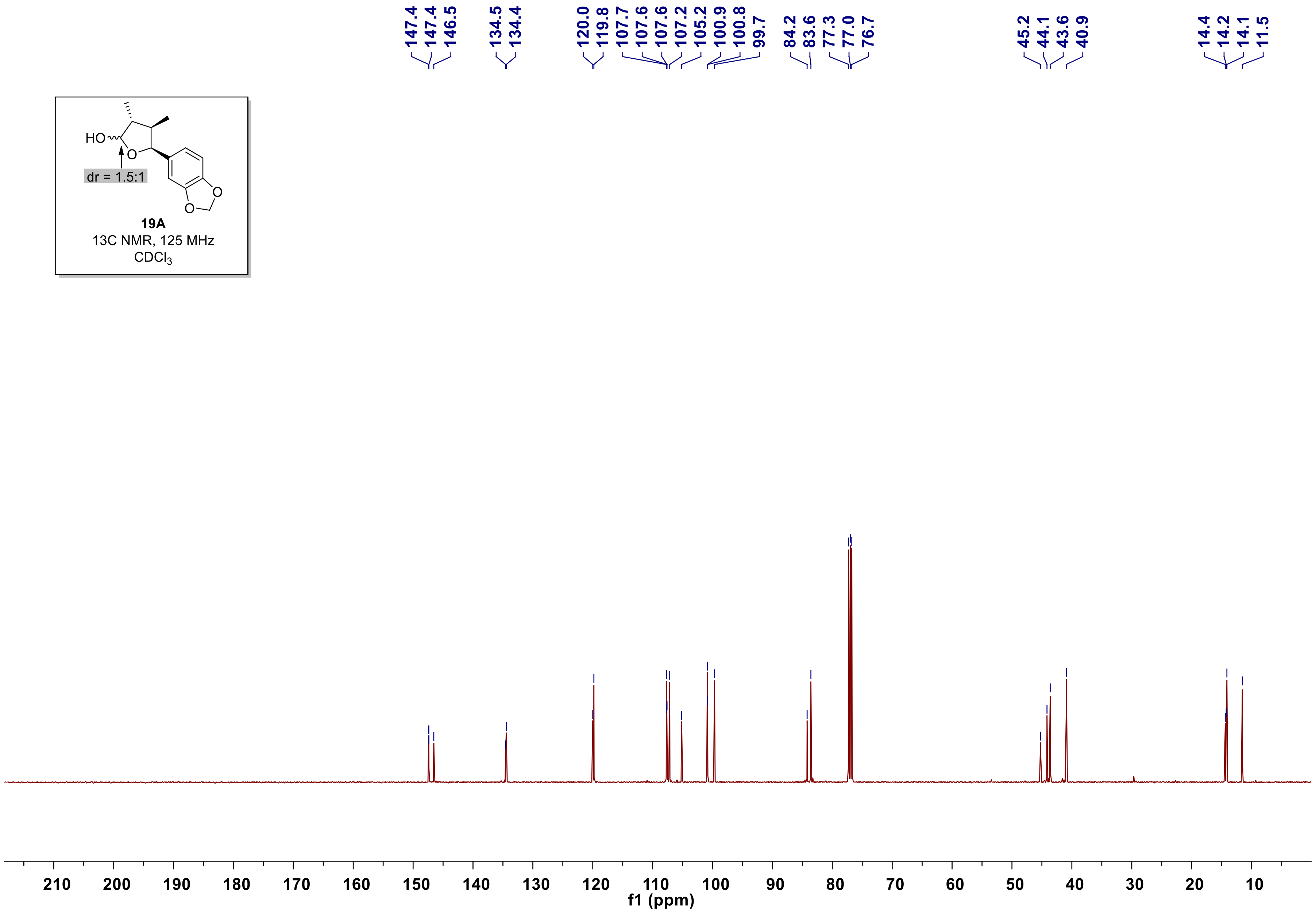
147.4
147.4
146.5
134.5
134.4
120.0
119.8
107.7
107.6
107.6
107.2
105.2
100.9
100.8
99.7
84.2
83.6
77.3
77.0
76.7
45.2
44.1
43.6
40.9
14.4
14.2
14.1
11.5
HO
O
dr = 1.5:1
O
O
19A
13C NMR, 125 MHz
CDCl3
210
200
190
180
170
160
150
140
130
120
110
100
90
80
70
60
50
40
30
20
10
f1 (ppm)

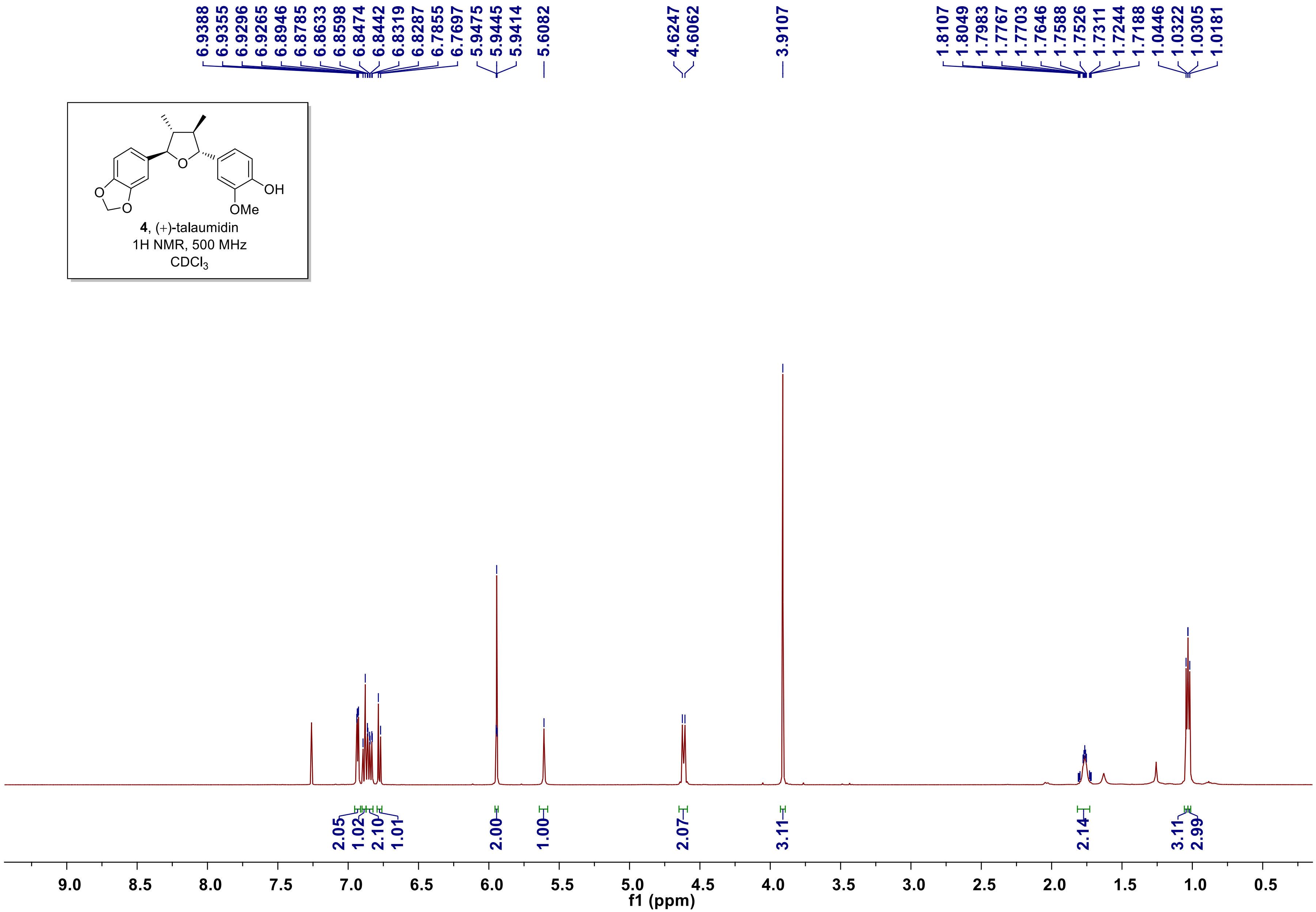
6.9388
6.9355
6.9296
6.9265
6.8946
6.8785
6.8633
6.8598
6.8474
6.8442
6.8319
6.8287
6.7855
6.7697
5.9475
5.9445
5.9414
5.6082
4.6247
4.6062
3.9107
1.8107
1.8049
1.7983
1.7767
1.7703
1.7646
1.7588
1.7526
1.7311
1.7244
1.7188
1.0446
1.0322
1.0305
1.0181
OH
OMe
O
O
O
4, (+)-talaumidin
1H NMR, 500 MHz
CDCl3
2.05
1.02
2.10
1.01
2.00
1.00
2.07
3.11
2.14
3.11
2.99
9.0
8.5
8.0
7.5
7.0
6.5
6.0
5.5
5.0
4.5
4.0
3.5
3.0
2.5
2.0
1.5
1.0
0.5
f1 (ppm)

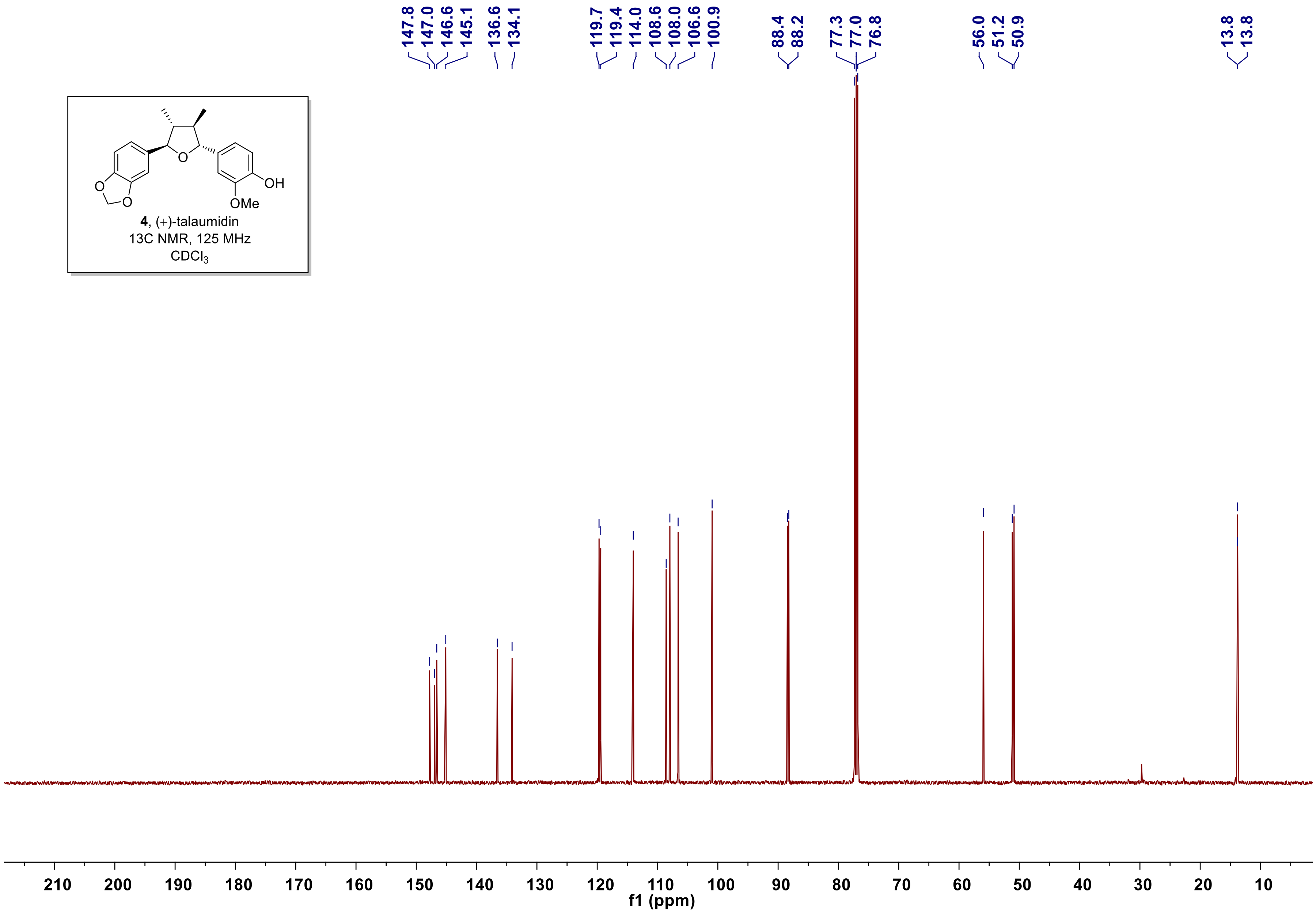
147.8
147.0
146.6
145.1
136.6
134.1
119.7
119.4
114.0
108.6
108.0
106.6
100.9
88.4
88.2
77.3
77.0
76.8
56.0
51.2
50.9
13.8
13.8
OH
OMe
O
4, (+)-talaumidin
13C NMR, 125 MHz
CDCl3
210
200
190
180
170
160
150
140
130
120
110
100
90
80
70
60
50
40
30
20
10
f1 (ppm)

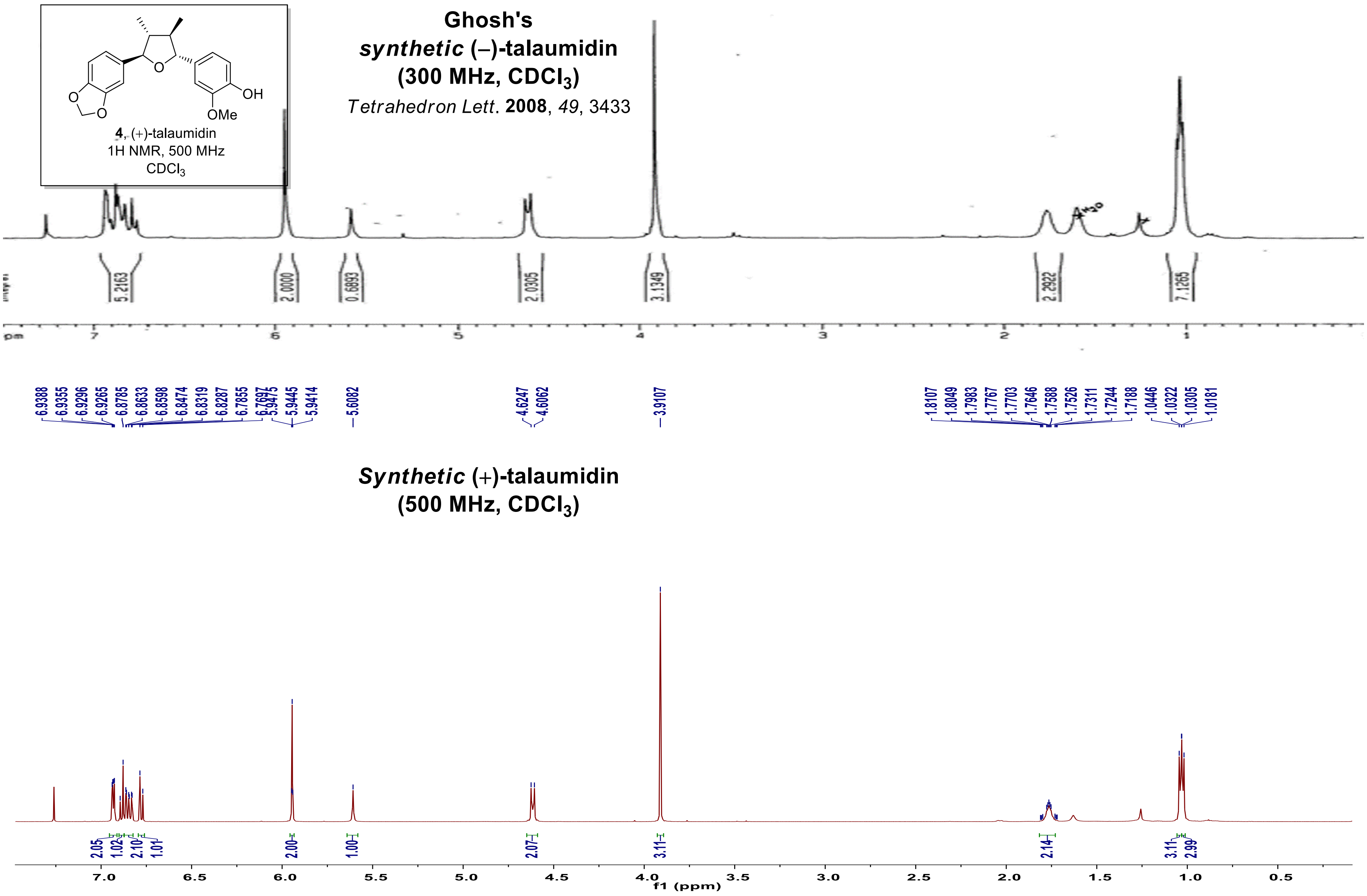

OH
OMe
O
O
O
4, (+)-talaumidin
1H NMR, 500 MHz
CDCl3
Ghosh's
synthetic (–)-talaumidin
(300 MHz, CDCl3)
Tetrahedron Lett. 2008, 49, 3433
5.2163
2.0000
0.6893
2.0305
3.1349
2.2922
7.1265
6.9388
6.9355
6.9296
6.9265
6.8785
6.8633
6.8598
6.8474
6.8319
6.8287
6.7855
6.7697
5.9475
5.9445
5.9414
5.6082
4.6247
4.6062
3.9107
1.8107
1.8049
1.7983
1.7767
1.7703
1.7646
1.7588
1.7526
1.7311
1.7244
1.7188
1.0446
1.0322
1.0305
1.0181
Synthetic (+)-talaumidin
(500 MHz, CDCl3)
2.05
1.02
2.10
1.01
2.00
1.00
2.07
3.11
2.14
3.11
2.99
7.0
6.5
6.0
5.5
5.0
4.5
4.0
3.5
3.0
2.5
2.0
1.5
1.0
0.5
f1 (ppm)

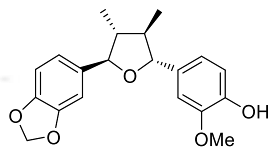

4, (+)-talaumidin
13C NMR, 125 MHz
$CDCl_3$

Ghosh's
***synthetic* (–)-talaumidin**
(75 MHz, $CDCl_3$)
Tetrahedron Lett. **2008**, *49*, 3433

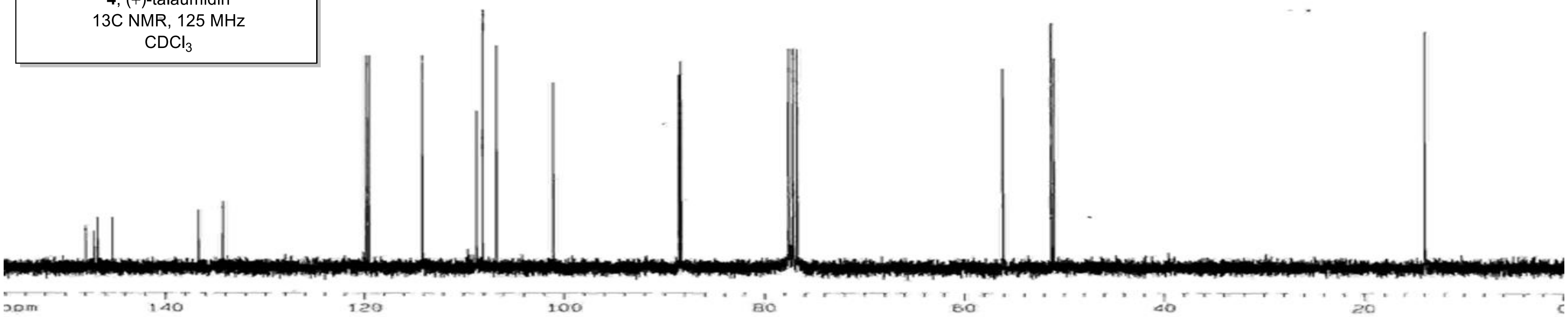

***Synthetic* (+)-talaumidin**
(125 MHz, $CDCl_3$)

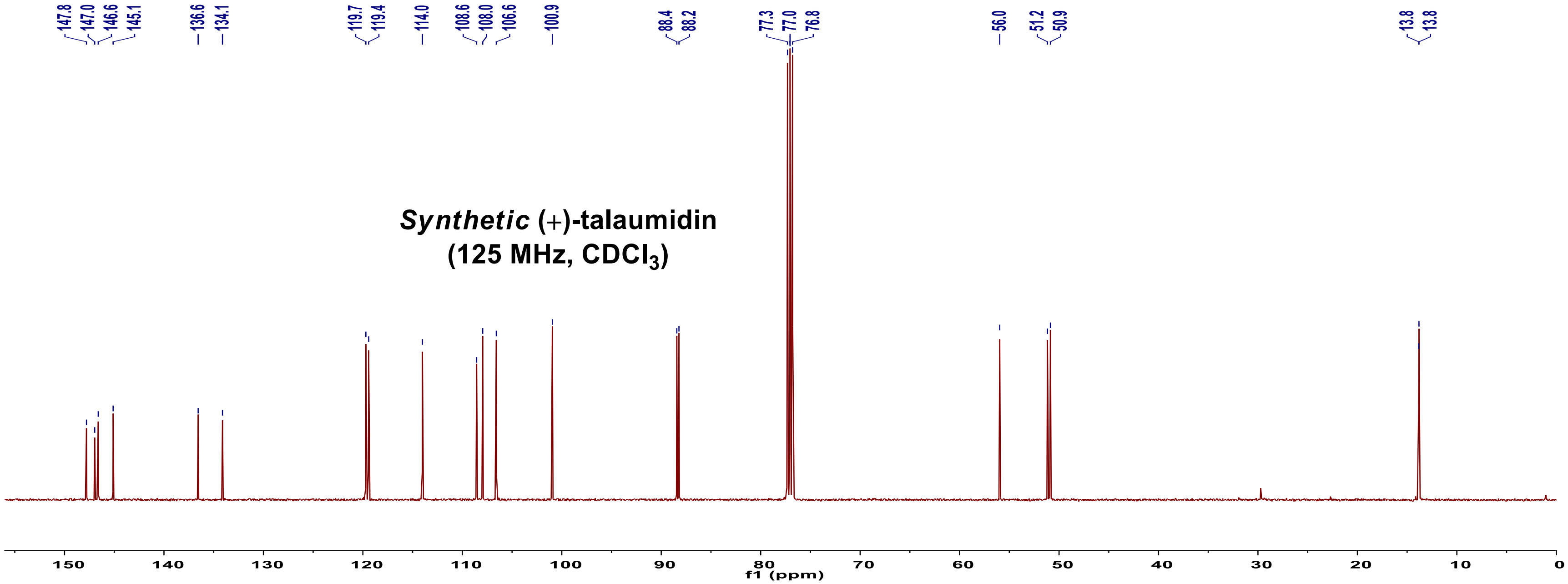

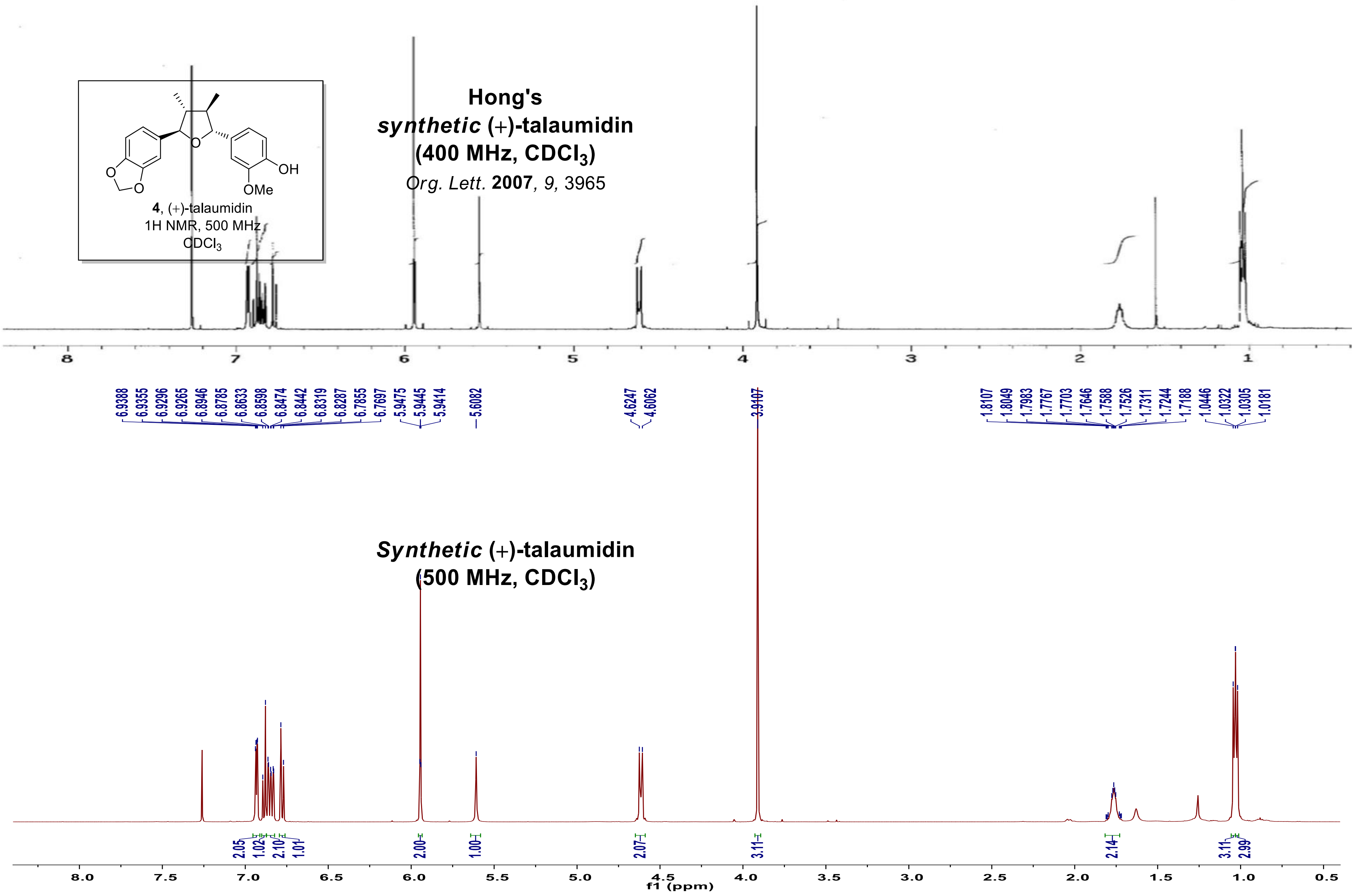
4, (+)-talaumidin
1H NMR, 500 MHz
CDCl3
OH
OMe
Hong's
synthetic (+)-talaumidin
(400 MHz, CDCl3)
Org. Lett. 2007, 9, 3965
Synthetic (+)-talaumidin
(500 MHz, CDCl3)
f1 (ppm)

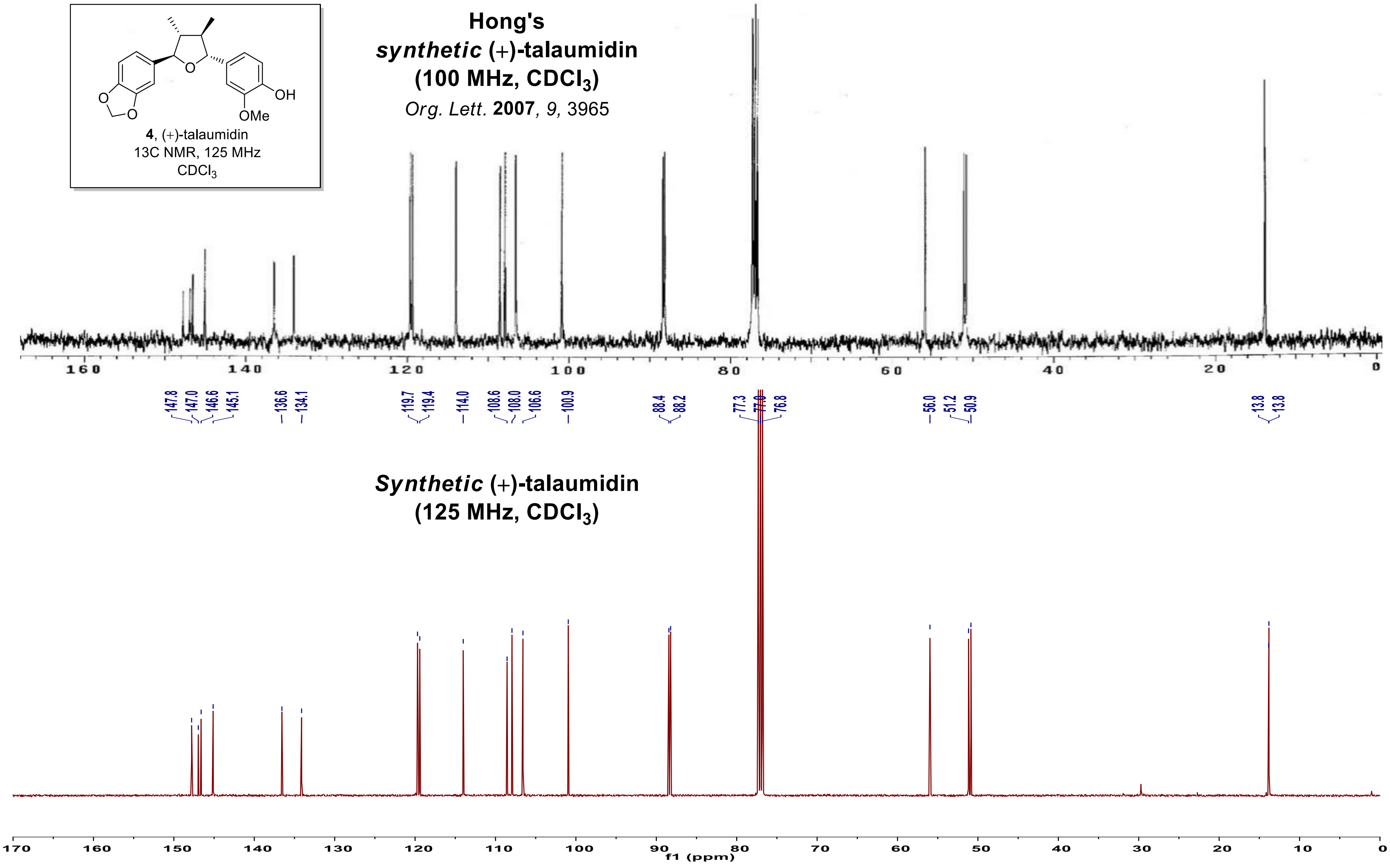

OH
OMe
O
O
O
4, (+)-talaumidin
13C NMR, 125 MHz
$CDCl_3$
Hong's
synthetic (+)-talaumidin
(100 MHz, $CDCl_3$)
Org. Lett. **2007**, *9*, 3965
160
140
120
100
80
60
40
20
0
147.8
147.0
146.6
145.1
136.6
134.1
119.7
119.4
114.0
108.6
108.0
106.6
100.9
88.4
88.2
77.3
77.0
76.8
56.0
51.2
50.9
13.8
13.8
Synthetic (+)-talaumidin
(125 MHz, $CDCl_3$)
170
160
150
140
130
120
110
100
90
80
70
60
50
40
30
20
10
0
f1 (ppm)

Fukuyama's
***synthetic* (+)-talaumidin**
(300 MHz, $CDCl_3$)
J. Org. Chem. **2015**, *80*, 7076

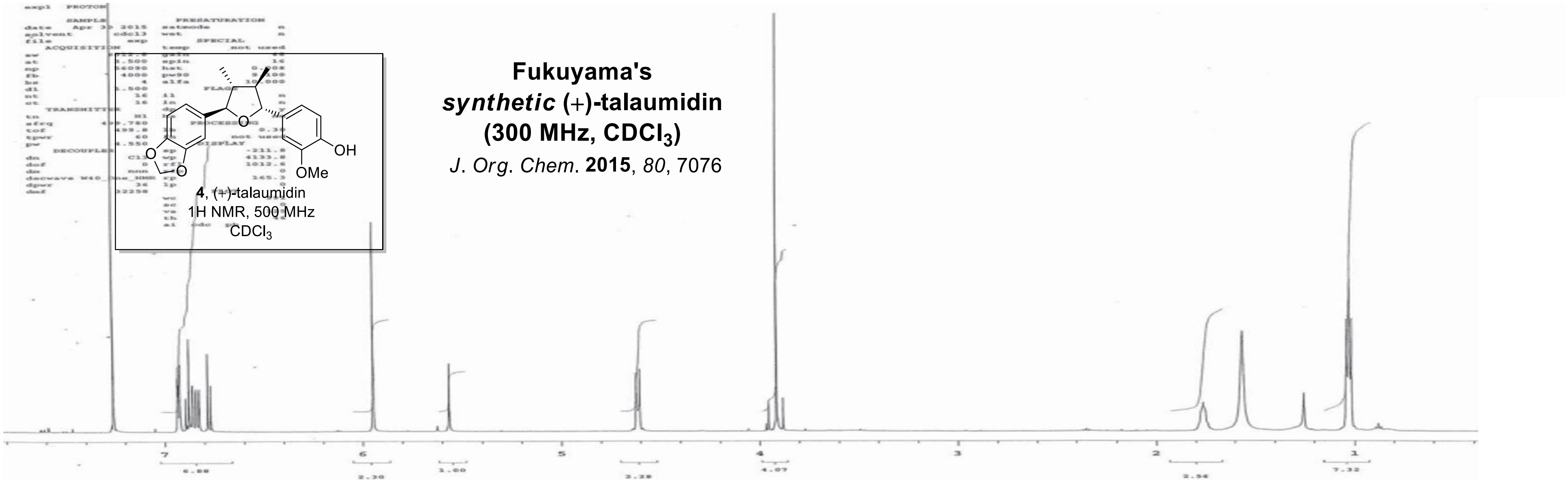

***Synthetic* (+)-talaumidin**
(500 MHz, $CDCl_3$)

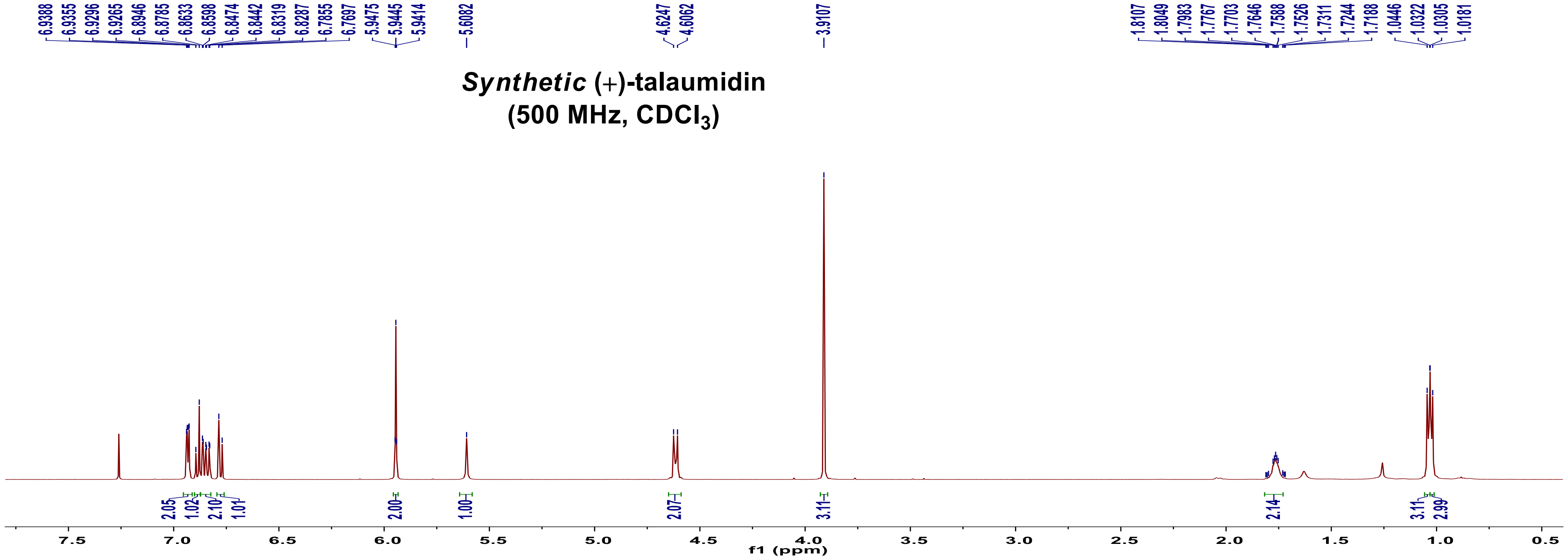

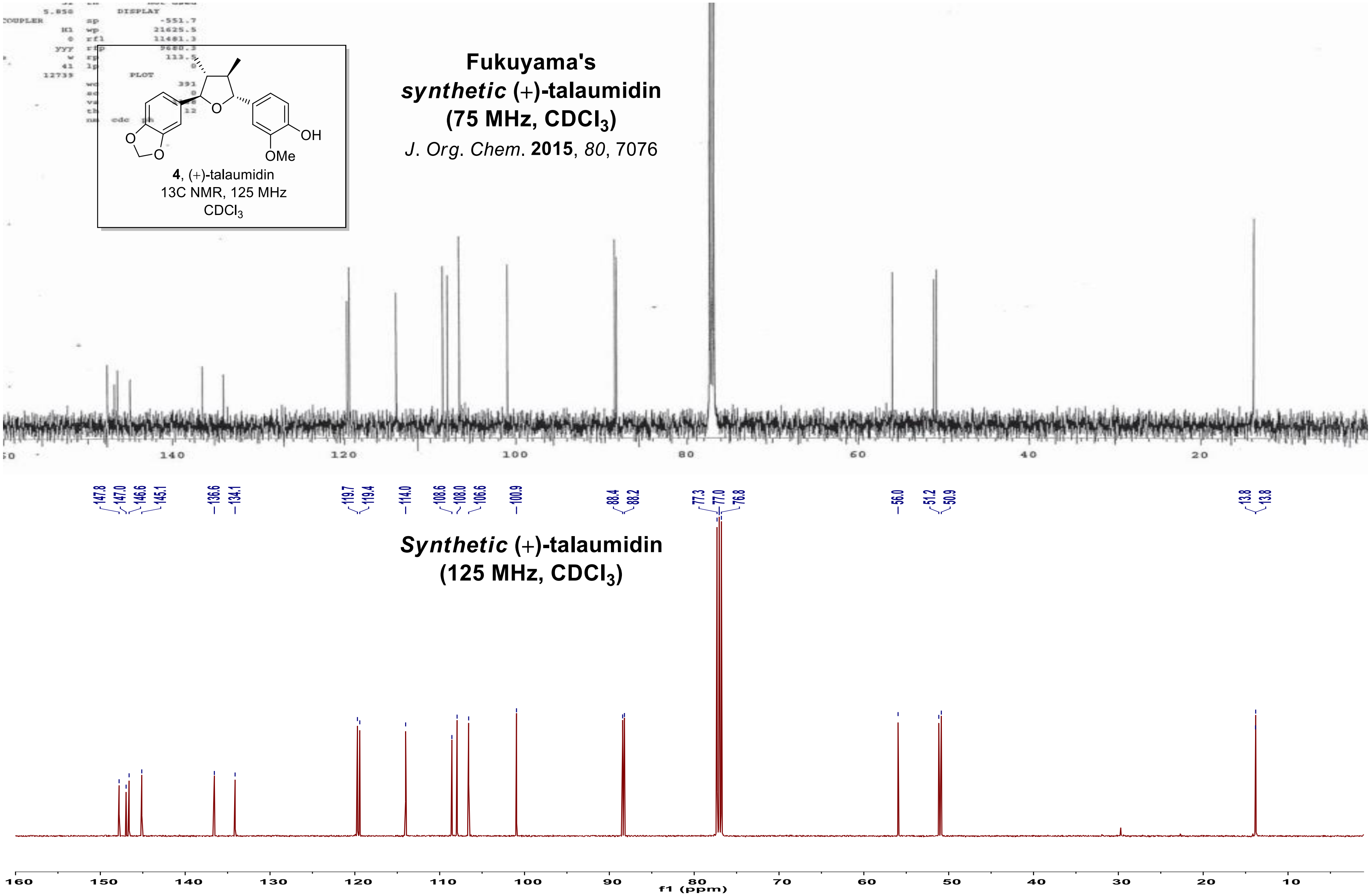
Fukuyama's
synthetic (+)-talaumidin
(75 MHz, CDCl3)
J. Org. Chem. 2015, 80, 7076
OH
OMe
O
4, (+)-talaumidin
13C NMR, 125 MHz
CDCl3
147.8 147.0 146.6 145.1 136.6 134.1 119.7 119.4 114.0 108.6 108.0 106.6 100.9 88.4 88.2 77.3 77.0 76.8 56.0 51.2 50.9 13.8 13.8
Synthetic (+)-talaumidin
(125 MHz, CDCl3)
160 150 140 130 120 110 100 90 80 70 60 50 40 30 20 10
f1 (ppm)

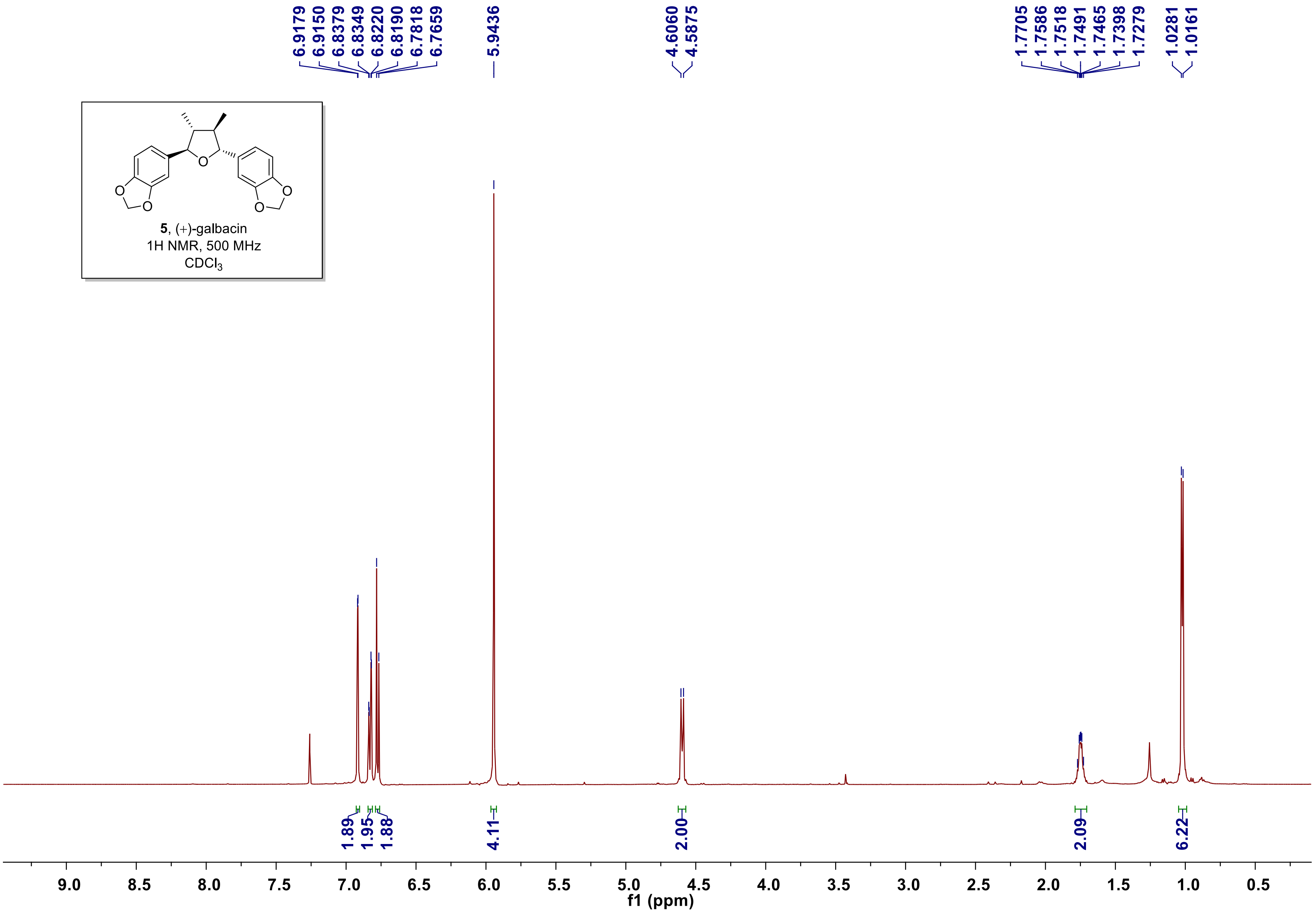
6.9179
6.9150
6.8379
6.8349
6.8220
6.8190
6.7818
6.7659
5.9436
4.6060
4.5875
1.7705
1.7586
1.7518
1.7491
1.7465
1.7398
1.7279
1.0281
1.0161
O
O
O
O
O
5, (+)-galbacin
1H NMR, 500 MHz
$CDCl_3$
1.89
1.95
1.88
4.11
2.00
2.09
6.22
9.0
8.5
8.0
7.5
7.0
6.5
6.0
5.5
5.0
4.5
4.0
3.5
3.0
2.5
2.0
1.5
1.0
0.5
f1 (ppm)

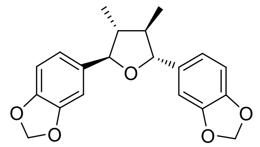

5, (+)-galbacin
13C NMR, 125 MHz
$CDCl_3$

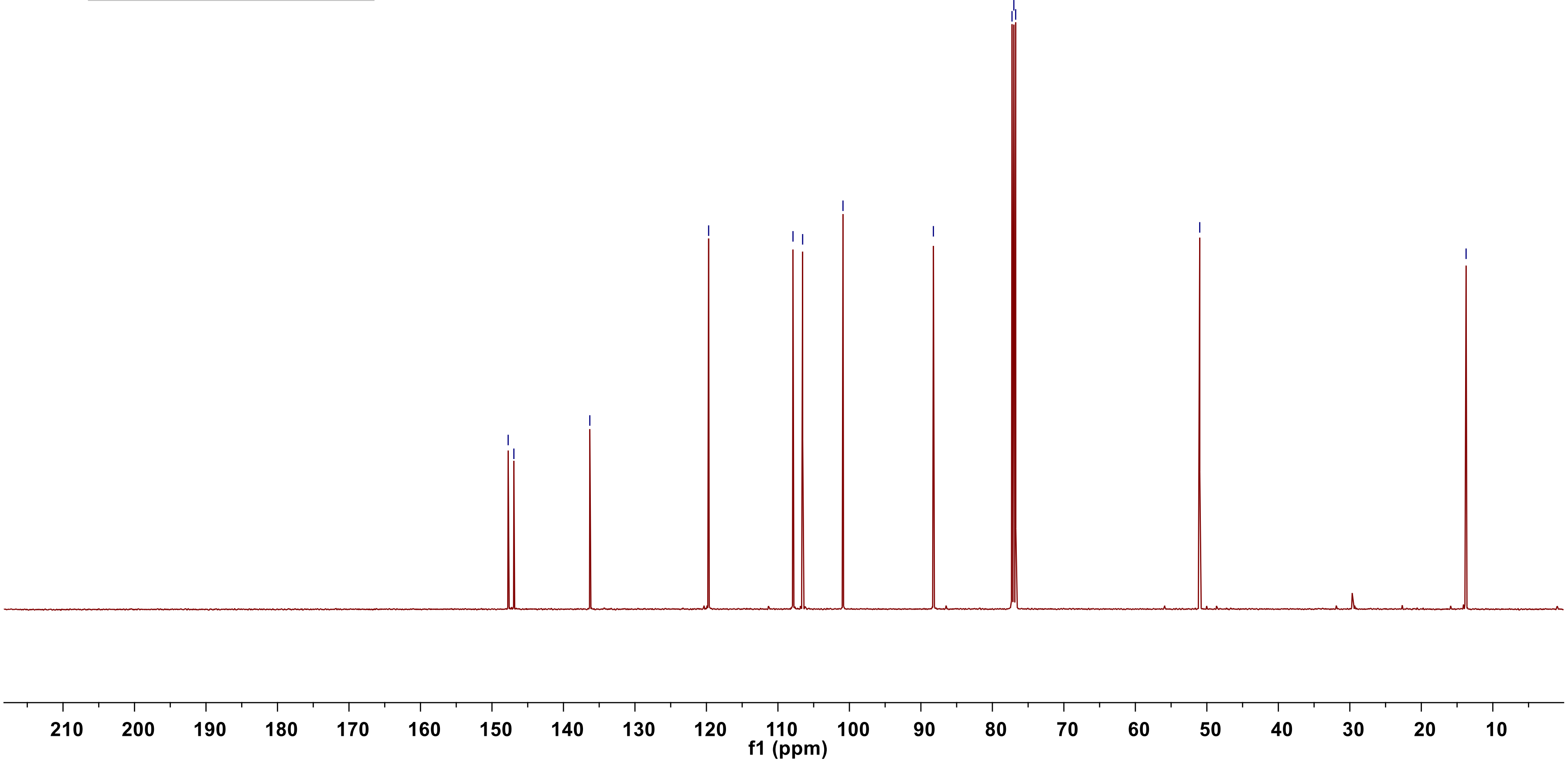

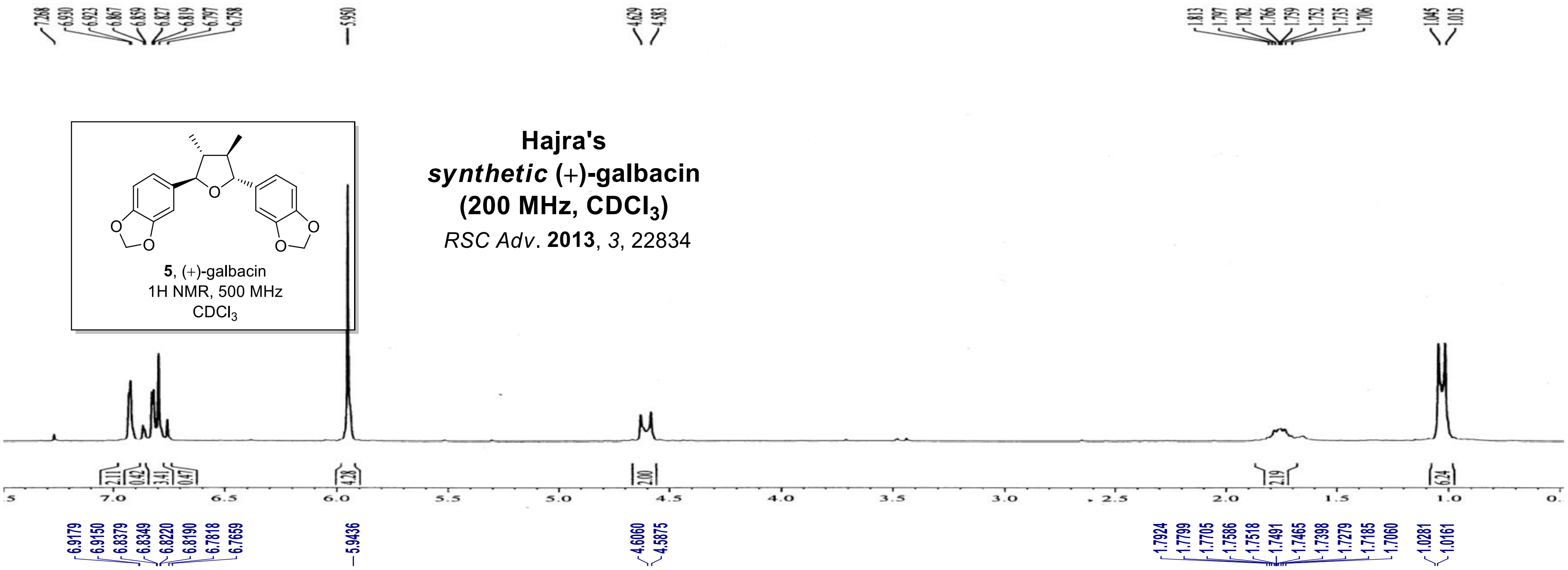

***Synthetic* (+)-galbacin**
(500 MHz, $CDCl_3$)

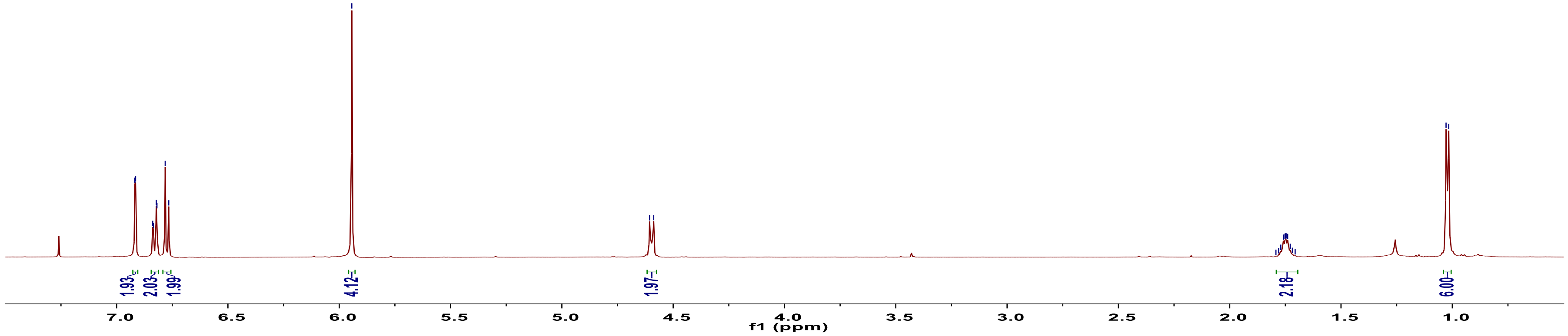

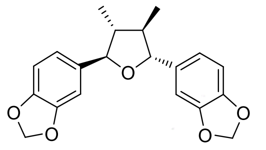

5, (+)-galbacin
13C NMR, 125 MHz
$CDCl_3$

Hajra's
***synthetic* (+)-galbacin**
(50 MHz, $CDCl_3$)
RSC Adv. **2013**, *3*, 22834

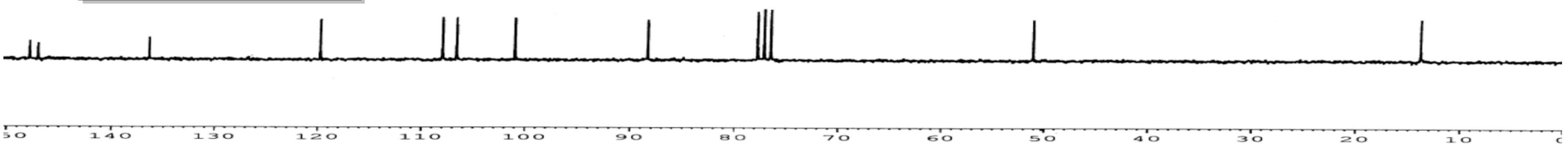

***Synthetic* (+)-galbacin**
(125 MHz, $CDCl_3$)

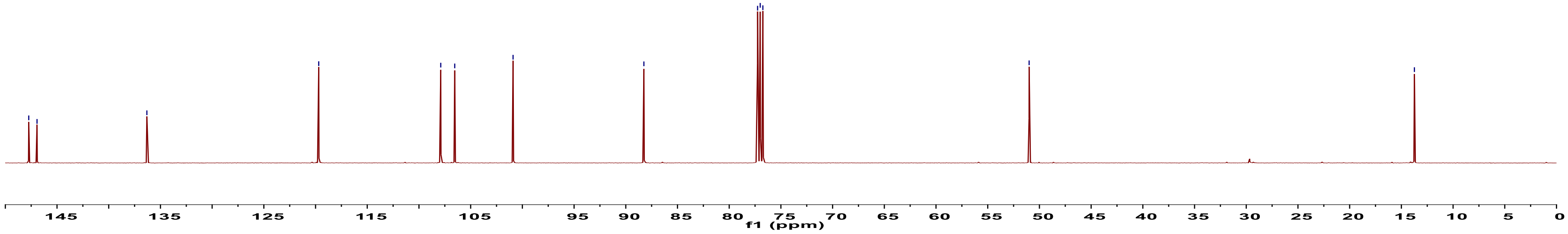

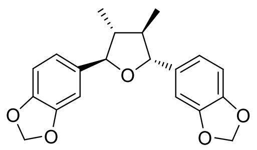

5, (+)-galbacin
1H NMR, 500 MHz
$CDCl_3$

Carreaux's
***synthetic* (–)-galbacin**
(300 MHz, $CDCl_3$)
Org. Biomol. Chem. **2018**, *16*, 1672

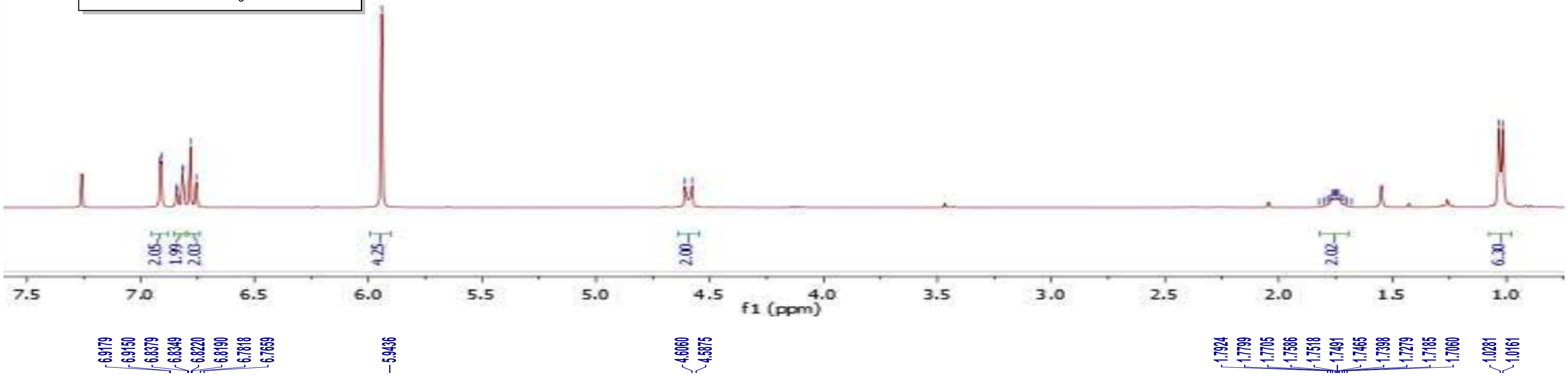

***Synthetic* (+)-galbacin**
(125 MHz, $CDCl_3$)

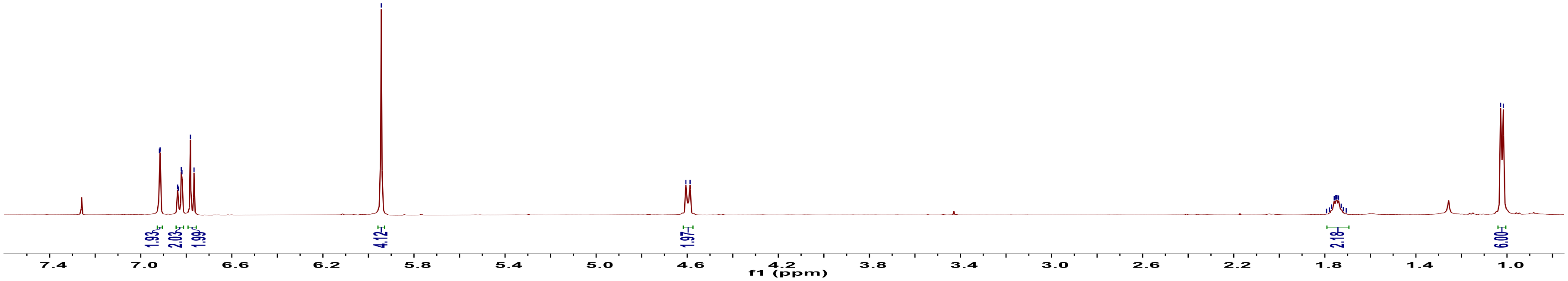

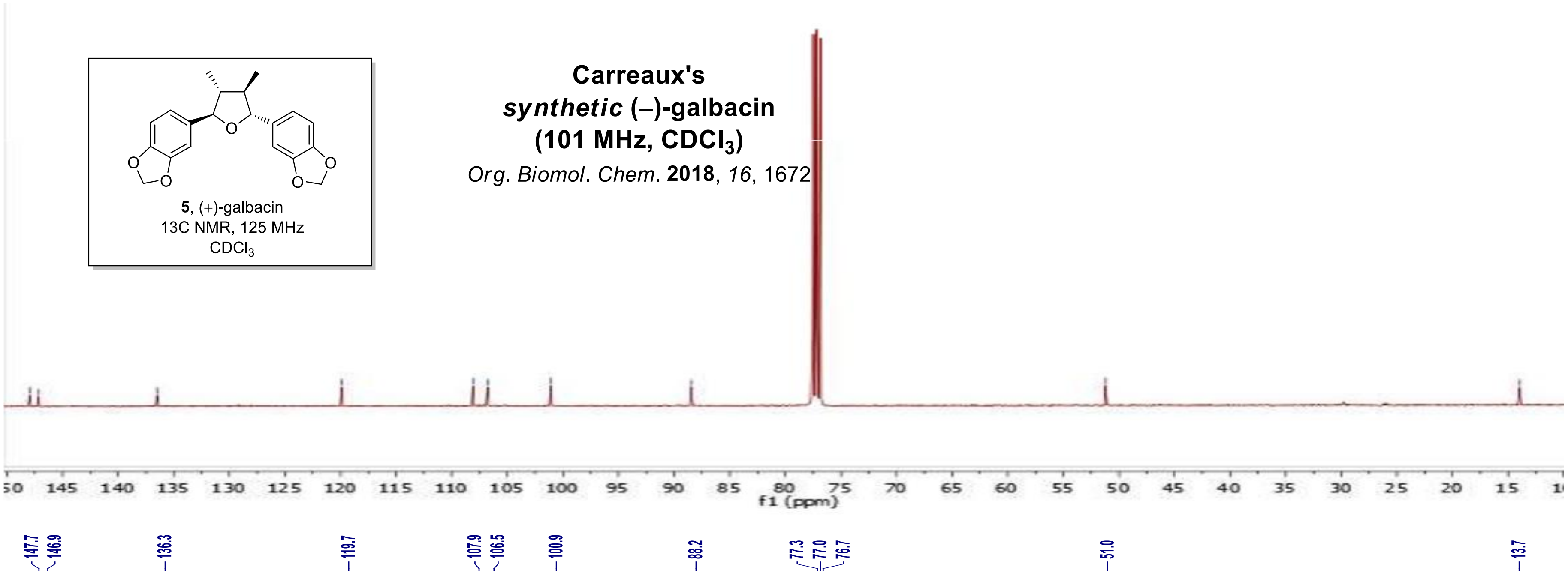

Synthetic **(+)-galbacin**
(125 MHz, $CDCl_3$)

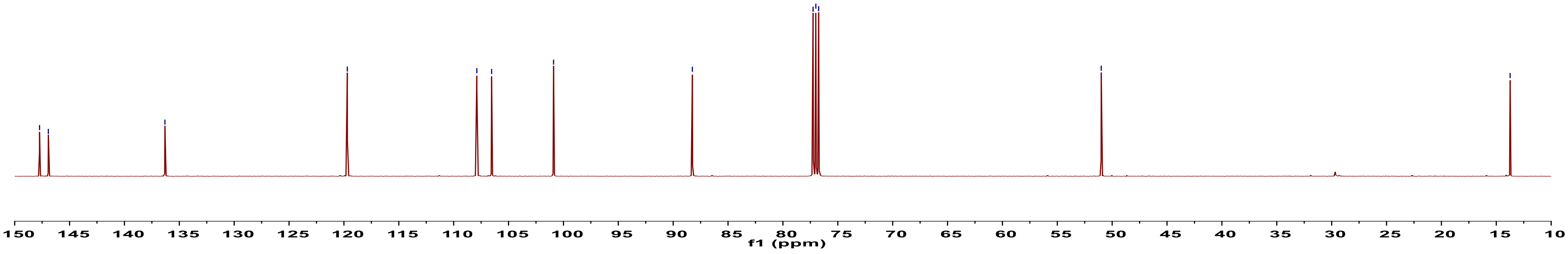

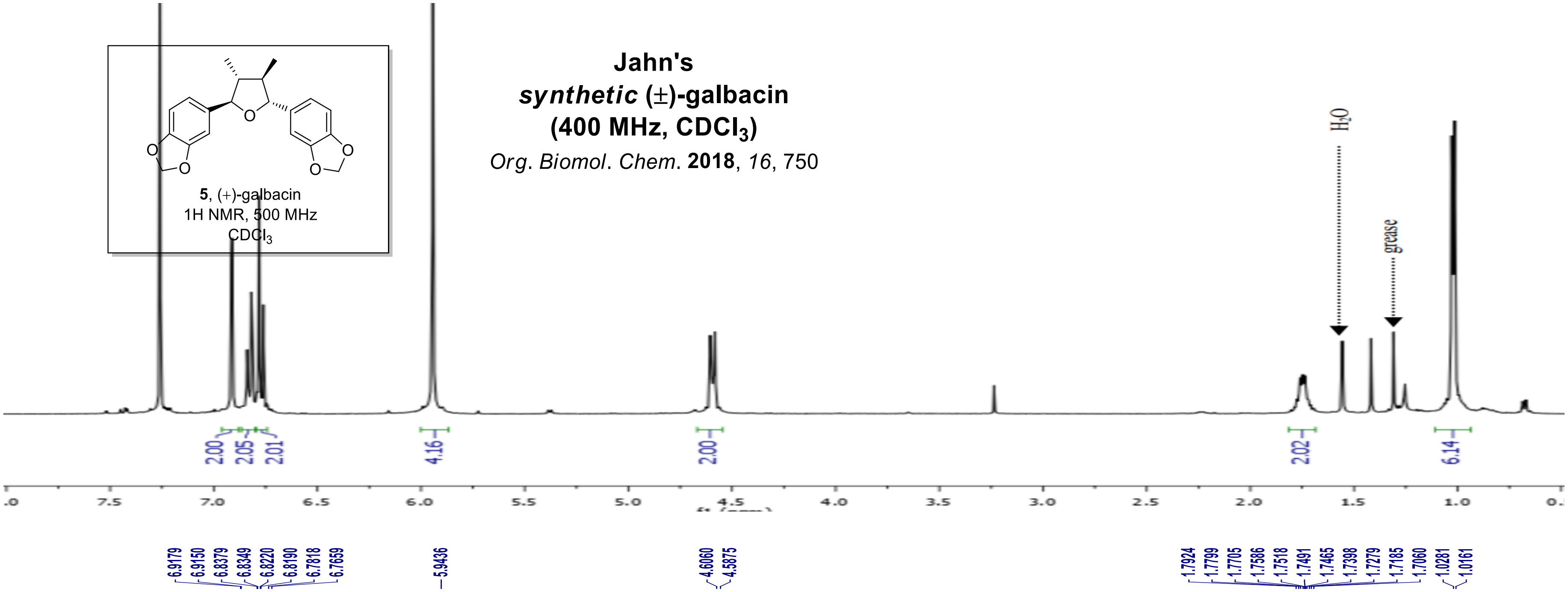

Jahn's
***synthetic* (±)-galbacin**
(400 MHz, $CDCl_3$)
Org. Biomol. Chem. **2018**, *16*, 750

Synthetic (+)-galbacin
(500 MHz, $CDCl_3$)

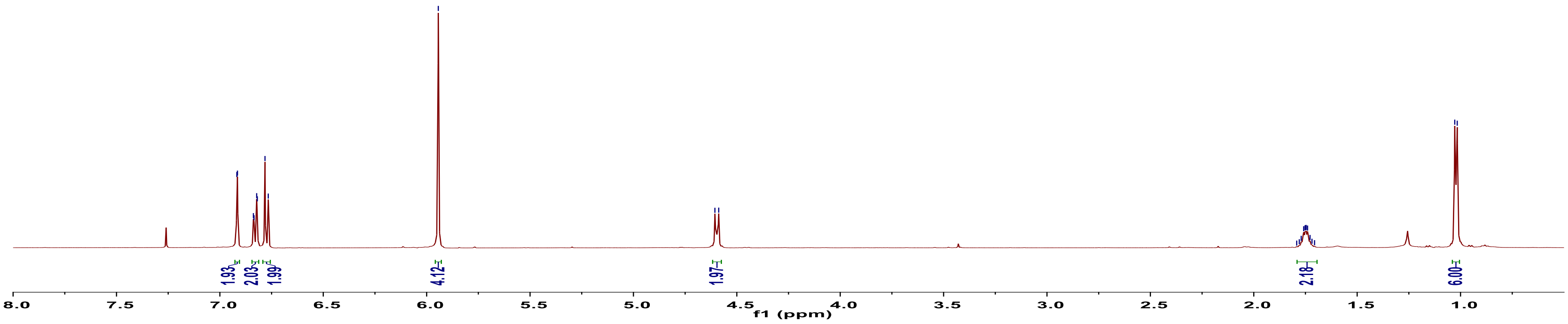

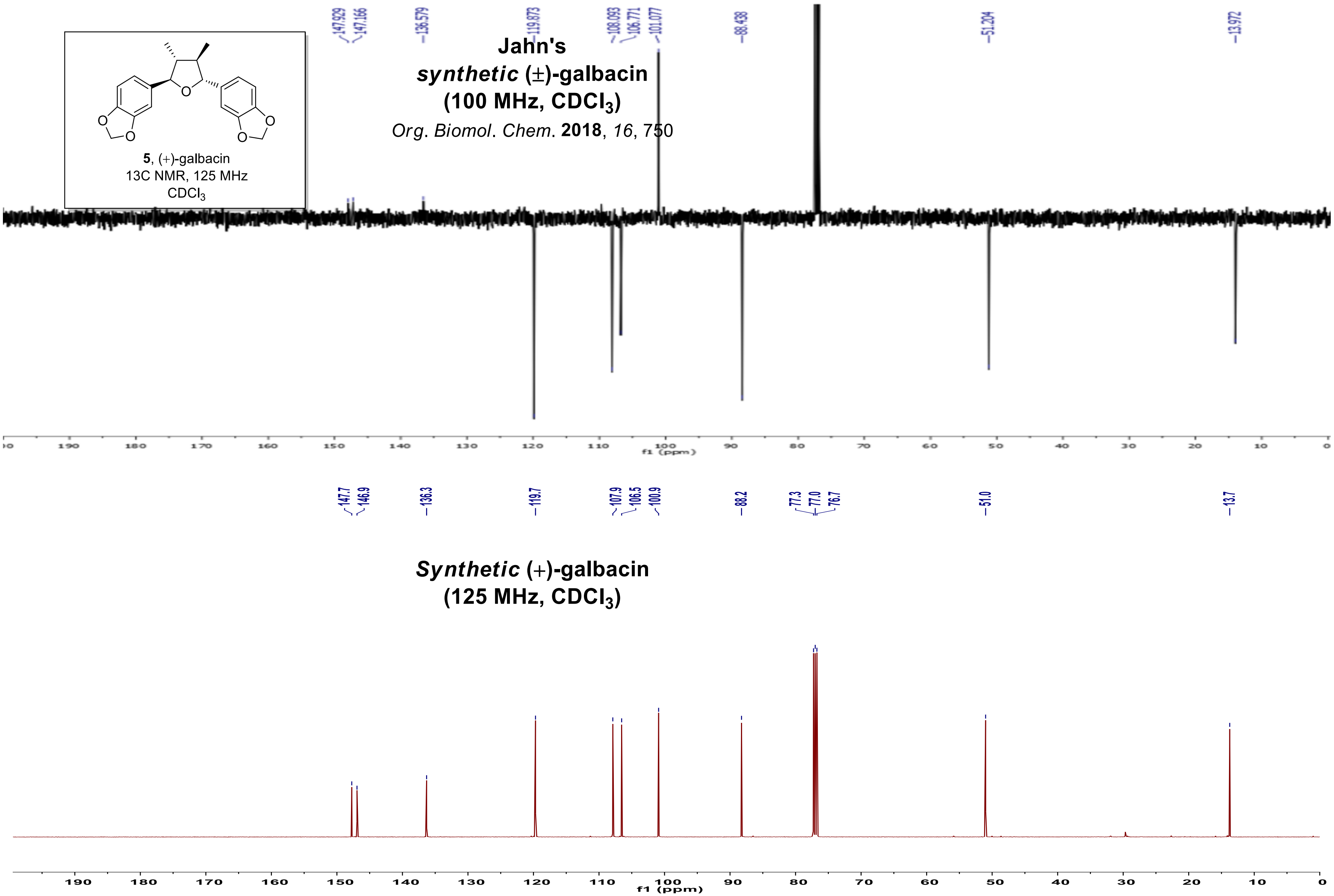
5, (+)-galbacin
13C NMR, 125 MHz
$CDCl_3$
Jahn's
synthetic (±)-galbacin
(100 MHz, $CDCl_3$)
Org. Biomol. Chem. **2018**, *16*, 750
147.929
147.166
136.579
119.873
108.093
106.771
101.077
88.438
51.204
13.972
f1 (ppm)
147.7
146.9
136.3
119.7
107.9
106.5
100.9
88.2
77.3
77.0
76.7
51.0
13.7
Synthetic (+)-galbacin
(125 MHz, $CDCl_3$)
f1 (ppm)

6. Reference

1. Harada, K.; Horiuchi, H.; Tanabe, K.; Carter, R.G.; Kubo, M.; Hioki, H.; Fukuyama, Y.; Esumi, T. Asymmetric synthesis of (–)-chicanine using a highly regioselective intramolecular Mitsunobu reaction and revision of its absolute configuration. *Tetrahedron Lett*. **2011**, *52*, 3005–3008.

2. Harada, K.; Kubo, M.; Horiuchi, H.; Ishii, A.; Esumi, T.; Hioki, H.; Fukuyama, Y. Systematic Asymmetric Synthesis of All Diastereomers of (−)-Talaumidin and Their Neurotrophic Activity. *J. Org. Chem*. **2015**, *80*, 7076−7088.

3. Kim, H.; Wooten, C.M.; Park, Y.; Hong, J. Stereoselective Synthesis of Tetrahydrofuran Lignans via BF_3,OEt_2-Promoted Reductive Deoxygenation/Epimerization of Cyclic Hemiketal: Synthesis of (−)-Odoratisol C, (−)-Futokadsurin A, (−)-Veraguensin, (+)-Fragransin A_2, (+)-Galbelgin, and (+)-Talaumidin. *Org. Lett*. **2007**, *9*, 3965−3968

4. Jagtap, P.R.; Cisarova, I.; Jahn, U. Bioinspired Total Synthesis of Tetrahydrofuran Lignans by Tandem Nucleophilic Addition/Redox Isomerization/Oxidative Coupling and Cycloetherification Reactions as Key Steps. *Org. Biomol. Chem*. **2018**, *16*, 750–755.

5. Jahn, U.; Rudakov, D. Tetrahydrofuran Lignans via Tandem Oxidative Anionic−Radical Processes or Reductive Radical Cyclizations. *Org. Lett*. **2006**, *8*, 4481−4484.

6. Rye, C.E.; Barker, D. Asymmetric Synthesis of (+)-Galbelgin, (−)-Kadangustin J, (−)-Cyclogalgravin and (−)-Pycnanthulignenes A and B, Three Structurally Distinct Lignan Classes, Using a Common Chiral Precursor. *J. Org. Chem*. **2011**, *76*, 6636–6648.

7. Hazra, S.; Hajra, S.A diastereoselective route to 2,5-diaryl-3,4- disubstituted tetrahydrofuran lignans: protection free synthesis of (+)-galbelgin and (+)-galbacin. *RSC Adv*. **2013**, *3*, 22834–22836.

8. Matcha, K.; Ghosh, S.A stereocontrolled approach for the synthesis of 2,5-diaryl-3,4-disubstituted furano lignans through a highly diastereoselective aldol condensation of an ester enolate with an a-chiral center: total syntheses of (−)-talaumidin and (−)-virgatusin. *Tetrahedron Lett*. **2008**, *49*, 3433−3436.

9. Henrion, S.; Macé, A.; Vallejos, M.M.; Roisnel, T.; Carboni, B.; Villalgordo, J.M.; Carreaux, F. Asymmetric synthesis of trans-4,5-disubstituted γ-butyrolactones involving a key allylboration step. First access to (−)-nicotlactone B and (−)-galbacin. *Org. Biomol. Chem*. **2018**, *16*, 1672
